# Cancer-associated fibroblast-derived protein S100-A11 influences the response to anti-HER2 therapies in HER2-positive breast cancer

**DOI:** 10.1016/j.neo.2026.101318

**Published:** 2026-05-19

**Authors:** Melani Luque, Miriam Morales-Gallego, Marta Sanz-Álvarez, Claudia Arguiñano, Yamileth Rangel, Natalia Ramírez-Merino, Mengjuan Qin, Ana Rovira, Joan Albanell, Juan Madoz-Gúrpide, Federico Rojo

**Affiliations:** a Department of Pathology, Health Research Institute-Fundación Jiménez Díaz University Hospital, Universidad Autónoma de Madrid (IIS-FJD, UAM), 28040 Madrid, Spain; b Biomedical Research Networking Centre for Cancer (CIBERONC), 28029 Madrid, Spain; c Department of Pathology, Infanta Elena University Hospital, 28342 Madrid, Spain; d Department of Medical Oncology, Gregorio Marañón University General Hospital (HGUGM), 28007 Madrid, Spain; e Cancer Research Program, Hospital del Mar Research Institute (IMIM), 08003 Barcelona, Spain; f Department of Medical Oncology, Hospital del Mar, 08003 Barcelona, Spain; g Universitat Pompeu Fabra, 08002 Barcelona, Spain

**Keywords:** Breast cancer, HER2-positive, Trastuzumab, Pertuzumab, Resistance, Tumour microenvironment, Cancer-associated fibroblasts, S100-A11, STAT3 signalling, RAGE receptor, Azeliragon, Stattic, Preclinical models, Biomarkers, Therapy-response biomarker potential

## Abstract

Breast cancer is the most commonly diagnosed cancer worldwide and includes the HER2-positive (HER2+) subtype, characterised by HER2 overexpression. HER2+ breast cancer is treated with neoadjuvant anti-HER2 therapy combined with taxane-based chemotherapy, yet a substantial proportion of patients fail to achieve pathological complete response. Increasing evidence indicates that the tumour microenvironment (TME), particularly cancer-associated fibroblasts (CAFs), contributes to therapy resistance through paracrine signalling. In this study, we investigated the role of stromal/CAF-derived S100-A11 in the response to trastuzumab, pertuzumab, and docetaxel (TPD) therapy. Proteomic analysis and ELISA confirmed increased S100-A11 secretion by TPD-treated CAF-200 fibroblasts. Recombinant S100-A11 reduced sensitivity to TPD in multiple HER2+ breast cancer cell lines, whereas S100A11 silencing in CAF-200 attenuated the resistance-promoting effect of CAF-conditioned medium in BT-474 and EFM-192A cells. Mechanistically, S100-A11 exposure was associated with increased STAT3 phosphorylation, and pharmacological inhibition of STAT3 or RAGE partially reversed the S100-A11-associated resistance phenotype. In a reductionist xenograft model, RAGE inhibition with azeliragon attenuated the effect of exogenous S100-A11 on tumour response to TPD. In a retrospective cohort of early-stage HER2+ breast cancer, high stromal S100-A11 expression was associated with residual disease after neoadjuvant therapy and with increased tumour p-STAT3 levels. Dual staining for S100-A11 and α-SMA further supported the presence of S100-A11-expressing CAFs in patient tumours. Together, these findings support a role for stromal S100-A11 in modulating response to anti-HER2 therapy and suggest that the S100-A11/RAGE/STAT3 axis may represent a therapeutically relevant stromal signalling pathway. Further validation in independent clinical cohorts and more physiologically representative models is required.

**Translational relevance:**

Resistance to anti-HER2 therapies remains a major clinical challenge in the treatment of HER2+ breast cancer. This study identifies the stromal protein S100-A11, secreted by CAFs, as a candidate mediator associated with reduced sensitivity to TPD therapy. We show that extracellular S100-A11 promotes tumour cell proliferation and is associated with activation of the RAGE/STAT3 signalling axis. Pharmacological inhibition of this pathway using azeliragon (a RAGE antagonist) or stattic (a STAT3 inhibitor) partially reverses the S100-A11-associated resistance phenotype in vitro and in a reductionist xenograft model. In parallel, high stromal S100-A11 expression is associated with poorer pathological response in a retrospective cohort of early-stage HER2+ breast cancer patients. While these findings are exploratory, they support the potential relevance of stromal S100-A11 as a component of the TME linked to therapy response. Overall, this work provides a rationale for further investigation of the S100-A11/RAGE/STAT3 axis as a potential target in stromal-mediated resistance to anti-HER2 therapy.

**Conceptual advance:**

This study provides further insight into the mechanisms underlying resistance to anti-HER2 therapy by identifying a therapy-associated, stroma-related adaptive response that may contribute to reduced treatment sensitivity beyond tumour-intrinsic alterations. While resistance in HER2+ breast cancer has traditionally been attributed to oncogenic signalling rewiring within tumour cells, our findings suggest that anti-HER2 therapy may also influence CAFs, promoting the secretion of factors that support tumour cell survival under therapeutic pressure. In this context, we describe a paracrine signalling interaction in which CAF-associated S100-A11 is linked to activation of the RAGE/STAT3 axis in tumour cells, accompanied by increased proliferation and reduced sensitivity to treatment. These observations support a model in which the TME acts as an active contributor to adaptive resistance, rather than a passive component. Moreover, the partial reversibility of this phenotype through pharmacological inhibition of RAGE or STAT3 suggests that stromal signalling pathways may represent potential targets for therapeutic intervention. Overall, these findings expand current perspectives on resistance by incorporating the contribution of therapy-associated stromal responses and support further investigation into combinatorial strategies targeting both tumour cells and their TME.

## Introduction

Breast cancer is one of the most prevalent malignancies worldwide and a primary cause of mortality among women [1]. The disease encompasses several subtypes, including human epidermal growth factor receptor 2-positive (HER2+) breast carcinoma, which accounts for approximately 15% to 20% of all reported cases [2]. The distinguishing feature of HER2+ tumours is overexpression of the HER2 receptor, and clinical management is based on anti-HER2 targeted therapies. The standard neoadjuvant regimen for early-stage HER2-positive (HER2+) breast cancer consists of two anti-HER2 antibodies, trastuzumab and pertuzumab, combined with taxane-based chemotherapy such as docetaxel (TPD). This combination has been shown to significantly enhance patient outcomes and survival rates [[3], [4], [5]]. However, nearly 60% of patients fail to achieve a pathological complete response (pCR) following this therapeutic course, thereby increasing the likelihood of recurrence, often as distant metastases developing months or years after surgery [4,6]. Current treatment options for HER2+ metastatic breast cancer remain limited, emphasising the need to prevent disease progression to this stage. Consequently, it is of paramount importance to elucidate the molecular pathways involved in resistance to anti-HER2 therapy and identify therapeutic strategies to overcome resistance in clinical practice.

Recent reports have demonstrated the pivotal role of the tumour microenvironment (TME) in the development of therapeutic resistance in cancer. This dynamic ecosystem fosters tumour growth and survival by enabling mechanisms such as immune evasion, angiogenesis, and metabolic adaptation. Importantly, the TME facilitates the emergence of resistant cancer cell populations through factors such as signalling pathway activation, inflammation, and hypoxia. Among the cellular constituents of the TME, cancer-associated fibroblasts (CAFs) represent the predominant stromal cell population [7,8], playing a central role in sculpting the TME and influencing tumour behaviour through their ability to produce and secrete an array of molecules, including cytokines and growth factors [9,10]. The significance of the CAF-derived secretome [11,12] is increasingly clear, with evidence indicating its contribution to various aspects of breast cancer pathogenesis, including proliferation, tumour progression, metastasis, and the emergence of drug resistance, such as resistance to anti-HER2 therapies [[13], [14], [15], [16], [17], [18]]. Consistent with this, we previously identified CAF-derived FGF5 as a mediator of resistance to anti-HER2 therapy via FGFR2 activation, supporting a role for the stromal secretome in therapeutic failure [15]. Nevertheless, further investigation is needed to fully explain CAF involvement in tumour initiation and progression, particularly in HER2+ breast tumours, and to explore its role in resistance to anti-HER2 therapy. A deeper understanding of these interactions is critical to attenuate therapeutic resistance and improve treatment outcomes.

In a previous study, we used mass spectrometry to analyse the secretome derived from CAFs and identify proteins that might contribute to anti-HER2 therapy resistance. Among these, S100-A11 was identified as the most relevant [19]. S100-A11 belongs to a family of proteins that are increasingly recognised as potentially relevant markers in various tumour types. This relevance stems from the function of S100-A11 in regulating various cellular processes that are key to tumour progression, including its role in the TME [20,21]. S100-A11 involvement in modulating the response to a range of therapies suggests the potential of this protein family as predictive factors of therapy response and as targets for therapeutic strategies [[22], [23], [24]]. However, the molecular mechanisms regulating their functions in breast cancer remain poorly understood, particularly concerning their extracellular role in the TME.

The primary objective of this study was to investigate the involvement of CAF-derived S100-A11 in the response to anti-HER2 therapies in HER2+ breast cancer. *In vitro* assays were conducted to establish the functional role of S100-A11 in resistance development in HER2+ breast cancer cell lines (BCCLs) treated with a combined therapy comprising TPD. These assays included pharmacological modulation of extracellular S100-A11 activity to reduce treatment resistance and improve the efficacy of anti-HER2 therapy. Furthermore, a subcutaneous murine xenograft model using BT-474 cells was employed to validate the role of S100-A11 in treatment resistance *in vivo* and to evaluate the therapeutic effect of pharmacological RAGE (receptor for advanced glycations endproducts) inhibition. Finally, the clinical relevance of S100-A11 as a potential biomarker associated with pathological response to neoadjuvant anti-HER2 therapy in early-stage HER2+ breast cancer was evaluated in patient samples.

## Materials and methods

### Cell cultures and treatments

The human breast cancer cell lines BT-474 (ATCC Cat# HTB-20; RRID:CVCL_0179), AU-565 (ATCC Cat# CRL-2351; RRID:CVCL_1074), SK-BR-3 (ATCC Cat# HTB-30; RRID:CVCL_0033), MCF-7 (ATCC Cat#: HTB-22; RRID: CVCL_0031) and the human T lymphocyte cell line Jurkat (ATCC Cat#: TIB-152; RRID: CVCL_0367) were purchased from the American Type Culture Collection, and EFM-192A (RRID:CVCL_1812) was acquired from the German Tissue Repository DSMZ. BT-474 and MCF-7 cells were maintained in Dulbecco's Modified Eagle’s Medium/Nutrient Mixture F-12 (DMEM/F-12) (Sigma-Aldrich, Steinheim, Germany) supplemented with 10% heat-inactivated foetal bovine serum (FBS) (Gibco, Thermo Fisher Scientific, Waltham, MA, USA), 2 mm glutamine (GlutaMAX, Gibco), and 1% penicillin/streptomycin (P/S) (Gibco). AU-565, SK-BR-3, EFM-192A, and Jurkat cells were cultured in RPMI 1640 (Gibco) with either 10% (for AU-565, SK-BR-3, and Jurkat) or 20% (for EFM-192A) heat-inactivated FBS and 1% P/S. The human cell line CAF-200 was derived from a tissue specimen obtained during breast surgery on a HER2+ patient and normal fibroblast lines NF-31 and NF-39 were obtained from reduction mammaplasties. These three cell lines were immortalised using a retroviral vector expressing hTERT, as previously described [15]. CAF-200 cells were cultured in high-glucose DMEM (Sigma-Aldrich) supplemented with 10% FBS, 2 mm glutamine, and 1% P/S. All cell lines were cultured as monolayers at 37°C in a humidified atmosphere with 5% CO_2_. Mycoplasma contamination was tested in accordance with a previously described protocol [25]. The cell lines were authenticated in accordance with a previously described protocol [25].

The anti-HER2 antibodies trastuzumab, T (Herceptin, Genentech Inc., San Francisco, CA, USA; RRID:AB_3669039) and pertuzumab, P (Perjeta, Genentech Inc.; RRID:AB_3694970) were provided by the pharmacy service of the Fundación Jiménez Díaz Hospital. Treatment concentrations of 15 μg/ml and 20 μg/ml, respectively, were determined based on the research team’s prior experience [25] and findings from the scientific literature. These concentrations reflect levels observed in patient plasma during clinical trials [26,27] and in preclinical models [28,29]. Working concentrations of docetaxel (D), stattic (Selleckchem, Madrid, Spain), and azeliragon (TTP488; MedChemExpress, NJ, USA) were selected based on the IC values determined for each cell line. Drug concentrations were selected according to the experimental context: sub-cytotoxic doses (IC₁₀ range) were used in proliferation assays to minimize non-specific effects, whereas higher concentrations were employed in short-term signalling experiments to ensure effective modulation of the targeted pathway. Recombinant human S100‑A11 protein (S100-A11r), carrier‑free (R&D Systems/Bio‑Techne, Cat. # 9015‑S11‑050; no RRID assigned) was purchased from Bio-Techne R&D Systems (MS, USA) and was reconstituted to a stock concentration of 100 μg/ml in sterile phosphate-buffered saline (PBS) with 0.1% bovine serum albumin (BSA).

### Generation of conditioned medium (CM)

CAF-200 fibroblasts were seeded in T-175 flasks and cultured until reaching approximately 50–60% confluence under standard conditions. Cells were then washed twice with PBS and incubated in fresh complete medium either in the absence (CM [CAF-200]) or presence of T (15 μg/ml), P (20 μg/ml), and D (0.5 nM) (CM [CAF-200/TPD]) for 72 h. For cellular and functional assays, CM was collected directly after the 72 h treatment period without serum starvation to preserve physiological signalling conditions. In all cases, conditioned media were collected and clarified by centrifugation at 500 × g for 5 min to remove cell debris, followed by filtration through a 0.22 μm filter (Millex®-GP, Merck Millipore). Conditioned media were prepared from fibroblasts at comparable confluence and viability across conditions. Residual drug carryover was minimized by PBS washing prior to CM collection. Where indicated, CM was mixed with fresh medium at a 2:1 ratio prior to its application to tumour cells.

### Cell proliferation assays

To ascertain the proliferation rates of BCCLs, cell proliferation assays were conducted in treated 6-well plates. BT-474 and EFM-192A cells were seeded in duplicate at densities of 3.5 × 10^5^ and 2.5 × 10^5^ cells per well, respectively, and allowed to adhere for 24 h in complete medium. The cells were then grown for 5 days in either fresh medium or a 2:1 mixture of conditioned and fresh medium. Subsequently, the cells were administered TPD, S100-A11r, stattic, or azeliragon, as appropriate. The culture media and treatments were replaced after 72 h, except for D, which was maintained only for the initial 72 h due to its high cytotoxicity. Following the treatment, the cells were counted by staining with trypan blue and analysed using the TC20 Automated Cell Counter (all from Bio-Rad, Hercules, CA, USA). Two counts were made for each well, and the mean value was calculated.

### Protein extraction and quantification

Cells were seeded in 6-well plates at a density of 5 × 10^5^ cells per well and allowed to adhere for 24 h. The cells were then treated for 6 h with either vehicle or a combination of 15 µg/ml T and 20 µg/ml P. Following treatment, the cells were rinsed with PBS and total protein extracts were isolated with RIPA buffer containing cOmplete Protease Inhibitor Cocktail tablets and PhosSTOP Phosphatase Inhibitor Cocktail tablets (Roche, Madrid, Spain) at 4°C for 15 min. Finally, lysates were sonicated and clarified (13,000 × g, 10 min, 4°C) and the supernatant was collected. Protein extracts were quantified using the Pierce BCA protein assay kit (Thermo Fisher Scientific) according to the manufacturer’s instructions.

### Western blotting (WB) analysis

Total protein extracts were prepared at a concentration of 1 μg/µl in 4 × Laemmli loading buffer (Bio-Rad) and boiled at 96°C for 15 min. Twenty microliters of the protein extract were loaded into a 10% SDS polyacrylamide gel, and proteins were separated by electrophoresis and transferred to a nitrocellulose membrane (130 V, 90 min, 4°C). Membranes were blocked in 5% milk or BSA in PBST for 1 h and incubated overnight at 4°C under agitation with the following primary antibodies:

  → Snail (C15D3) (rabbit monoclonal antibody, Cell Signaling Technology, Danvers, MA, USA, Cat# 3879; RRID: AB_2255011; 1:500).
  → α-Smooth Muscle Actin, α-SMA (D4K9N) (rabbit monoclonal, Cell Signaling, Cat# 19245; RRID:AB_2734735; 1:1000).
  → Caveolin-1 (D46G3) (rabbit monoclonal, Cell Signaling, Cat# 3267; RRID:AB_2275453; 1:1000).
  → AKT (rabbit monoclonal, Cell Signaling, Cat# 4685; RRID:AB_915783; 1:1000).
  → Phospho-AKT (Thr308) (rabbit monoclonal, Cell Signaling, Cat# 4056; RRID:AB_331163; 1:1000).
  → Phospho-AKT (Ser473) (rabbit monoclonal, Cell Signaling, Cat# 4060; RRID:AB_2315049; 1:1000).
  → p44/42 MAPK (ERK1/2) (rabbit monoclonal, Cell Signaling, Cat# 4695; RRID:AB_390779; 1:1000).
  → Phospho-p44/42 MAPK (ERK1/2)) (Thr202/Tyr204) (rabbit monoclonal, Cell Signaling, Cat# 4370; RRID:AB_2315112; 1:1000).
  → STAT3 (rabbit monoclonal, Cell Signaling, Cat# 30835; RRID:AB_2798954; 1:1000).
  → Phospho-STAT3 (Tyr705) (rabbit monoclonal, Cell Signaling, Cat# 9145; RRID:AB_2491009; 1:1000).
  → RAGE 1 (rabbit monoclonal, Cell Signaling, Cat# 6996; RRID:AB_10830221; 1:1000).
  → S100-A11 [EPR11172] (rabbit monoclonal, Abcam, Cambridge, UK, Cat# ab180593; RRID:AB_2920727; 1:10,000).
  → β-actin (mouse monoclonal, Sigma-Aldrich, Cat# A5441; RRID:AB_476744; 1:5000).

After washing in PBST, membranes were incubated with HRP-conjugated secondary antibodies: goat anti‑rabbit IgG (H + L), HRP (Thermo Fisher Scientific; Cat# 32260; RRID:AB_1965959) or goat anti‑mouse IgG (H + L), HRP (Thermo Fisher Scientific; Cat# 32230; RRID:AB_1965958), both at 1:5000 dilution for 1 h at room temperature. The membranes were then incubated with the detection reagent (Immobilon Crescendo Western HRP substrate, Merck Millipore, Carrigtwohill, Ireland) for 1 min. Signals were visualised using the Amersham Imager 600 system (GE Healthcare, Chicago, IL, USA), and protein band intensities were analysed using densitometry and quantified using ImageJ software (version 1.54d; National Institutes of Health, USA; RRID:SCR_003070). Band intensities were normalized to β-actin and quantified using the Gel Analysis plugin following standard procedures.

### Enzyme-linked immunosorbent assay (ELISA)

The presence of S100-A11 in CM from CAF-200 fibroblasts was quantified using the RayBio Human S100-A11 ELISA kit (RayBiotech, Peachtree Corners, GA, USA). The entire procedure was conducted at room temperature in accordance with the manufacturer's instructions. Briefly, 100 μl of each sample was added in duplicate to a 96-well plate and incubated under shaking. The wells were then incubated with the biotinylated detection antibody, followed by the streptavidin-HRP solution, and then incubated with the TMB substrate in the dark. After the reaction had ceased, the S100-A11 concentration was quantified by measuring absorbance at 450 nm and interpolating the data readings against a standard curve.

### siRNA silencing

Transient silencing of the *S100A11* gene in the CAF-200 line was achieved with the predesigned Silencer Select siRNA targeting human *S100A11* (s12431) and siRNA Silencer Negative Control no 1 (AM4635) (both from Thermo Fisher Scientific). Fibroblasts were seeded in 6-well plates at a density of 2 × 10^5^ cells per well. After cell adhesion, the cells were transfected with 50 nm siRNA and 5 μg of Lipofectamine 2000 (Thermo Fisher Scientific) diluted in Opti-MEM I Reduced Serum Medium (Gibco). Following a 72-h incubation period, transfection was terminated by removing the medium, and the cells were washed with PBS. Subsequently, the cells were trypsinised, pelleted, and a portion of the cell pellet was reserved for RNA extraction and assessment of *S100A11* expression levels. The remaining cells were then reseeded for continued growth up to 10 days post-transfection. During this period, they were cultured in T175 flasks to produce CM in accordance with the previously described protocol [19].

### Quantitative reverse transcription polymerase chain reaction (qRT-PCR)

Total RNA was extracted from the samples using the RNeasy Mini Kit (Qiagen, Hilden, Germany) in accordance with the manufacturer's instructions. The quality of the extracted RNA was assessed using the NanoDrop ND-2000 spectrophotometer (Thermo Fisher Scientific). RNA reverse transcription to cDNA was conducted using the High-Capacity cDNA Reverse Transcription Kit (Applied Biosystems, Waltham, MA, USA). qRT-PCR amplification was performed on the 7500 Fast Real-Time PCR System platform (Applied Biosystems), employing predesigned TaqMan probes specific for *S100A11* (Hs01055944_g1) and *GAPDH* (Hs02758991_g1) (Applied Biosystems). The relative gene expression was calculated according to the comparative delta-delta Ct method [30], using *GAPDH* gene expression levels as a reference.

### Establishment of resistant xenografts in a murine model and preclinical study

An *in vivo* subcutaneous breast cancer xenograft model was developed at the Fundación Jiménez Díaz Hospital. All experiments were performed in accordance with Directive 2010/63/EU and Royal Decree 53/2013 on the protection of animals used for scientific purposes and were approved by the Department of Environment, Agriculture, and Interior of the Community of Madrid. Six-week-old female athymic immunodeficient nude mice (Rj:ATHYM-Foxn1nu/nu*, Janvier Lab, Saint-Berthevin, France) were selected for inoculation. The sample size was initially calculated using power analysis estimates with a significance level of 0.05 and a power of 0.8, along with an additional 20% allowance to account for potential losses. The final sample size was determined on the basis of previous pilot studies that demonstrated consistent tumour growth kinetics and treatment efficacy. Thirty mice were inoculated subcutaneously in their right flank with 20 × 10^6^ BT-474 cells mixed with 1:1 Matrigel (BD Biosciences, Spain) in PBS. When the average tumour volume reached 100 mm^3^, the mice were randomly allocated into six groups of five mice each. Treatment dose and duration were determined based on the findings of prior pilot studies and were administered as follows: Group 1, mice receiving a control treatment consisting of human IgG1ĸ (10 mg/kg, Sigma-Aldrich); Group 2, TPD (concentrations of 10 mg/kg, 10 mg/kg, and 1 mg/kg, respectively); Group 3, S100-A11r (15 µg/kg); Group 4, azeliragon (1 mg/kg); Group 5, S100-A11r in combination with TPD; Group 6, a combination of S100-A11r, TPD, and azeliragon. All treatments were freshly prepared in PBS. For azeliragon, the compound was first dissolved in dimethyl sulfoxide (DMSO) followed by dilution in PBS. This allowed for an injection volume of 100 µl intraperitoneally every three days over a period of four weeks. The mice also received 17β-oestradiol (Sigma-Aldrich) dissolved at 1 µM in drinking water provided ad libitum. Tumour diameters were meticulously measured by two blinded investigators using a digital calliper, and tumour volumes were calculated based on the following formula: volume = width^2^ × length/2. After four weeks, the tumour xenografts obtained from BT-474 cells were excised and included in formalin-fixed paraffin-embedded (FFPE) blocks.

### Immunohistochemistry

FFPE sections of 3 µm thickness were obtained from both human tumour xenografts in mice and patient tumour samples. Immunohistochemistry (IHC) was conducted in accordance with well-established protocols [31]. In summary, FFPE sections were mounted on positively charged glass slides using a Dako Link platform (Dako, Agilent Technologies, Madrid, Spain). Following deparaffinisation, heat-induced antigen retrieval was conducted in a pH 9 EDTA-based buffered solution (Dako), and endogenous peroxidase activity was quenched. Primary antibodies targeting S100-A11 (rabbit monoclonal, Abcam, Cambridge, UK, Cat# ab180593; RRID:AB_2920727; 1:10,000), p-STAT3-Tyr705 (rabbit monoclonal, Cell Signaling, Cat# 9145; RRID:AB_2491009; 1:200), and phospho-Histone H3 (Ser10) (rabbit monoclonal, Cell Signaling, Cat# 9701; RRID:AB_331535; 1:100) were used. Antigen-antibody reactions were visualised through incubation with an anti-rabbit Ig-dextran polymer conjugated with peroxidase (Flex+, Dako) followed by visualisation with 3,3′-diaminobenzidine and counterstaining with haematoxylin. Immunohistochemical staining was conducted using a Dako Autostainer platform. Two senior pathologists (Y.R. and F.R.) conducted a semiquantitative evaluation of the percentage of either CAFs exhibiting positive staining for S100-A11 or tumour cells with positive staining for p-STAT3. In the case of S100-A11, expression assessment was based on cellular localization and morphological criteria, distinguishing stromal (CAF-derived) from tumour cell–associated staining; only S100-A11 expression clearly confined to stromal fibroblasts was considered for CAF-specific scoring, whereas cases with predominant tumour cell staining were evaluated separately and excluded from stromal-focused analyses. Subsequently, an H-score was established, ranging from 0 to 300, as described elsewhere [31].

### Patients and tumour samples

A single-centre retrospective analysis was conducted on 77 cases of HER2+ breast cancer, with an average follow-up duration of 4.58 years. Tumour samples were obtained from the Biobank of the Fundación Jiménez Díaz Hospital in accordance with the provisions of Law 14/2007, of 3 July, on Biomedical Research and Royal Decree 1716/2011, of 18 November. The use of patient samples was approved by the Research Ethics Committee of the Fundación Jiménez Díaz University Hospital. Patients eligible for the study were newly diagnosed with early-stage, localized HER2+ breast cancer, defined as HER2 3+ by immunohistochemistry (IHC) or HER2 2+ with confirmed amplification by fluorescence in situ hybridization (FISH). Eligibility required histopathological confirmation from initial core needle biopsy, including IHC assessment of ER, PR, Ki67, tumour grade, and HER2 status by IHC and FISH when applicable. All patients were candidates for neoadjuvant treatment consisting of TP and taxane-based chemotherapy followed by definitive surgery with available resected tumour tissue for pathological evaluation. FFPE samples containing both the infiltrating tumour and the stromal components were available from diagnostic biopsies for all cases. Clinicopathological data were compiled for each case, and tumour staging was determined according to the TNM classification by the American Joint Committee on Cancer (AJCC) [32], while histological grading followed the Scarff-Bloom-Richardson (SBR) scale modified by Elston [33]. ER, PR, and HER2 expression was assessed by IHC, and HER2 amplification was confirmed by FISH, in accordance with the criteria outlined in the ASCO—CAP guideline [34]. The pathological response was evaluated using the Miller and Payne system, with the appropriate modifications for assessing lymph node response (axillary response) [35].

Clinical data were collected from medical records by oncologists following written informed consent from each patient (Document S1). The studies were conducted in accordance with the ethical guidelines set out in the Declaration of Helsinki. The project was approved by the corresponding ethics committees and institutional review boards of our hospital (Document S2). Clinical tumour response to primary chemotherapy was evaluated using the International Union Against Cancer (UICC) pathological staging criteria [36]. The variables analysed included demographic data (age, menopausal status), pathological features (histological type and grade, oestrogen receptor [ER], progesterone receptor [PR], Ki67), clinical parameters (clinical stage, type of surgery), neoadjuvant treatment received, and treatment efficacy assessed by residual cancer burden index (RCB), clinical response rate, and invasive event-free survival (iEFS).

### Statistical analysis

All statistical analyses were performed using IBM SPSS Statistics (version 27.0.1; RRID:SCR_016479) and GraphPad Prism (version 8.0.1; GraphPad Software, USA; RRID:SCR_002798). Both software packages were used for data processing, statistical testing (including t-tests, Chi-square tests, and ROC curve analysis), and generation of graphical outputs. Data are expressed as mean values ± standard deviation (SD) from at least three replicates, unless otherwise indicated. Normality of data distribution was assessed using the Shapiro–Wilk test, and homogeneity of variances was confirmed with Levene’s test. Comparisons between two groups were conducted using two-tailed Student’s t-tests for parametric data, while associations between categorical variables were assessed using Chi-square tests (without continuity correction). The association between S100-A11 and p-STAT3 as continuous H-score variables was assessed using Spearman’s rank correlation test. In addition, differences in tumour p-STAT3 H-score according to low versus high stromal S100-A11 expression were evaluated using the non-parametric Mann–Whitney U test. The optimal cut-off thresholds for S100-A11 and p-STAT3 expression were determined using receiver operating characteristic (ROC) curve analysis, which identified patients with residual disease after neoadjuvant therapy. Survival outcomes were analysed using the Kaplan-Meier method, and differences between groups were evaluated with the log-rank test to assess statistical significance. Disease-free survival (DFS) was defined as the time from surgery to any signs of relapse, and overall survival (OS) as the time from diagnosis to death from any cause or the last follow-up. Statistical significance was set at *p* < 0.05. No adjustments for multiple comparisons were applied. Statistical analyses and figures were independently reviewed by a qualified biostatistician. The following symbols were used to indicate statistical significance: *: *p* < 0.05, **: *p* < 0.01, ***: *p* < 0.001. The present study was conducted in accordance with the Reporting Recommendations for Tumor Marker Prognostic Studies (REMARK) guidelines [37].

## Results

### Treatment of CAF-200 with TPD increased S100-A11 secretion, influencing the acquisition of resistance to anti-HER2 therapy

**Fig. 1:**
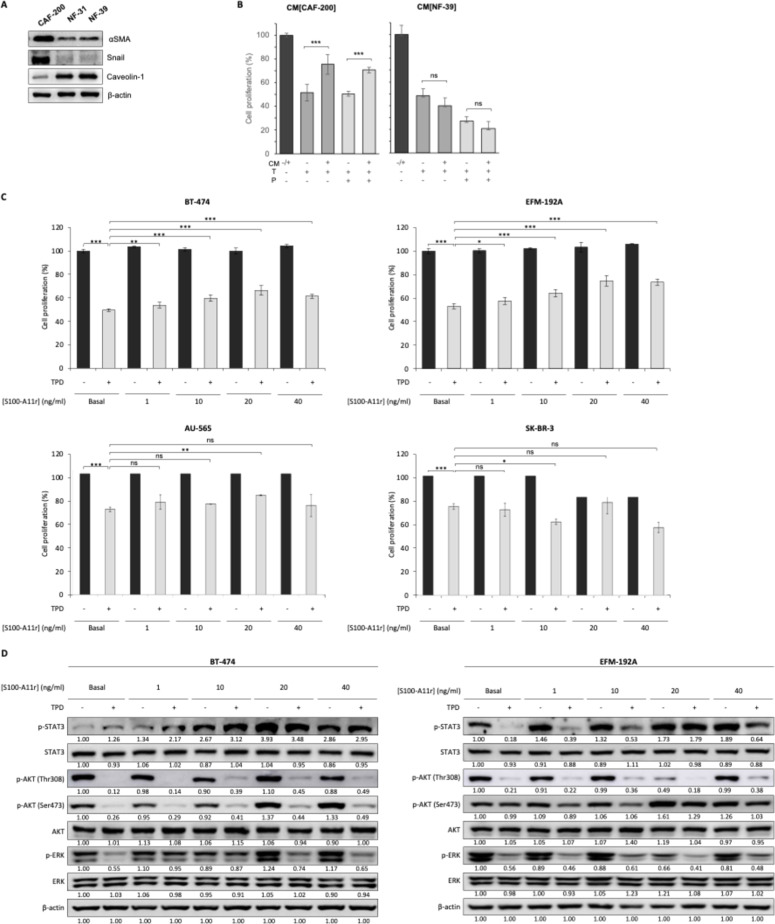
Characterization of CAF-200 fibroblasts and their functional impact on anti-HER2 therapy response. A. Protein expression analysis of fibroblast-associated markers in CAF-200 and normal fibroblasts (NF-31 and NF-39) by WB. CAF-200 displayed a CAF-like phenotype, with differential expression of Snail, αSMA, and caveolin-1 compared with normal fibroblasts. β-actin was used as a loading control. B. Functional impact of CM on BCCL response to anti-HER2 therapy. CM was collected from CAF-200 or NF-39 fibroblasts cultured under basal (untreated) conditions and subsequently applied to tumour cells in combination with TP. CM derived from CAF-200 reduced sensitivity to TP treatment, whereas CM from NF-39 did not reproduce this resistance-promoting effect. Cell proliferation was assessed after 5 days of treatment. Data are presented as mean ± SD (n ≥ 3). Statistical significance is indicated as follows: (*) *p* < 0.05; (**) *p* < 0.01; (***) *p* < 0.001. **C.** Effect of exogenous addition of S100-A11r, ranging from 1 to 40 ng/ml, on proliferation rates of BT-474, EFM-192A, AU-565, and SK-BR-3 cells. **D.** WB analysis of phosphorylated and total forms of STAT3, AKT, and ERK proteins for BT-474 and EFM-192A cells, respectively. The effect of exogenous addition of recombinant S100-A11 was assessed for 6 h.

CAF-200, NF-31 and NF-39 fibroblasts lines were derived from the same experimental framework previously described in our work on CAF-mediated resistance to anti-HER2 therapy [19]. These stromal models had been partially characterized in previous studies [15]. To further define their identity in the present work, we analysed the expression of fibroblast-associated markers. CAF-200 displayed a CAF-like phenotype, with differential expression of markers such as Snail and α-SMA, and lack of expression of caveolin-1 compared with normal fibroblasts, supporting the specificity of the model (Fig. 1A). Functionally, CM from CAF-200 attenuated the sensitivity of HER2+ breast cancer cells to TP, whereas CM from NF-39 did not reproduce this resistance-promoting effect (Fig. 1B). Consistent with this CAF-specific functional activity, previous label-free MS/MS proteomic analysis of conditioned media revealed that TPD treatment increased the abundance of several secreted proteins in CAF-200 compared with both untreated CAF-200 and NF-39 controls [19]. Among these therapy-induced CAF-secreted factors, S100-A11 was previously identified as the most relevant candidate associated with the acquisition of resistance [19]. The concentration of S100-A11 in fibroblast-secreted medium was subsequently confirmed by ELISA using aliquots from the same conditioned media analysed by mass spectrometry, demonstrating a more than three-fold increase in medium derived from TPD-treated CAF-200 compared with control medium from untreated fibroblasts (Figure S1).

To assess the contribution of S100-A11 to treatment resistance, HER2+ BCCLs were exposed to increasing concentrations of S100-A11r under TPD treatment. This analysis included four lines: BT-474, EFM-192A, AU-565, and SK-BR-3. Exposure to S100-A11r resulted in a statistically significant increase in cell proliferation under TPD treatment in three models analysed: in BT-474, EFM-192A and AU-565 cells, the strongest effect was observed upon addition of 20 ng/ml S100-A11r (66.4% vs. 49.5%, *p* < 0.001 for BT-474; 74.7% vs. 53.1%, *p* < 0.001 for EFM-192A; 84.1% vs. 71.8%, *p* < 0.001 for AU-565). More modest resistance-promoting effects were observed in SK-BR-3 cells, although the overall results supported the functional relevance of extracellular S100-A11 across multiple HER2+ breast cancer models (Fig. 1C). Overall, these findings suggest that extracellular S100-A11 contributes to reduced sensitivity to TPD treatment. Because the most consistent and robust S100-A11r–mediated effects were observed in BT-474 and EFM-192A cells, subsequent mechanistic and functional experiments were focused on these two models.

**Fig. 2:**
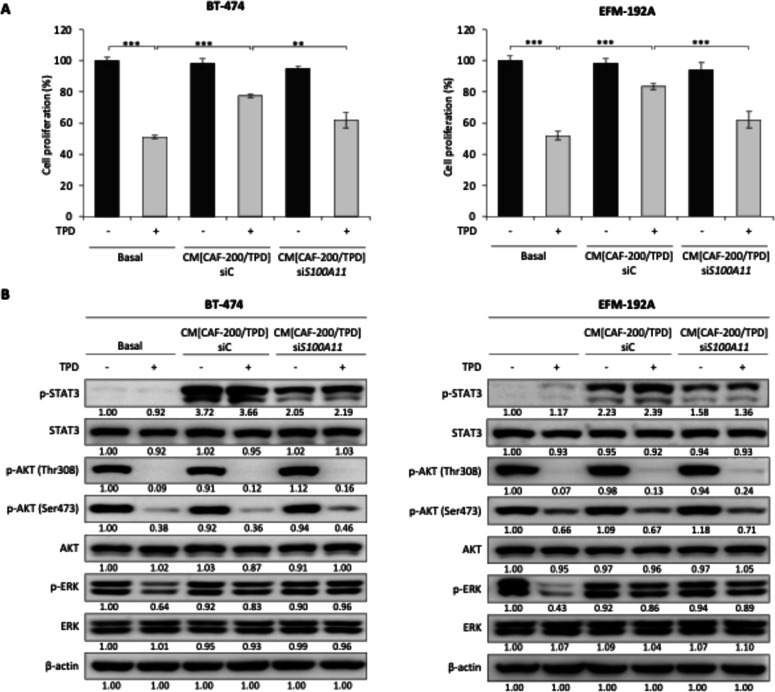
The impact of TPD therapy on HER2+ breast cancer cells is contingent on the presence of S100-A11 in the CM secreted by CAF-200. A. The initial reduction in proliferation rates in the BT-474 cell line by TPD was attenuated by the addition of CAF-200–CM; however, this effect was less pronounced after *S100A11* gene silencing in CAF-200 fibroblasts. CM [CAF-200] was obtained from CAF-200 treated with TPD under the indicated conditions (siC or siS100A11). The same effect was observed in the EFM-192A cell line. Treatment for 5 days with TPD therapy (15 μg/ml T; 20 μg/ml P; 0.5 and 1 nM D, respectively). Basal: control without recombinant protein; CM [CAF-200]: CAF-200–CM; siC: control silencing; si*S100A11*: silencing of *S100A11* gene. (*): *p* < 0.05; (**): *p* < 0.01; (***): *p* < 0.001. Error bars represent the calculated value of the standard deviation (n = 6). **B.** WB analysis of BT-474 and EFM-192A cells, respectively, of phosphorylated and total forms of STAT3, AKT, and ERK proteins. The effect of the CAF-200–CM obtained after *S100A11* gene silencing in CAF-200 fibroblasts was assessed for 6 h. Relative abundance levels of up- or down-regulated proteins were determined by densitometric analysis of the images, normalising them to β-actin loading control and to the respective untreated control. All immunoblot comparisons were performed within the same membrane and exposure conditions; therefore, signal intensities should not be compared across different figures. Representative images are shown for n = 3. Other specific details of the experimental conditions are shown in the Fig. 1 legend, unless otherwise indicated.

To further investigate whether CAF-derived S100-A11 contributes to this effect, the *S100A11* gene was silenced in CAF-200, and the resulting CM was subsequently used to treat BT-474 and EFM-192A cells under TPD therapy. Successful downregulation of S100-A11 mRNA and protein was confirmed by qRT-PCR and ELISA assays, respectively (Figure S2). In these experiments, CM [CAF-200] refers to CM obtained from CAF-200 fibroblasts exposed to TPD treatment, in parallel with siRNA-mediated silencing (siC or siS100A11), thereby modelling therapy-induced stromal signalling (Fig. 2). Notably, the resistance-promoting effect of CAF-200–derived CM was reduced when the cells were exposed to CM [CAF-200 si*S100A11*]. A statistically significant decrease in proliferation was observed in both cell lines (62.0% vs. 77.0%, *p* < 0.01, in BT-474; 62.1% vs. 83.5%, *p* < 0.001, in EFM-192A) when comparing the CM [CAF-200 si*S100A11*] vs. the CM [CAF-200 siC] conditions (Fig. 2A).

### S100-A11–mediated resistance via STAT3 activation

In our previous work [19], we demonstrated that exposing BCCLs to CM from CAF-200 resulted in increased STAT3 phosphorylation. In the present study, we investigated whether the upregulation of this signalling pathway was indeed a consequence of S100-A11. Our findings support this, at least in part. Furthermore, exposure to S100-A11r resulted in elevated p-STAT3 levels in both tumour cell lines, irrespective of the presence or absence of TPD treatment (Fig. 1D). In BT-474 cells, the addition of 20 ng/ml S100-A11r resulted in an increase in p-STAT3 levels of more than three-fold. In EFM-192A, exogenous S100-A11r reversed the initial decrease in STAT3 phosphorylation caused by the treatment, raising p-STAT3 levels above the control. The influence of S100-A11 on p-AKT levels was more moderate, with S100-A11r showing no significant impact on the effects of TPD treatment. In both cell lines, the addition of S100-A11r did not attenuate the dephosphorylation of p-AKT (at either the Thr308 or Ser473 residues) caused by the therapy. Furthermore, S100-A11r had a minimal effect on p-AKT levels in instances where TPD treatment did not modify phosphorylation (at the Ser473 residue in the EFM-192A cell line). In contrast, S100-A11r protein levels exhibited a slight increase in Ser473 phosphorylation at concentrations equal to or greater than 20 ng/ml prior to TPD treatment in both cell lines. The effect of S100-A11r addition on the ERK phosphorylation signal was also quite subtle. In BT-474, it appears that the protein is capable of partially preventing treatment-related dephosphorylation. However, at concentrations of 20 ng/ml or higher, a slight increase in ERK phosphorylation was observed. Conversely, negligible effects were observed in EFM-192A.

To confirm the role of S100-A11 in the generation of acquired resistance to TPD treatment, we examined the effects of the silencing approach at the molecular level (Fig. 2B). The exposure of BCCLs to the CM from S100-A11-depleted CAF-200 reduced the effect on STAT3 phosphorylation, whose levels were reduced in both cell lines, even with TPD treatment. The observation that STAT3 phosphorylation remained significantly elevated relative to the control indicates that S100-A11 is not the sole molecule responsible for this activation. No significant alterations in p-AKT levels were observed when we compared the effect of the CM derived from *S100A11*-silenced CAF-200 with the effect of the medium derived from control fibroblasts. In contrast, the loss of S100-A11 appears to result in the activation of p-ERK.

**Fig. 3:**
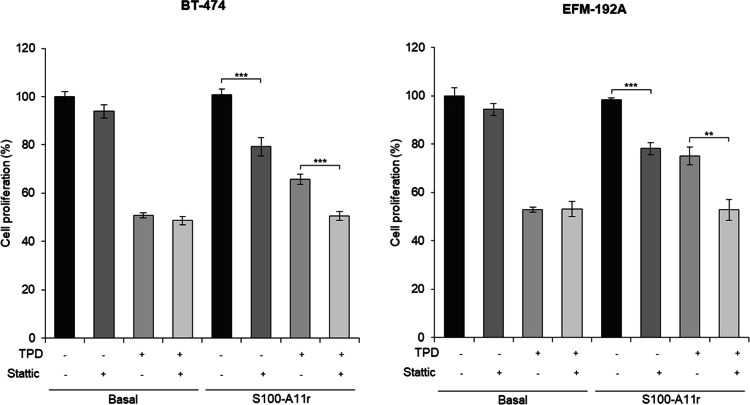
Treatment with stattic reversed BCCL sensitivity to the basal levels of the TPD therapy, despite the addition of the S100-A11r protein. The figure shows the proliferation rates of BT-474 and EFM-192A cells after treatment with stattic, exposed to exogenous S100-A11r (20 ng/ml), with TPD therapy. Other conditions as in previous figures.

The aforementioned findings suggest that STAT3 phosphorylation represents a key downstream signalling event associated with S100-A11 exposure, and thus might be involved in the generation of resistance caused by CAF-200. Consequently, we proceeded to employ stattic, a STAT3 inhibitor, to observe a relationship between STAT3 and S100-A11 (Figure S3). It should be noted that apparent differences in basal p-STAT3 signal intensity between Fig.s 2B and S3 are attributable to differences in image acquisition conditions. In the experiment shown in Fig. 2B, the p-STAT3 signal in samples exposed to CAF-CM was markedly stronger than in basal conditions, requiring shorter exposure times to avoid signal saturation. As a result, basal p-STAT3 levels appear comparatively weaker. In contrast, longer exposure times were used in Figure S3 due to more homogeneous signal intensities across conditions, allowing clearer visualization of basal p-STAT3. Importantly, comparisons were performed within each blot. Once the optimal concentration of the drug was determined (Figure S4), the analysis of cell proliferation under culture conditions with S100-A11r demonstrated a significant decrease following the addition of stattic (79.2%, *p* < 0.001, in BT-474; 78.2%, *p* < 0.001, in EFM-192A) (Fig. 3). While pharmacological inhibition does not fully establish pathway specificity, these findings are consistent with a role for STAT3 signalling in mediating S100-A11–associated resistance. The same effect was observed even in the presence of treatment with TPD (50.5% vs. 65.8%, *p* < 0.001, in BT-474; 52.8% vs. 75.2%, *p* < 0.01, in EFM-192A). These data support a functional contribution of STAT3 activation to the resistance phenotype associated with S100-A11 in the resistance mechanism generated by S100-A11 in HER2+ BCCLs. Consistent with these results, a partial validation in AU-565 cells showed that stattic reduced S100-A11r–associated proliferation under TPD treatment, suggesting that the involvement of STAT3 in this phenotype is not restricted to BT-474 and EFM-192A cells (Figure S5). Similar results were observed with CAF-200–CM, further supporting a functional contribution of CAF-derived S100-A11 to the resistance phenotype (Figure S6).

### RAGE inhibition with azeliragon reversed S100-A11–induced resistance

RAGE (encoded by *AGER*) has been identified as a key receptor for S100 proteins, including S100-A11. Initially, we attempted to assess the functional role of RAGE using siRNA-mediated gene silencing approaches in HER2+ BCCLs. However, transient knockdown using two independent siRNAs yielded inconsistent and non-reproducible suppression of RAGE mRNA expression across cell lines and time points. Therefore, these data were not included, and subsequent analyses focused on pharmacological inhibition using azeliragon as a more robust and reproducible strategy to interrogate RAGE function as a receptor for S100-A11 in the generation of TPD resistance. Azeliragon is a RAGE antagonist capable of inhibiting ligand binding, including S100-A11, and subsequent intracellular transduction through the S100-A11/RAGE/STAT3 axis. Initially, basal expression levels of RAGE in BT-474 and EFM-192A cells were analysed by WB analysis, confirming these to be in the range of positive controls in MCF-7 and Jurkat cells (Figure S7). Based on IC_10_ values, a working concentration of azeliragon was established for each cell line (Figure S8).

**Fig. 4:**
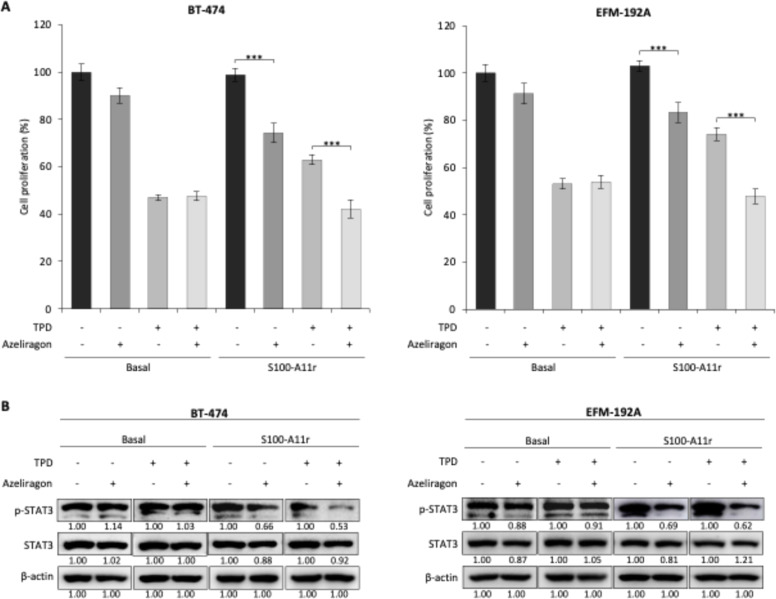
Treatment with azeliragon reversed BCCL sensitivity to the basal levels of the TPD therapy, despite the addition of the S100-A11r protein. **A.** The figure shows the proliferation rates of BT-474 and EFM-192A cells, respectively, after treatment with azeliragon. **B.** The effect of treatment of BT-474 and EFM-192A cell lines with azeliragon for 6 h on the level and activation of STAT3 was evaluated by WB analysis. Other conditions as in previous figures.

Azeliragon treatment reduced the proliferation of both tumour lines cultured with exogenous S100-A11r protein. Specifically, in BT-474 cells, the proliferation rate was reduced to 74.3% compared to the control group (*p* < 0.001), while in EFM-192A cells, it was reduced to 83.2% compared to the control group (*p* < 0.001) (Fig. 4A). This effect was also evident in the presence of TPD, as azeliragon significantly attenuated S100-A11r–induced resistance to TPD treatment (42.1% vs. 63.0%, *p* < 0.001, in BT-474; 47.8% vs. 74.1%, *p* < 0.001, in EFM-192A).

To confirm the role of the S100-A11/RAGE/STAT3 axis in response to TPD therapy in HER2+ BCCLs, we examined the effect of azeliragon on p-STAT3 levels incubated with S100-A11r. As with previous immunoblot analyses, differences in apparent p-STAT3 signal intensity across experiments may reflect variations in exposure times used during image acquisition, which were adjusted to avoid signal saturation and ensure optimal detection within each blot. In these conditions, we observed a decline in STAT3 phosphorylation of 40% to 50% in both BT-474 and EFM-192A cells (Fig. 4B). Similar results were observed with CAF-200–CM, both in proliferation (Figure S9) and at the molecular level (Figure S10), further supporting the hypothesis that S100-A11 derived from the CAF-200 secretome is involved in the induction of treatment resistance. Finally, the abundance and phosphorylation levels of AKT and ERK were analysed after azeliragon treatment, revealing no significant modulation in either cell line (Figure S11).

### *In vivo* validation confirms the therapeutic potential of RAGE inhibition

**Fig. 5:**
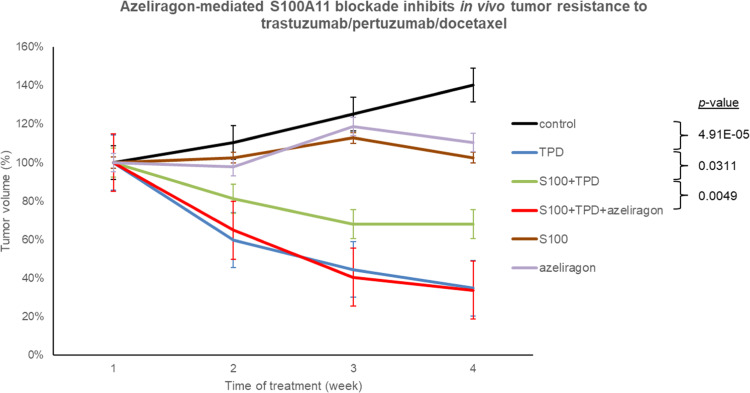
The combination of azeliragon and TPD restored sensitivity to anti-tumour activity in BT-474 cells that had developed resistance to TPD. Azeliragon-mediated S100-A11 blockade inhibited *in vivo* tumour resistance to TPD. Treatment with azeliragon restored sensitivity to TPD: the combination of TPD and azeliragon reduced tumour proliferation and augmented apoptosis in mouse BT-474 xenografts that were resistant to TPD in the presence of S100-A11r. BT-474 cells (20 × 10^6^) were injected subcutaneously into the right flank of the mice. Tumour volume was measured every 3 days after injection until the tumour had grown to ≥ 100 mm^3^. Treatments were injected intraperitoneally every 4 days for 4 weeks. The results are compared with the controls, S100-S11-stimulated tumours, TPD, and the combination of TPD and azeliragon groups of treatment, and the study incorporates a statistical analysis of tumour growth (n = 5 mice/group, two-tailed unpaired-samples t-test).

We assessed azeliragon-mediated restoration of TPD sensitivity *in vivo* using a murine xenograft model. First, the role of S100-A11 in the development of acquired resistance and tumour growth was confirmed. To achieve this objective, the BT-474 cells were xenografted into mice. Once tumour volumes reached a minimum of 100 mm^3^, the animals were randomly allocated to one of six treatment groups: control; TPD; S100-A11r; azeliragon; S100-A11r + TPD; and S100-A11r + TPD + azeliragon. Consistent with *in vitro* findings, the S100-A11r + TPD group exhibited a less pronounced reduction in tumour volume (*p* = 0.0311), which supported our hypothesis that S100-A11 induces resistance to TPD. In contrast, the combination of TPD and azeliragon following the administration of S100-A11r led to a substantial decrease in tumour volume (*p* = 0.0049) that was comparable to the effect of TPD in tumours not stimulated by S100-A11r (*p* = 4.91E-05) (Fig. 5). In addition, longitudinal monitoring of body weight showed stable values across all experimental groups, with no significant treatment-related weight loss, supporting the tolerability of azeliragon in combination with TPD and S100-A11r under the conditions tested (Figure S12). Although this model is based on the administration of recombinant S100-A11 rather than CAF co-implantation, it enables controlled and temporally defined modulation of extracellular S100-A11 levels, thereby allowing direct assessment of its causal contribution to treatment resistance independently of additional stromal-derived factors.

At the conclusion of the experiment, mouse BT-474 xenografts were collected, and markers were analysed by IHC. A study of haematoxylin–eosin-stained biopsy sections confirmed the presence and morphology of BT-474 tumour cells in control mice (treated with IgG alone, S100-A11r, or azeliragon; Figure S13). This revealed the presence of atypical proliferative epithelial cells surrounded by tumour-infiltrating soft tissue. The analysis further revealed the anti-tumour effect of the treatments and demonstrated that human cells were mostly eliminated when mice were given any type of TPD-containing treatment. In addition, we assessed the effects of the combination of TPD and azeliragon on the proliferation of tumour cells. This was achieved by measuring the expression of p-H3 in tumour specimens. The combined treatment resulted in a significant reduction in p-H3 expression (*p* = 0.0240) compared with TPD as monotherapy (Figure S14). Consequently, the BT-474 tumour xenograft rendered resistant by the addition of S100-A11r, and HER2-directed therapy in combination with azeliragon led to a significant inhibition of tumour growth and cell proliferation.

### S100-A11 and p-STAT3 expression analysis in early human HER2+ breast cancer

**Fig. 6:**
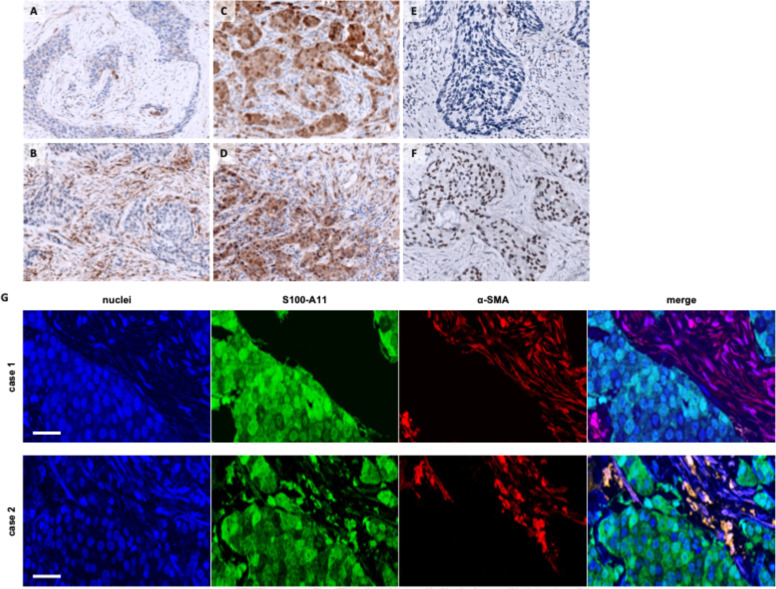
Representative images of the detection of S100-A11 (1:10,000) and p-STAT3 (1:200) by IHC from sections of FFPE samples of HER2+ breast tumours. **A.** Mild, patchy S100-A11 staining in stromal fibroblasts and tumour cells. **B.** Positive S100-A11 staining of moderate intensity in stromal fibroblasts with negative staining in tumour cells. **C.** Diffuse S100-A11 staining of variable intensity in tumour cells with negative staining in stromal fibroblasts. **D.** Positive S100-A11 staining, diffuse in tumour cells and patchy in stromal fibroblasts, of variable intensity. **E.** Negative p-STAT3 staining in tumour cells. **F.** Patchy, moderate-intensity positive p-STAT3 staining in tumour cells. DAB staining; counterstaining with haematoxylin (200 × images). **G.** Identification of S100-A11–expressing CAFs in HER2+ breast tumours by dual immunohistochemistry. Representative dual sequential immunohistochemistry images of S100-A11 and α-SMA in FFPE sections from HER2+ breast tumours. Chromogenic signals were digitally pseudo-coloured in ImageJ for visualisation: S100-A11 (DAB) is shown in green, α-SMA (Permanent Red) in red, and haematoxylin nuclear counterstain in blue. Merged overlays (right panels) highlight areas of co-localisation (yellow signal), indicating S100-A11 expression within α-SMA–positive stromal fibroblasts. Case 1 illustrates a tumour with absence of S100-A11 expression in α-SMA-positive CAFs (S100-A11-low), showing no co-localisation in the merged image. Case 2 shows prominent co-localisation of S100-A11 and α-SMA within stromal cells, consistent with a population of S100-A11–expressing CAFs (S100-A11-high). These findings provide spatial evidence that S100-A11 is expressed in a subset of CAFs within the TME, while also illustrating the heterogeneity of its stromal distribution across cases. Scale bar, 50 µm.

To determine the clinical implications of these findings, we evaluated the prevalence and relevance of CAF-derived S100-A11 and tumour p-STAT3 expression in a retrospective clinical cohort of 77 patients with early-stage HER2+ breast carcinoma (Table S1). All patients had received neoadjuvant treatment consisting of TP and taxane-based chemotherapy. Initially, the expression levels of these two markers were measured in the diagnostic tumour biopsies prior to the initiation of neoadjuvant therapy. Pathologists’ experienced assessment showed S100-A11 expression in both the stromal and tumour compartments in tissue sections. The distribution within the CAFs was heterogeneous, with moderate staining intensity observed in both the cytoplasm and nucleus. S100-A11 expression was also detected in specific mononuclear cells, predominantly lymphocytes and macrophages, as well as endothelial cells within the stroma (Fig. 6A–D). Tumour cells displayed a diffuse staining pattern for S100-A11, ranging from weak to strong intensity, predominantly cytoplasmic with occasional nuclear localisation. p-STAT3 expression was primarily observed in the nuclei of tumour cells, displaying a heterogeneous and patchy distribution, with intensity ranging from weak to strong. Mild cytoplasmic staining was also detected. Within the stromal compartment, nuclear p-STAT3 staining was predominantly observed in lymphocytes, with occasional staining in endothelial and other mononuclear cells (Fig. 6E–F).

Optimal thresholds for S100-A11 and p-STAT3 high expression were determined by ROC curve analysis based on the identification of patients with residual disease after neoadjuvant treatment (Figure S15). Samples with H-score values ≥ 82 (sensitivity = 76%, specificity = 73%) were classified as high S100-A11 expression, whereas samples with H-score ≥ 11 (sensitivity = 84%, specificity = 33%) were considered high for p-STAT3 expression. Notably, the relatively low specificity of the p-STAT3 cut-off may limit its discriminatory capacity when considered as a standalone biomarker. Furthermore, as cut-off values were derived using residual disease as the classification variable, subsequent associations with pathological response should be interpreted as exploratory and hypothesis-generating.

To further address the compartment-specific origin of S100-A11 in human tumours, we performed dual sequential immunohistochemistry on FFPE sections combining S100-A11 and the canonical CAF marker α-SMA. Chromogenic signals were digitally pseudo-coloured to facilitate spatial interpretation. This analysis revealed clear co-localisation of S100-A11 with α-SMA-positive stromal cells in a subset of tumours, identifying a population of S100-A11–expressing CAFs (Fig. 6G). In contrast, other cases displayed α-SMA-positive fibroblasts lacking S100-A11 staining, indicating heterogeneity in CAF-associated S100-A11 expression across tumours. Additional immunofluorescence co-localisation analyses were performed and yielded results consistent with the immunohistochemical findings (not shown). These findings provide direct histological evidence that, although S100-A11 is expressed in multiple cellular compartments, a subset of CAFs clearly expresses S100-A11 in patient samples, supporting its proposed paracrine role within the TME.

The H-score value was determined for all cases, and their distribution was assessed according to the established cut-offs (Figure S16). Overall, 55.8% of cases (43/77) exhibited low S100-A11 expression in CAFs, whereas 44.2% (34/77) showed high expression. Regarding p-STAT3 in tumour cells, 27.3% (21/77) exhibited low expression and 72.7% (56/77) high expression (Table S1).

The relationship between S100-A11 expression in CAFs and p-STAT3 levels in tumour cells was first assessed using continuous H-score values. Spearman rank correlation analysis revealed a significant positive association between both markers (ρ = 0.461, p < 0.001), although the strength of the correlation was moderate, consistent with the different dynamic ranges and cellular compartments evaluated. In addition, when S100-A11 was analysed as a categorical variable, tumours with high S100-A11 expression in CAFs showed higher p-STAT3 levels in tumour cells than tumours with low stromal S100-A11 expression (median p-STAT3 H-score 40 vs. 15; Mann–Whitney U test, p = 0.031). When both markers were classified into low and high expression groups, a significant association was also observed: 91.2% (31/34) of cases with high S100-A11 expression in CAFs exhibited high p-STAT3 levels in tumour cells, compared with 58.1% (25/43) of cases with low S100-A11 expression (p < 0.001) (Table 1; Figure S17). Importantly, the association between stromal S100-A11 and tumour p-STAT3 was consistently observed across both continuous and categorical analyses, reducing reliance on dichotomised ROC-derived thresholds.

Because stromal S100-A11 and tumour p-STAT3 were associated across both analytical approaches, patients were next stratified into two groups according to their combined expression pattern: cases with concomitant high S100-A11 expression in CAFs and high p-STAT3 expression in tumour cells (co-high group), and cases lacking simultaneous high expression of both markers. This combinatorial approach integrates stromal and tumoural compartments and may better capture tumour–microenvironment interactions that are not reflected by individual markers alone. This classification identified a subgroup of patients with a distinct clinical behaviour (Figure S17). Together, these findings reinforce the association between CAF-derived S100-A11 levels and tumoural STAT3 activation and suggest that their combined expression may better capture clinically relevant resistance phenotypes than either marker alone in early-stage HER2+ breast tumours.

### Clinical correlation of stromal S100-A11 and tumour p-STAT3 expression

To explore the clinical relevance of stromal S100-A11 expression, its association with clinicopathological variables was first analysed. High S100-A11 expression in CAFs was associated with more advanced tumour stage (stage III–IV) compared with low S100-A11 expression (38.2% vs. 16.3%, *p* = 0.030). No statistically significant associations were observed with other clinicopathological parameters, including age, tumour size, lymph node involvement, or hormone receptor status (Table 1).

**Table 1:** Correlation between the expression levels of S100-A11 in CAFs and p-STAT3 in tumour cells. The distribution of cases and percentages for each category is presented.

	S100-A11 expression in CAFs				
	Low-level		High-level		χ2 p
	No. cases	%	No. cases	%	
	(n = 43)		(n = 34)		
p-STAT3 expression in tumour cells					
low-level	18	41.9	3	8.8	< 0.001
high-level	25	58.1	31	91.2	

Given the observed association between stromal S100-A11 and tumour p-STAT3 across both continuous and categorical analyses, we next evaluated their potential clinical relevance in relation to treatment response. To evaluate the role of CAF-derived S100-A11 in treatment response, we analysed its association with residual tumour following neoadjuvant therapy, defining pathological complete response (pCR) as the absence of residual tumour in both breast and lymph nodes. High S100-A11 expression in CAFs was associated with pathological response (*p* < 0.001). Specifically, in tumours with high S100-A11 expression in CAFs, 76.5% of cases (26/34) exhibited residual disease, whereas 74.4% (32/43) of cases with low S100-A11 expression achieved pCR, representing a marked absolute difference in response rates. In contrast, p-STAT3 expression alone was not significantly associated with pathological complete response (Table 2), suggesting that its clinical relevance may depend on its integration with stromal S100-A11 expression rather than acting as an independent predictive biomarker.

**Table 2:** Correlation between the expression levels of S100-A11 in CAF-200 and p-STAT3 in tumour cells with tumour and axillary lymph node responses assessed by Miller-Payne and modified Miller-Payne grading system, as well as with pathological response after neoadjuvant treatment. pCR: pathological complete response. The distribution of cases and percentages for each category is shown.

	S100-A11 expression in CAFs					p-STAT3 expression in tumour cells				
	Low-level		High-level		χ2 p	Low-level		High-level		**χ2 p**
	no. of cases	%	no. of cases	%		no. of cases	%	no. of cases	**%**	
	(n = 43)		(n = 34)			(n = 21)		(n = 56)		
Tumor response grade (Miller-Payne)										
G3	4	9.3	10	29.4	< 0.001	5	23.8	9	16.1	0.6
G4	4	9.3	14	41.2		1	4.8	17	30.3	
G5	35	81.4	10	29.4		15	71.4	30	53.6	
Axillary lymph node response grade (modified Miller-Payne)										
Tumor absence (A-D grades)	39	90.7	26	76.5	0.087	18	85.7	47	83.9	0.847
Tumor presence (B-C grades)	4	9.3	8	23.5		3	14.3	9	16.1	
Pathological response										
pCR	32	74.4	8	23.5	< 0.001	13	61.9	27	48.2	0.284
No pCR	11	25.6	26	76.5		8	38.1	29	51.8	

Notably, the co-high group was significantly associated with poorer pathological response, as reflected by a substantially lower proportion of cases achieving pCR (19.4% vs. 73.9%, *p* < 0.001), corresponding to an absolute difference of over 50 percentage points (Table 3). This group also exhibited a higher frequency of intermediate Miller–Payne grades (G3–G4) and a greater proportion of residual tumour in axillary lymph nodes (25.8% vs. 8.7%, *p* = 0.042). These findings support the notion that combined stromal–tumoural biomarker assessment provides a more informative representation of treatment response than either marker alone.

**Table 3:** Correlation between combined expression of S100-A11 in CAFs and p-STAT3 in tumour cells with tumour and axillary lymph node responses assessed by Miller-Payne and modified Miller-Payne grading system, as well as with pathological response after neoadjuvant treatment. Patients were stratified into two groups: high S100-A11 in CAFs and high p-STAT3 in tumour cells (co-high) versus all other expression patterns.

	High expression of S100-A11 in CAFs and p-STAT3 in tumour cells				
	No		Yes		χ2 p
	no. of cases	%	no. of cases	%	
	(n = 46)		(n = 31)		
Tumor response grade (Miller-Payne)					
G3	5	10.9	9	29.0	< 0.001
G4	4	8.7	14	45.2	
G5	37	80.4	8	25.8	
Axillary lymph node response grade (modified Miller-Payne)					
Tumor absence (A-D grades)	42	91.3	23	74.2	0.042
Tumor presence (B-C grades)	4	8.7	8	25.8	
Pathological response					
pCR	34	73.9	6	19.4	< 0.001
No pCR	12	26.1	25	80.6	

Within the entire cohort, only 9.1% of patients (7/77) experienced tumour recurrence. No association was observed between S100-A11 expression in CAFs and recurrence (*p* = 0.942) (Table S2). Kaplan–Meier survival analyses showed no significant differences in disease-free survival (DFS) (*p* = 0.135) or overall survival (OS) (*p* = 0.683) according to S100-A11 expression status (Figure S18). However, these analyses were limited by the low number of events, precluding robust statistical conclusions.

To further explore the potential independent contribution of stromal S100-A11 expression, a multivariable regression analysis was performed including ER status, histological grade, and TNM stage (Table S3). In this model, high stromal S100-A11 expression remained significantly associated with residual disease after neoadjuvant therapy (HR = 3.61, 95% CI 1.71–7.62, *p* = 0.001), whereas ER status, histological grade, and TNM stage were not significantly associated with outcome. These findings suggest that stromal S100-A11 may represent an independent factor associated with residual disease after neoadjuvant therapy in this cohort, although this result should be interpreted cautiously given the limited sample size and number of events.

Although multivariable regression was performed, the clinical analyses should still be interpreted with caution because of the retrospective single-centre design, limited sample size, low number of recurrence events, and lack of external validation. In addition, cut-off values were derived using ROC analysis within the same cohort, which may introduce overfitting and limit the generalizability of these thresholds. Given the number of comparisons performed, the possibility of type I error cannot be excluded.

## Discussion

The TME, and particularly the CAFs within it, has been proposed to play a substantial role in the development of treatment resistance [[38], [39], [40]]. Increasing evidence suggests that improving the efficacy of anti-HER2 therapies and overcoming resistance may be achievable by targeting stromal-derived factors within the CAF secretome, potentially leading to clinically relevant benefits and shifts in therapeutic strategies [39,41]. The primary objective of this study was to identify a candidate factor secreted by CAF-200 fibroblasts, which are known to induce resistance to anti-HER2 therapy via paracrine effects mediated by factors within their secretome [19]. In the analysis, we incorporated comparisons with normal fibroblasts (NF-39), which did not reproduce the resistance phenotype observed with CAF-200, supporting the specificity of the CAF-derived effect. In addition, the resistance-promoting activity of extracellular S100-A11 was validated across multiple HER2+ BCCLs, although with variable magnitude, indicating that the observed effects are not restricted to a single tumour model. The use of a single CAF model (CAF-200) nevertheless represents a limitation of the present study, particularly given the well-recognised heterogeneity of CAF populations across breast tumours. Different CAF subsets may exhibit distinct secretory profiles and functional properties, and therefore the extent to which S100-A11-mediated effects are conserved across CAF subtypes remains to be determined. Validation in additional primary CAF models are in progress to generalise these findings.

We investigated the potential role of the extracellular form of the S100-A11 protein, derived from the secretome of CAFs, in the development of TPD therapy resistance in HER2+ breast cancer. Our findings indicate that S100-A11 contributes to the proliferation of BT-474 and EFM-192A BCCLs. Moreover, the results of this study demonstrate that exogenous S100-A11 reduces the sensitivity of these cells to the combination TPD treatment. Gene silencing of *S100A11* in CAF-200 led to a partial reversal of the therapy resistance induced by fibroblast-derived CM. Importantly, the use of S100A11 silencing in CAF-200 fibroblasts provides functional evidence supporting a stromal origin of the observed effects. CM from S100A11-depleted CAFs showed a reduced ability to induce resistance in tumour cells compared with control fibroblasts, indicating that CAF-derived S100-A11 contributes directly to the resistance phenotype. This functional approach complements the histological findings and supports the relevance of stromal S100-A11 in mediating tumour cell response.

Although the role of S100-A11 protein in cell viability and proliferation in breast cancer cells has not been specifically explored in the literature, evidence regarding its function in tumour cells from other cancer types exists [[42], [43], [44], [45]]. This evidence supports a pivotal function of S100-A11 in the communication between CAFs and tumour cells. The contribution of S100-A11 to the acquisition of drug resistance has been demonstrated in other reports, including studies on chemotherapeutics [46] and radiotherapy [47]. While there is evidence that S100 proteins promote tumour growth and progression by stimulating signalling pathways that regulate cell survival [48], a comprehensive understanding of the mechanistic roles of S100-A11 in breast cancer remains elusive. The secreted S100-A11 acts as an effector molecule, activating intracellular signalling by binding to the RAGE receptor [49,50], though the direct interaction between the two proteins in breast cancer has not yet been elucidated. The interaction of RAGE with S100 proteins, including S100-A11, activates various intracellular signalling pathways, including MAPK, STAT3, and AKT [[51], [52], [53]]. Consequently, the increase in STAT3 phosphorylation observed in BT-474 and EFM-192A cells following incubation with S100-A11r protein might be associated with signalling transduction via RAGE, in a manner analogous to the effect observed in p-STAT3 after the addition of CM in our previous study [19], and consistent with reports in other pathological scenarios [53,54]. The impact of CAF-200–derived S100-A11 on STAT3 phosphorylation was also investigated by *S100A11* gene silencing in CAF-200. This approach resulted in a moderate reduction in STAT3 phosphorylation, while not affecting ERK and AKT phosphorylation, in BT-474 and EFM-192A cell lines. Similar findings were reported in other cancer types [23]. Additionally, we have demonstrated that treatment with stattic could resensitise BT-474 and EFM-192A cells to TPD therapy. In summary, these results indicated that TPD resistance is at least partly attributable to STAT3 activation induced by the S100-A11 protein derived from CAF-200, and that it is susceptible to reversal through direct intervention in this signalling pathway. Functional assessment in this study was primarily based on short-term proliferation assays. While these experiments provide a robust and reproducible measure of treatment response, they do not capture other relevant aspects of tumour cell behaviour, such as apoptosis, long-term clonogenic survival, or adaptive resistance mechanisms. Therefore, the functional impact of S100-A11 may extend beyond the parameters evaluated here and warrants further investigation using complementary experimental approaches.

Our findings should also be interpreted in the context of our previous work identifying CAF-derived FGF5 as a mediator of resistance to anti-HER2 therapy through FGFR2 activation [15]. Together, these studies support a model in which CAFs promote therapeutic resistance not through a single dominant factor, but via a broader adaptive secretory program that sustains tumour cell survival under pharmacological pressure. Although the present data do not allow us to define whether the FGF5/FGFR2 and S100-A11/RAGE/STAT3 axes operate sequentially, hierarchically, or in parallel, the partial reversal of resistance observed upon S100-A11 silencing suggests that S100-A11 is unlikely to be the sole CAF-derived mediator responsible for STAT3 activation. This is consistent with the existence of multiple, potentially cooperative CAF-derived signalling pathways contributing to resistance. We therefore propose that FGF5/FGFR2 and S100-A11/RAGE/STAT3 may represent complementary mechanisms whose relative contribution could vary across tumours and biological contexts. Elucidating whether distinct patient subsets are preferentially driven by one or the other pathway, and whether combined targeting of these stromal axes may improve therapeutic efficacy, will require further investigation.

The blockade of RAGE with azeliragon, a selective antagonist, increased the sensitivity of BT-474 and EFM-192A cells to TPD treatment and significantly reversed the resistance to the combined therapy induced by either exogenous S100-A11 or CM from CAF-200. Previous studies have demonstrated that pharmacological blockade of RAGE inhibits cell proliferation [55,56], and specifically the S100-A11/RAGE/STAT3 axis in cancer cells [23]. Studies in murine models of triple-negative breast cancer (TNBC) have demonstrated the efficacy of inhibiting RAGE, through both pharmacological and genetic methods, in reducing tumour growth *in vivo* [57,58]. Although the effects of azeliragon in cancer models have not been extensively studied, recent research in breast cancer has demonstrated the use of this inhibitor and its derived compounds in TNBC murine models [59,60]. The administration of azeliragon led to a reduction of p-STAT3 levels in TPD-incubated cells exposed to the S100-A11r protein. Conversely, it increased ERK phosphorylation, which may be a compensatory response. Previous studies have demonstrated that RAGE blockade in TNBC models resulted in a reduction in STAT3 phosphorylation, which has been linked to a suppression of metastasis *in vitro* [57,59]. Our findings suggest that RAGE plays a regulatory role in the signalling cascade initiated by extracellular S100-A11, which ultimately affects the STAT3 pathway. Together, these results suggest that the S100-A11/RAGE/STAT3 axis may serve as a mechanism of resistance to TPD therapy, indicating that RAGE could be a viable therapeutic target. The data also indicated that modulating the secretome activity of CAFs may be a potential strategy to attenuate resistance to anti-HER2 therapy in HER2+ breast cancer (Figure S19). Although our data support the involvement of the S100-A11/RAGE/STAT3 axis, the evidence is primarily based on pharmacological inhibition and correlative signalling changes. The absence of robust genetic perturbation models (e.g., RAGE or STAT3 knockdown or knockout) represents a limitation of the study. Therefore, while our findings are consistent with a functional role of this signalling axis, a definitive causal relationship cannot be fully established.

We employed a murine xenograft model to investigate the effects of our proposed therapeutic intervention in HER2+ breast cancer, with specific focus on the role of the TME and CAFs in mediating resistance to anti-HER2 therapies. The initial finding was the validation of the role of S100-A11 as an inducer of resistance to TDP treatment. The diminished efficacy of the therapy observed in the S100-A11r + TPD group underscores the role of S100-A11 in inducing resistance, a finding that aligns with recent research indicating that CAF-derived factors can promote treatment resistance [61]. It is noteworthy that the combination of TPD and azeliragon effectively countered S100-A11–induced resistance. The 24% increase in tumour size caused by the addition of S100-A11r (with respect to the TDP-only group) was counteracted by a reduction of 25% when azeliragon was included in the therapy, corroborating our prior *in vitro* observations and suggesting that azeliragon exerts a specific effect on the resistance induced by S100-A11. RAGE, a multifaceted molecule, interacts with various ligands, including S100 proteins, to activate downstream signalling pathways such as STAT3. Azeliragon exerts its antineoplastic effects by blocking RAGE, thereby effectively disrupting the signalling axis that regulates tumour cell proliferation. Such disruption leads to the reversal of resistance to TPD therapy. This approach is further substantiated by recent literature, which emphasises the potential of targeting the RAGE/S100 axis in cancer therapy [62]. Finally, the S100-A11r and azeliragon groups exhibited a slight, non-significant increase in tumour volume (and significantly less than the control group). The statistical analysis confirmed the significance of these findings, with a highly significant *p* for TPD + azeliragon (in the presence of S100-A11r) vs. TPD (in the presence of S100-A11r) (*p* < 0.01). These results underscore the potential of azeliragon to counteract resistance mechanisms in breast cancer therapy, warranting further molecular and histopathological investigations.

The *in vivo* model used in this study should be interpreted as a reductionist proof-of-principle system rather than as a full representation of CAF–tumour interactions. Administration of recombinant S100-A11 allowed us to isolate the contribution of extracellular S100-A11 to TPD resistance and to evaluate whether pharmacological RAGE inhibition could reverse this effect under controlled conditions. This approach reduces confounding from other CAF-derived factors present in the stromal secretome. However, it does not reproduce the continuous, spatially organized, and context-dependent secretion of S100-A11 by CAFs within the TME. Therefore, these findings support the functional relevance of extracellular S100-A11 *in vivo*, but do not establish that CAF-derived S100-A11 alone is sufficient to drive resistance in a physiological stromal setting. Future studies using orthotopic models or co-implantation of tumour cells with control versus S100A11-silenced CAFs will be required to validate this mechanism in a more representative microenvironmental context.

Emerging evidence indicates that S100-A11 is expressed in breast tumours and may contribute to disease progression by exerting oncogenic effects. Research suggests that S100-A11 may have the potential to serve as a prognostic biomarker of poor outcomes [[63], [64], [65], [66]]. These findings have linked S100-A11 overexpression to drug resistance and to an immunosuppressive TME, which is characterised by the presence of CAFs and lymphocytic infiltration [63]. However, the specific role of S100-A11 from CAFs, as well as its prognostic and predictive value in breast cancer therapy response, remains poorly understood. In a retrospective study, we evaluated the expression of S100-A11 in CAFs from early-stage HER2+ breast carcinoma biopsies. The aim was to assess the clinical relevance of stromal S100-A11 expression in patient outcomes following neoadjuvant TPD therapy. Given the retrospective design and limited sample size, these findings should be interpreted as exploratory. The analysis revealed that S100-A11 is expressed in the stromal component, where it was found in both the cytoplasm and nucleus of CAFs. Additionally, S100-A11 expression was observed in tumour cells, predominantly in the cytoplasm but also in the nucleus, consistent with previous studies [64,66]. Furthermore, p-STAT3 expression was predominantly localised in the nucleus of neoplastic cells, as previously reported in HER2+ breast tumours [67].

A key issue concerns the cellular origin of S100-A11 within tumour tissues, given its expression across multiple compartments, including tumour cells, immune infiltrates, and endothelial cells. To address this, we performed dual staining for S100-A11 and α-SMA, a well-established CAF marker, enabling spatial identification of stromal fibroblasts expressing S100-A11. This analysis demonstrated clear co-localisation in a subset of cases, providing direct evidence that CAFs represent a source of S100-A11 in human HER2+ breast tumours. At the same time, S100-A11 expression was not restricted to CAFs, highlighting the complexity of its distribution within the TME. Rather than undermining our model, this supports a context-dependent role in which stromal S100-A11 contributes to paracrine signalling, while tumour cell–intrinsic expression may represent an additional, potentially independent component. In line with this, our clinical analyses were restricted to cases in which S100-A11 expression could be confidently assigned to the stromal compartment based on morphological criteria, thereby minimising confounding from tumour cell–derived signal. These findings support the presence of a stromal S100-A11 component that is both biologically and histologically identifiable and is consistent with the functional effects observed in our experimental models. However, despite the use of dual staining and compartment-specific scoring, the possibility of partial overlap or ambiguity in signal attribution cannot be completely excluded. Therefore, while our data support a stromal contribution to S100-A11-mediated effects, they do not imply that CAFs are the exclusive source of this protein within the TME.

In our study, S100-A11 expression in CAFs was found to significantly correlate with tumour pathological response. This was evidenced by a significant decrease in tumours with S100-A11 high expression in CAFs achieving Miller and Payne's G5 response (Table 2). Although the correlation with axillary response was not statistically significant, tumours with high S100-A11 expression in CAFs exhibited higher post-treatment presence of axillary tumour cells compared with cases with low S100-A11 expression, which showed no axillary response. In the context of therapy resistance, the majority of HER2+ breast tumours with high S100-A11 expression in CAFs exhibited residual disease following anti-HER2 therapy, while only a minority achieved pCR. The observed reduction in pathological response in the high S100-A11 expression group was consistent with our findings in BCCLs, suggesting that S100-A11 from CAFs may play a key role in tumour response to anti-HER2 TPD therapy. Furthermore, our study demonstrated a positive correlation between the elevated expression of S100-A11 in CAFs and high levels of p-STAT3 in tumour cells, consistent with a stromal-mediated paracrine mechanism. Importantly, the combined analysis of stromal S100-A11 and tumour p-STAT3 identified a co-high subgroup with a markedly lower probability of achieving pCR and a higher frequency of residual nodal disease. This suggests that integrating stromal and tumour-cell biomarkers may better capture therapy-resistant TME interactions than either marker alone. Although these findings require validation in independent cohorts, they support the clinical relevance of the proposed stromal S100-A11–STAT3 signalling axis.

The use of dual anti-HER2 blockade has demonstrated substantial clinical benefits in early-stage HER2+ breast cancer, culminating in an enhanced 5-year recurrence-free survival rate [4]. Consequently, the cohort exhibited a low incidence of disease recurrence and deaths, which has limited the power of our analysis of the potential relationship between stromal S100-A11 protein expression and disease outcomes. However, given that patients with residual disease after treatment have a significantly higher risk of recurrence [[68], [69], [70]], achieving pCR is a surrogate endpoint for long-term survival without recurrence.

The interpretation of the clinical data should consider several methodological limitations. Cut-off values for S100-A11 and p-STAT3 expression were derived using ROC curve analysis within the same cohort based on pathological response, which may introduce overfitting and limit the generalizability of these thresholds. Although multivariable regression analysis was performed, the clinical findings should still be interpreted with caution because of the retrospective single-centre design, limited sample size, low number of recurrence events, and lack of external validation. In addition, cut-off values were derived using ROC analysis within the same cohort, which may introduce overfitting and limit the generalizability of these thresholds. Given the number of comparisons performed, the possibility of type I error cannot be excluded. Therefore, the independent predictive value of stromal S100-A11 cannot be established from the present data, and these findings should be considered hypothesis-generating. Within these limitations, our clinical results support an association between high S100-A11 expression in CAFs and the presence of residual disease after neoadjuvant anti-HER2 therapy. Although elevated stromal S100-A11 may identify tumours with reduced sensitivity to treatment, its relationship with recurrence risk could not be formally assessed in this cohort because of the low number of events. Therefore, validation in larger, independent cohorts with longer follow-up and multivariable modelling will be required to determine the clinical utility of stromal S100-A11 as a biomarker of therapy response. Overall, these findings suggest that high S100-A11 expression in CAFs is associated with a lower probability of achieving pCR following neoadjuvant anti-HER2 treatment. If confirmed, stromal S100-A11 could help identify patients with reduced sensitivity to standard neoadjuvant therapy and may provide a rationale for future studies exploring therapeutic strategies aimed at disrupting CAF-derived S100-A11 signalling.

This study has certain limitations worth noting. The use of a limited number of cell lines may restrict the generalizability of the findings. Additionally, further investigation is warranted to elucidate the role of RAGE interactions with ligands other than S100-A11, particularly in the context of RAGE inhibition, to understand their contributions in both cell and *in vivo* models. Although findings from clinical samples supported our *in vitro* observations, the retrospective, single-centre design and relatively small sample size may limit the statistical power and broader clinical applicability of the results. Moreover, variability in patient follow-up durations could affect the assessment of long-term outcomes such as recurrence.

## Conclusions

In this study, we identify S100-A11 as a stromal-derived factor associated with reduced sensitivity to anti-HER2 TPD therapy in HER2+ breast cancer models. Functional experiments indicate that extracellular S100-A11 is linked to increased tumour cell proliferation and STAT3 activation, while silencing of S100A11 in CAFs attenuates the resistance-promoting effects of conditioned medium. Pharmacological inhibition of STAT3 or RAGE partially reverses this phenotype, supporting the involvement of the S100-A11/RAGE/STAT3 signalling axis under defined experimental conditions.

In the clinical setting, stromal S100-A11 expression was associated with tumour p-STAT3 levels and reduced pathological response to neoadjuvant anti-HER2 therapy, reflected by a lower probability of achieving pCR and increased residual disease, although these findings should be considered exploratory. While S100-A11 is not exclusively CAF-derived, its stromal expression appears to be functionally relevant within the tumour microenvironment.

Overall, these results support a role for stromal S100-A11 in modulating response to anti-HER2 therapy and provide a rationale for further investigation of the S100-A11/RAGE/STAT3 axis as a potential target in stromal-mediated resistance. Validation in larger clinical cohorts and more physiologically representative models will be required to determine its translational relevance.

## Funding

The present work was funded by Instituto de Salud Carlos III (ISCIII) through the projects PI21/00002, PI21/00142, PI24/00160 and PI24/00192, and co-funded by the European Union; CIBERONC (Biomedical Research Networking Centre for Cancer, CB16/12/00481); Generalitat de Catalunya (2021 SGR 00776). M.M.-G. was supported by a Predoctoral Health Research Training contract from the ISCIII (FI22/00324). M.S.-A. was supported by a Jiménez Díaz predoctoral research grant funded by the Fundación Conchita Rábago de Jiménez Díaz. C.A. was supported by a Research Assistant grant from the Comunidad de Madrid (PEJ-2023-AI/SAL-GL-27249).

## Institutional Review Board statement

Not applicable.

## Consent for publication statement

Not applicable.

## Data availability

Not applicable.

## CRediT authorship contribution statement

**Melani Luque:** Writing – original draft, Methodology, Investigation, Formal analysis. **Miriam Morales-Gallego:** Methodology, Investigation. **Marta Sanz-Álvarez:** Writing – review & editing, Methodology, Investigation. **Claudia Arguiñano:** Methodology, Investigation. **Yamileth Rangel:** Resources, Data curation. **Natalia Ramírez-Merino:** Resources, Data curation. **Mengjuan Qin:** Writing – review & editing, Methodology, Investigation. **Ana Rovira:** Writing – review & editing, Validation. **Joan Albanell:** Writing – review & editing, Funding acquisition. **Juan Madoz-Gúrpide:** Writing – review & editing, Writing – original draft, Supervision, Formal analysis, Conceptualization. **Federico Rojo:** Writing – review & editing, Supervision, Funding acquisition, Conceptualization.

## Declaration of competing interest

The authors declare the following financial interests/personal relationships which may be considered as potential competing interests:

Federico Rojo reports article publishing charges and equipment, drugs, or supplies were provided by Carlos III Health Institute. Joan Albanell reports article publishing charges and equipment, drugs, or supplies were provided by Government of Catalonia. Joan Albanell reports travel was provided by Biomedical Research Network Centre in Cancer. If there are other authors, they declare that they have no known competing financial interests or personal relationships that could have appeared to influence the work reported in this paper.

## Appendix Supplementary materials

Image, application 1

Image, application 2

Image, application 3

Image, application 4
