# Supplementary material for: Cancer-associated fibroblast-derived protein S100-A11 influences the response to anti-HER2 therapies in HER2-positive breast cancer
Source: Neoplasia. 2026 May 19;78:101318. doi: 10.1016/j.neo.2026.101318 (PMC13213234; doi:10.1016/j.neo.2026.101318)
Supplement: Supplementary file 1 [file mmc1.docx]

# SUPPLEMENTARY MATERIALS

**Document S1.** Donor information document for biological sample donations to the Fundación Jiménez Díaz Biobank (supplementary file).

**Document S2.** Certificate of approval for the research project involving the collection of samples from patients included in this study. This approval has been granted by the Clinical Trials Committee of the Fundación Jiménez Díaz University Hospital, Madrid, Spain (supplementary file).


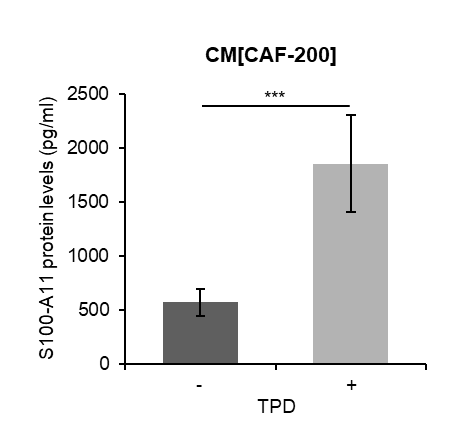


**Figure S1.** The validation of S100-A11 protein levels (in pg/ml) in CAF-200–derived CM following treatment with TPD (15 μg/ml T; 20 μg/ml P; 0.5 nM D) was determined by ELISA. CM[CAF-200]: CAF-200–CM. (***): *p* < 0.001. The error bars represent the calculated value of the standard deviation (n = 3).


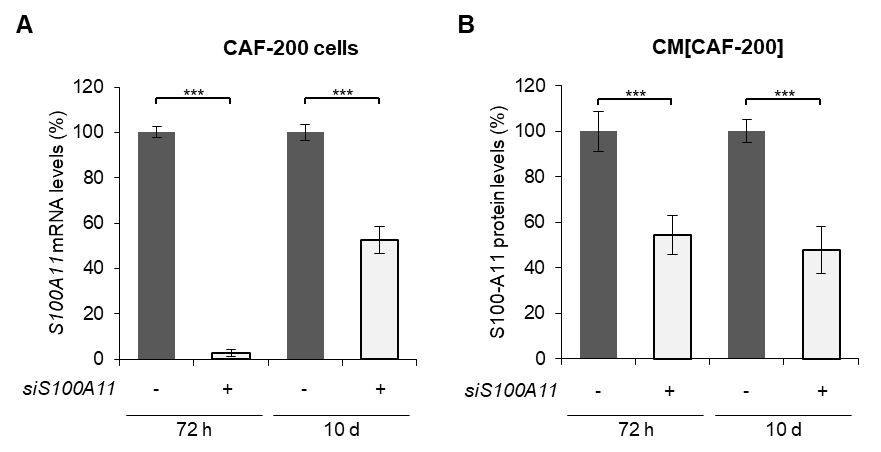


Figure S2. **A.** Following *S100A11* gene silencing in CAF-200 fibroblasts, the relative levels of S100A11 mRNA were determined by quantitative PCR (qRT-PCR) at 72 h or 10 days. **B.** Subsequently, the relative levels of S100-A11 protein were determined in the CAF-200–CM by enzyme-linked immunosorbent assay (ELISA) under the indicated conditions. CM[CAF-200]: CAF-200–CM. siS100A11: *S100A11* gene silencing. (***): *p* < 0.001. Error bars represent the calculated value of the standard deviation (n = 4).


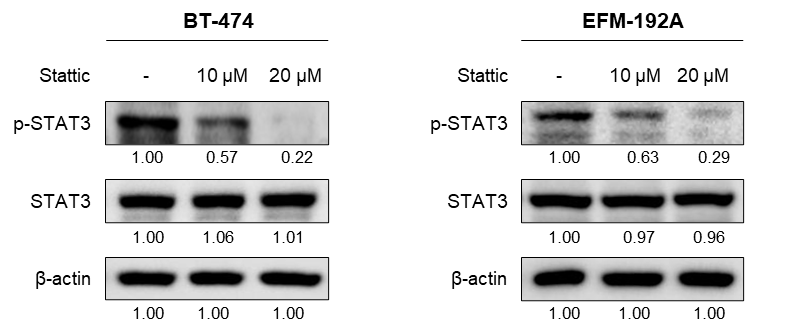


Figure S3. WB analyses of p-STAT3 and STAT3 on BT-474 and EFM-192A cells, respectively, after treatment for 6 h with 10 or 20 μM stattic. Relative abundance levels of proteins were determined by densitometric analysis of the images, normalising them with the β-actin loading control and with the respective untreated control. Representative images are shown for n = 2.


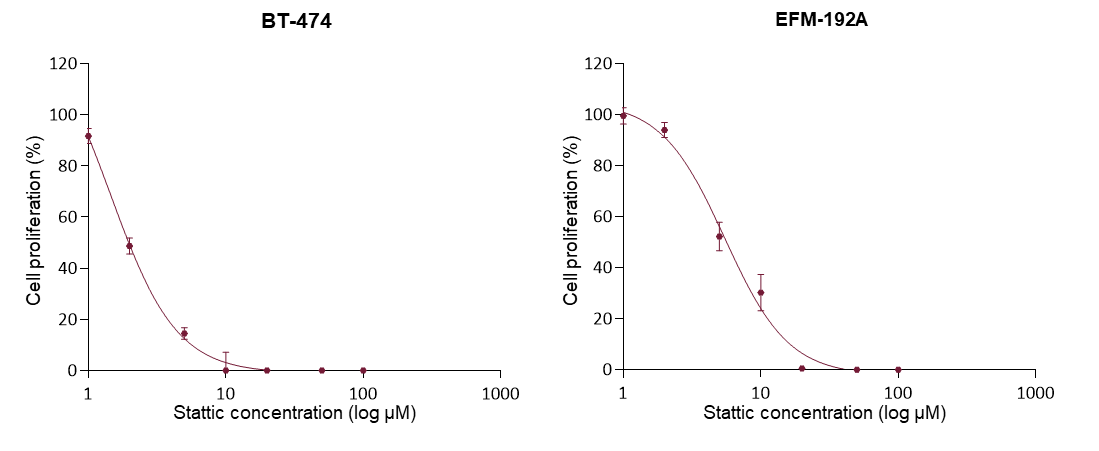


Figure S4. Proliferation rates of BT-474 and EFM-192A cells, respectively, after treatment with increasing concentrations of stattic ranging from 1 to 100 μM, represented on a logarithmic scale on the X-axis. The continuous line represents the curve of the nonlinear fitting model. Error bars represent the calculated value of the standard deviation (n=3).

Figure S5. Partial validation of the effect of STAT3 inhibition on S100-A11r–mediated resistance in AU-565 cells. The figure shows the proliferation rate of AU-565 cells after treatment with stattic in the presence of exogenous S100-A11r and TPD therapy. Stattic reduced S100-A11r–associated proliferation under TPD treatment, supporting the involvement of STAT3 in S100-A11–mediated resistance in an additional HER2+ breast cancer cell model. S100-A11r: recombinant S100-A11 protein. Statistical significance is indicated as follows: (*) *p* < 0.05; (**) *p* < 0.01. Error bars represent SD.


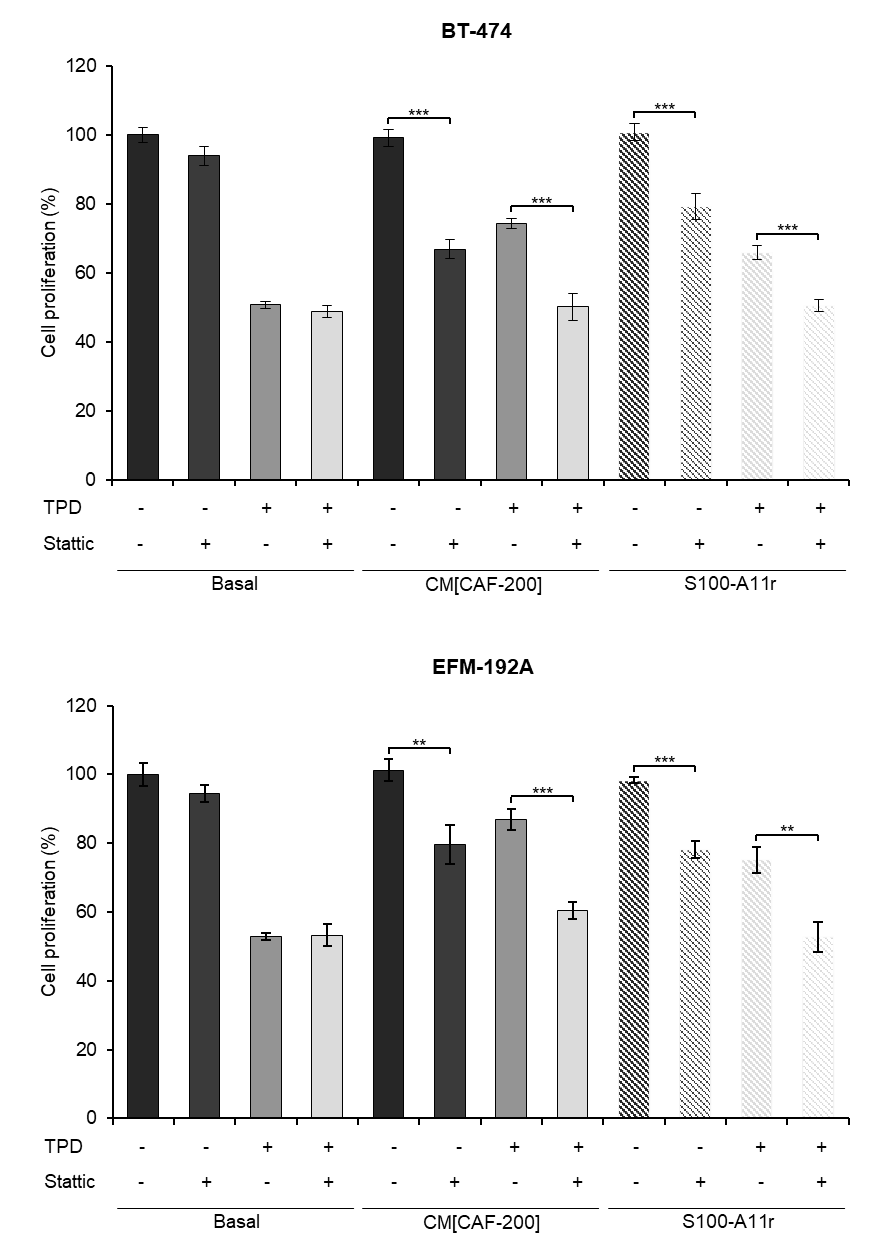


Figure S6. Treatment with stattic reversed BCCL sensitivity to the basal levels of the TPD therapy, despite the addition of the CAF-200–CM. The figure shows the proliferation rates of BT-474 and EFM-192A cells after treatment with stattic, exposed to either CAF-200–CM or exogenous S100-A11r (20 ng/ml) for 5 days, with TPD therapy. Other conditions as in previous figures. Shaded bar graphs correspond to data previously presented in Figure 3 (S100-A11r conditions) and are reproduced here for comparative purposes only, to enable direct evaluation against CM[CAF-200] effects. These panels do not represent independent experiments.


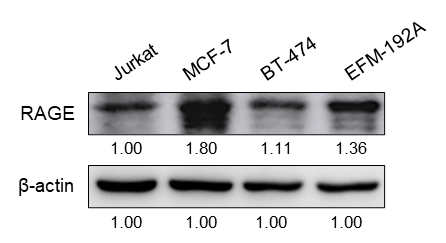


Figure S7. A WB analysis was conducted on RAGE in BT-474 and EFM-192 lysates, with lysates from Jurkat and MCF-7 cells serving as positive controls. The relative abundance of protein levels was determined by densitometric analysis of the images, normalised to β-actin loading controls and to Jurkat levels. Representative images are shown for n = 2.


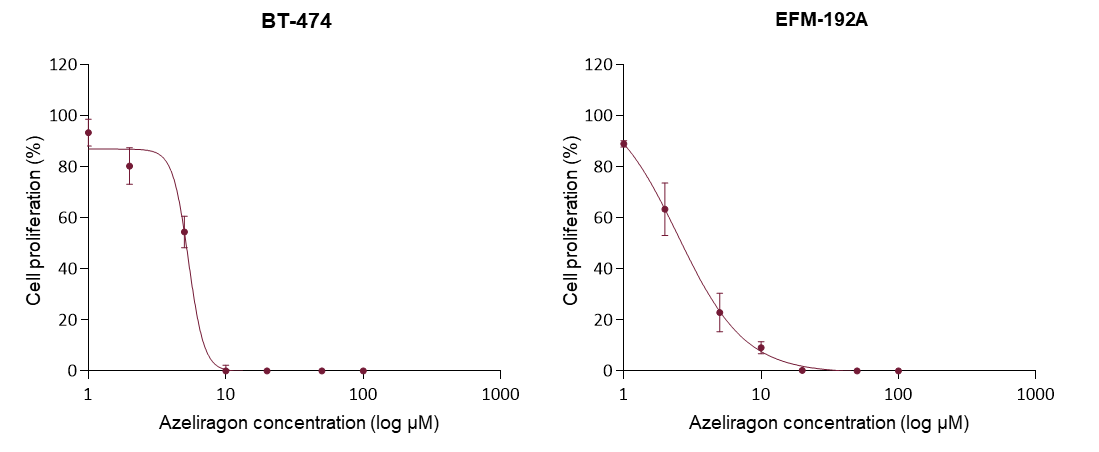


Figure S8. Proliferation rates of BT-474 and EFM-192A cells, respectively, following treatment with increasing concentrations of azeliragon ranging from 1 to 100 μM. The data is represented on a logarithmic scale on the X-axis. The continuous line represents the curve of the nonlinear fitting model. Error bars represent the calculated value of the standard deviation (n = 3).

Figure S9. Proliferation rates of BT-474 and EFM-192A cells after treatment with 1 μM azeliragon, exposed to CAF-200–CM or exogenous S100-A11r, for 5 days, with or without TPD therapy. Conditions as in previous figures. Error bars represent the standard deviation (n=4). Shaded bar graphs correspond to data previously presented in Figure 4 (S100-A11r conditions) and are reproduced here for comparative purposes only, to enable direct evaluation against CM[CAF-200] effects. These panels do not represent independent experiments.


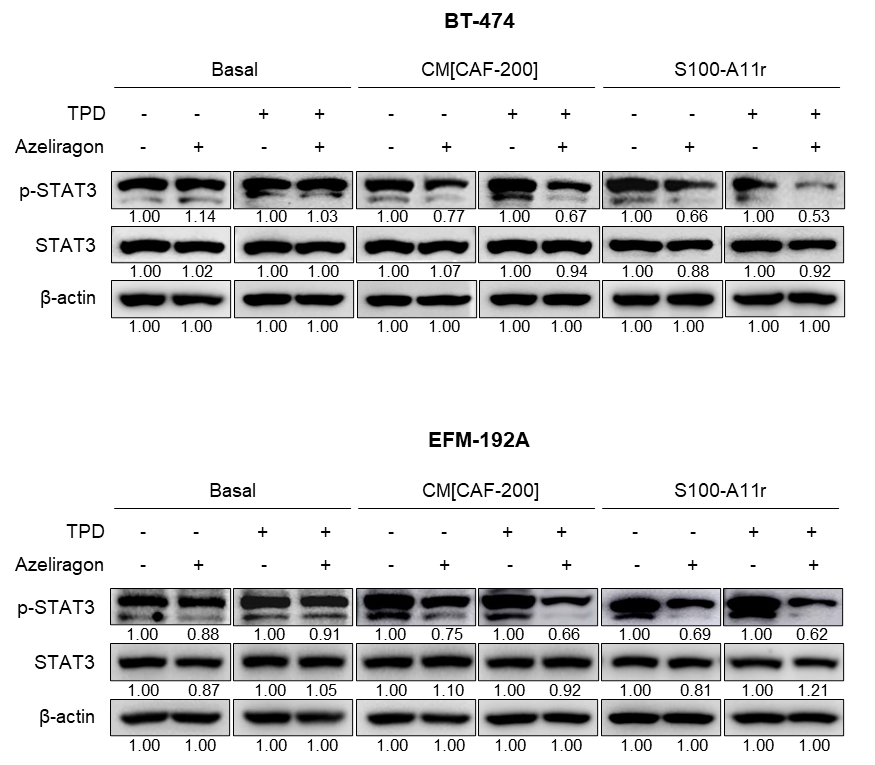


Figure S10. WB analyses of p-STAT3 and STAT3 on BT-474 and EFM-192A cells. The effect of treatment with 20 μM azeliragon for 6 h was assessed on tumour cells exposed to CM from CAF-200 or exogenous S100-A11r. Conditions as in previous figures. Images are shown representative for n = 3.


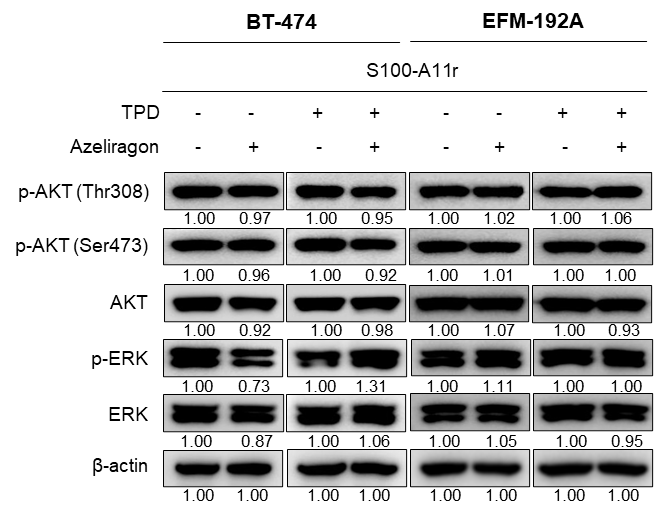


Figure S11. WB analysis on BT-474 and EFM-192A cells of phosphorylated and total forms of AKT and ERK. The effect of treatment with 20 μM azeliragon for 6 h was assessed on tumour cells exposed to exogenous S100-A11r, with or without TPD therapy. The images presented herein are intended to serve as illustrative representations, with n = 3.

Figure S12. Longitudinal monitoring of body weight in the in vivo study. Body weight of mice was recorded throughout the experimental period across all treatment groups, including control, TPD, S100-A11r, azeliragon, and their respective combinations. No significant differences in body weight were observed between groups, and no sustained weight loss was detected, indicating that the treatments were well tolerated under the experimental conditions. Data are presented as mean.


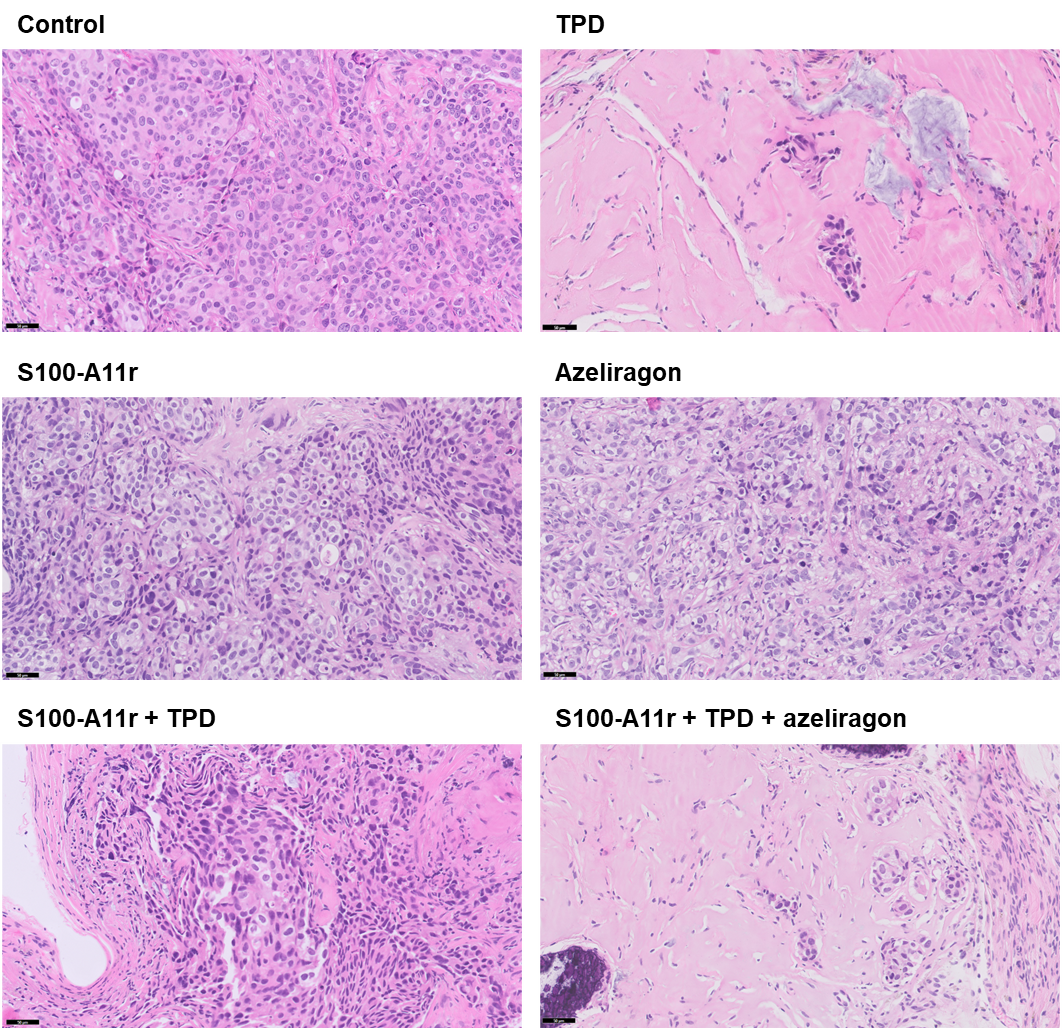


Figure S13. Representative images of haematoxylin and eosin staining from the different treatment groups. Images were captured at ×200 magnification. Scale bar: 50 µm.


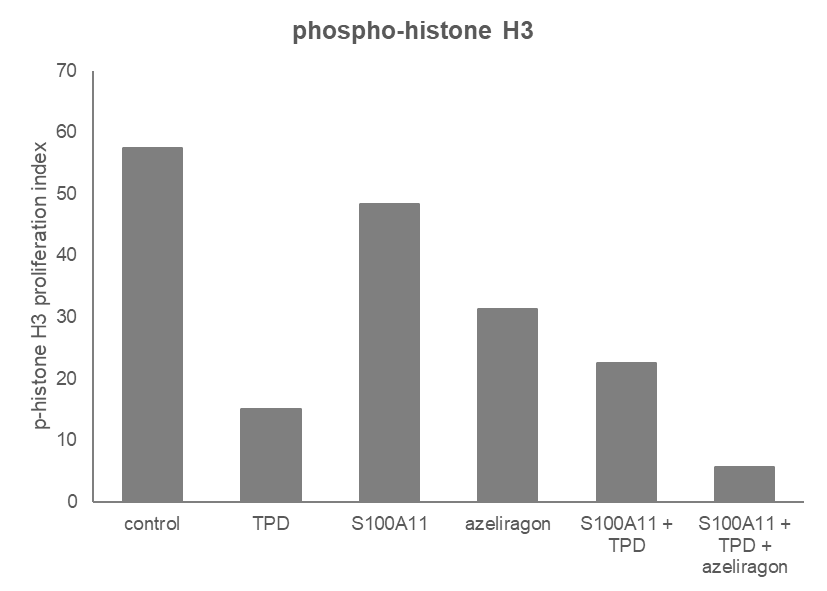


Figure S14. The combination of TPD with azeliragon significantly reduced proliferation of S100-A11–induced TPD-resistant xenograft human tumours in murine models. Bar chart presents the IHC detection of p-H3 expression in tumour samples from the different groups of treatment in the murine model. All results are expressed as mean ± s.e.m. values (n = 5).

|  | **No. cases** | | **S100-A11 Low-level** | | **S100-A11 High-level** | |  | **p-STAT3 Low-level** | | **p-STAT3 High-level** | |  |
| --- | --- | --- | --- | --- | --- | --- | --- | --- | --- | --- | --- | --- |
|  | **(n = 77)** | **%** | **(n = 43; 55.8%)** | | **(n = 34; 44.2%)** | | **χ2 p** | **(n = 21; 27.3%)** | | **(n = 56; 72.7%)** | | **χ2 p** |
|  |  |  | **No. cases** | **%** | **No. cases** | **%** |  | **No. cases** | **%** | **No. cases** | **%** |  |
| **Age (years) (median. range)** | 53 (28-87) |  |  |  |  |  |  |  |  |  |  |  |
| **Hormonal status** |  |  |  |  |  |  |  |  |  |  |  |  |
| **Premenopausal** | 29 | 37.7 | 16 | 37.2 | 13 | 38.2 | 0.926 | 7 | 33.3 | 22 | 39.3 | 0.631 |
| **Postmenopausal** | 48 | 62.3 | 27 | 62.8 | 21 | 61.8 |  | 14 | 66.7 | 34 | 60.7 |  |
| **Family history** |  |  |  |  |  |  |  |  |  |  |  |  |
| **No** | 50 | 64.9 | 26 | 60.5 | 24 | 70.6 | 0.355 | 12 | 57.1 | 38 | 67.9 | 0.380 |
| **Yes** | 27 | 35.1 | 17 | 39.5 | 10 | 29.4 |  | 9 | 42.9 | 18 | 32.1 |  |
| **Morphologic tye** |  |  |  |  |  |  |  |  |  |  |  |  |
| **NOS** | 69 | 89.6 | 41 | 95.3 | 28 | 82.4 | 0.097 | 20 | 95.2 | 49 | 85.7 | 0.507 |
| **CLI** | 5 | 6.5 | 2 | 4.7 | 3 | 8.8 |  | 1 | 4.8 | 4 | 7.1 |  |
| **Other** | 3 | 3.9 | 0 | 0 | 3 | 8.8 |  | 0 | 0 | 3 | 5.4 |  |
| **Histological grading** |  |  |  |  |  |  |  |  |  |  |  |  |
| **(Scarff-Bloom-Richardson)** |  |  |  |  |  |  |  |  |  |  |  |  |
| **II** | 23 | 29.9 | 14 | 32.6 | 9 | 26.5 | 0.562 | 4 | 19.0 | 19 | 33.9 | 0.204 |
| **III** | 54 | 70.1 | 29 | 67.4 | 25 | 73.5 |  | 17 | 81.0 | 37 | 66.1 |  |
| **ER status** |  |  |  |  |  |  |  |  |  |  |  |  |
| **Negative** | 27 | 35.1 | 19 | 44.2 | 8 | 23.5 | 0.059 | 11 | 52.4 | 16 | 28.6 | 0.051 |
| **Positive** | 50 | 64.9 | 24 | 55.8 | 26 | 76.5 |  | 10 | 47.6 | 40 | 71.4 |  |
| **PR status** |  |  |  |  |  |  |  |  |  |  |  |  |
| **Negative** | 46 | 59.7 | 31 | 72.1 | 15 | 44.1 | 0.013 | 15 | 71.4 | 31 | 55.4 | 0.200 |
| **Positive** | 31 | 40.3 | 12 | 27.9 | 19 | 55.9 |  | 6 | 28.6 | 25 | 44.6 |  |
| **Ki-67** |  |  |  |  |  |  |  |  |  |  |  |  |
| **< 20%** | 10 | 13.0 | 4 | 9.3 | 6 | 17.6 | 0.358 | 3 | 14.3 | 7 | 12.5 | 0.232 |
| **≥ 20%** | 67 | 87.0 | 39 | 90.7 | 28 | 82.4 |  | 18 | 85.7 | 49 | 87.5 |  |
| **T** |  |  |  |  |  |  |  |  |  |  |  |  |
| **1** | 2 | 2.6 | 1 | 2.3 | 1 | 2.9 | 0.568 | 0 | 0 | 2 | 3.6 | 0.199 |
| **2** | 56 | 72.7 | 30 | 69.8 | 26 | 76.5 |  | 14 | 66.7 | 42 | 75.0 |  |
| **3** | 12 | 15.6 | 6 | 14.0 | 6 | 17.6 |  | 3 | 14.3 | 9 | 16.1 |  |
| **4** | 6 | 7.8 | 5 | 11.6 | 1 | 2.9 |  | 4 | 19.0 | 2 | 3.6 |  |
| **Unknown** | 1 | 1.3 | 1 | 2.3 | 0 | 0 |  | 0 | 0 | 1 | 1.8 |  |
| **N** |  |  |  |  |  |  |  |  |  |  |  |  |
| **0** | 38 | 49.4 | 20 | 46.5 | 18 | 52.9 | 0.492 | 10 | 47.6 | 28 | 50.0 | 0.830 |
| **1** | 37 | 48.1 | 22 | 51.2 | 15 | 44.1 |  | 11 | 52.4 | 26 | 46.4 |  |
| **2** | 1 | 1.3 | 1 | 2.3 | 0 | 0 |  | 0 | 0 | 1 | 1.8 |  |
| **3** | 1 | 1.3 | 0 | 0 | 1 | 2.9 |  | 0 | 0 | 1 | 1.8 |  |

Table S1. The clinical-pathological characteristics of early HER2+ breast cancer patients treated with neoadjuvant anti-HER2 therapy plus chemotherapy, and the distribution of cases based on the expression of S100-A11 in CAFs and p-STAT3 in tumour cells in relation to the clinical-pathological indicators are presented. The distribution of the number of cases and percentages for each category is shown. Carcinoma NOS: infiltrating ductal carcinoma, not otherwise specified. CLI: infiltrating lobular carcinoma. ER: oestrogen receptor. PR: progesterone receptor. T and N correspond to tumour and node categories, respectively, of the TNM staging system.


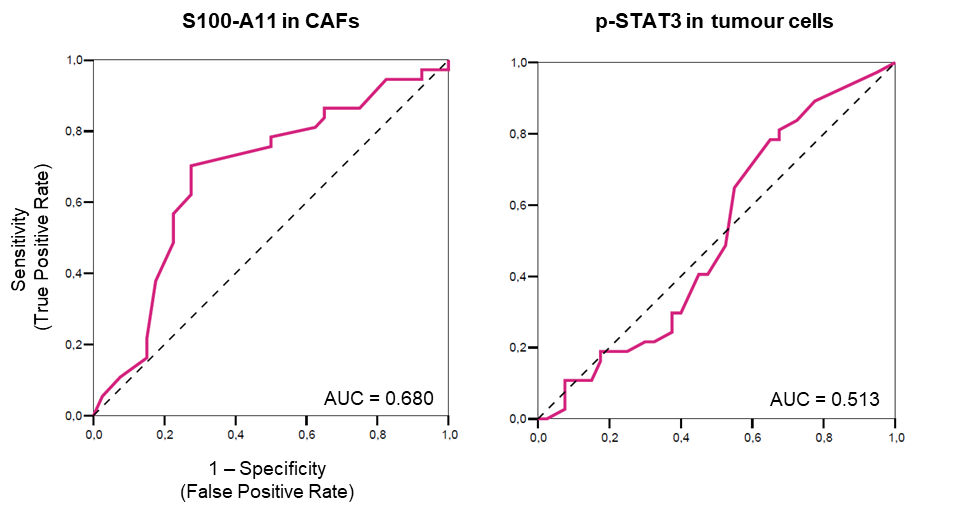


Figure S15. ROC curve representation for determining the cut-off point defining high expressions of S100-A11 in CAFs and p-STAT3 in tumour cells, respectively. According to the ROC curve analysis, based on the identification of cases with residual disease after neoadjuvant treatment, the H-score value established as a cut-off point was ≥ 82 (with 75.7% sensitivity and 72.5% specificity) for S100-A11 high expression, and ≥ 11 (83.8% sensitivity and 32.5% specificity) for p-STAT3 high expression.


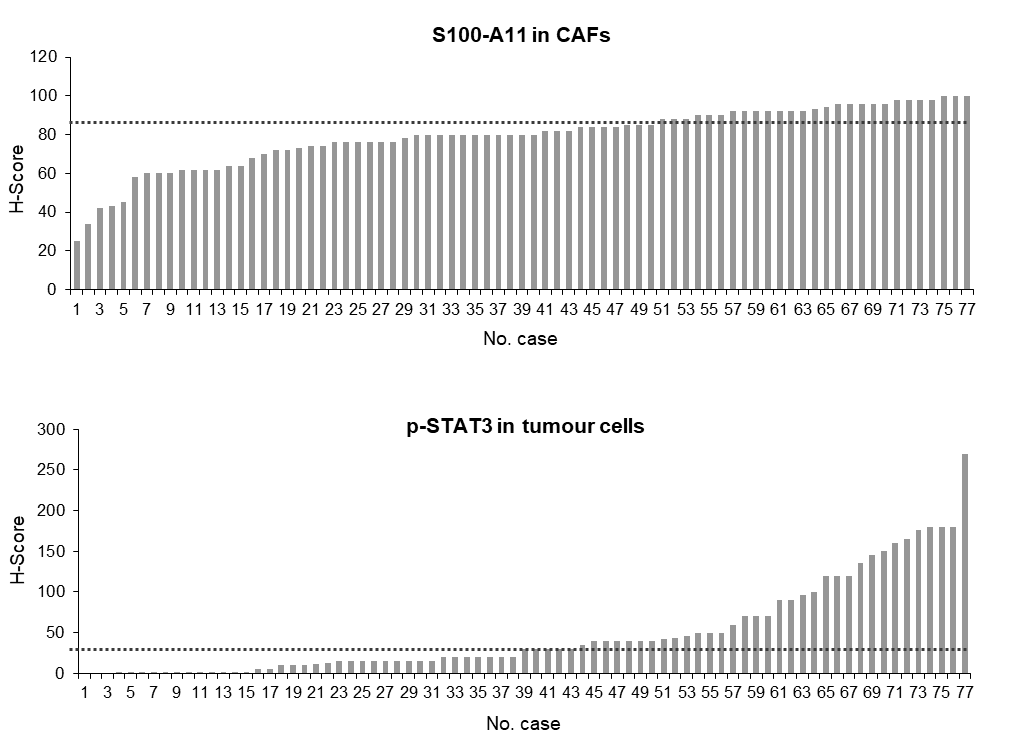


Figure S16. The distribution of patient cases with early HER2+ breast cancer is represented here based on the H-score determined for S100-A11 in CAFs and for p-STAT3 in tumour cells, respectively. The cut-off point, represented by the dotted line, was set at ≥ 82 for S100-A11 and ≥ 11 for p-STAT3. (It is important to note that the case number (#1 to #77) is representative and does not correspond to the same case across the two panels.)

Figure S17. Association between S100-A11 expression in CAFs and tumour p-STAT3 levels in HER2+ breast cancer. **A.** Distribution of p-STAT3 expression in tumour cells according to S100-A11 levels in CAFs, classified as low or high based on ROC-derived H-score thresholds. **B.** The combined expression pattern of both markers was further used to stratify cases into two groups: tumours with concomitant high S100-A11 expression in CAFs and high p-STAT3 expression in tumour cells (co-high group), and tumours lacking simultaneous high expression. This classification identifies a subset of tumours with coordinated CAF–tumour signalling activity. (*): *p* < 0.05; (***): *p* < 0.001.

|  | **S100-A11 expression in CAFs** | | | |  | **p-STAT3 expression in tumour cells** | | | |  |
| --- | --- | --- | --- | --- | --- | --- | --- | --- | --- | --- |
|  | **Low-level** |  | **High-level** |  |  | **Low-level** |  | **High-level** |  |  |
|  | **No. cases** | **%** | **No. cases** | **%** | **χ2 p** | **No. cases** | **%** | **No. cases** | **%** | **χ2 p** |
|  | **(n = 43)** |  | **(n = 34)** |  |  | **(n = 21)** |  | **(n = 56)** |  |  |
| **Relapse** |  |  |  |  |  |  |  |  |  |  |
| **No** | 39 | 90.7 | 31 | 91.2 | 0.942 | 18 | 85.7 | 52 | 92.9 | 0.332 |
| **Yes** | 4 | 9.3 | 3 | 8.8 |  | 3 | 14.3 | 4 | 7.1 |  |

Table S2. Correlation between S100-A11 expression in CAFs and p-STAT3 in tumour cells with respect to tumour relapse. The distribution of the number of cases and percentages for each category is shown below.


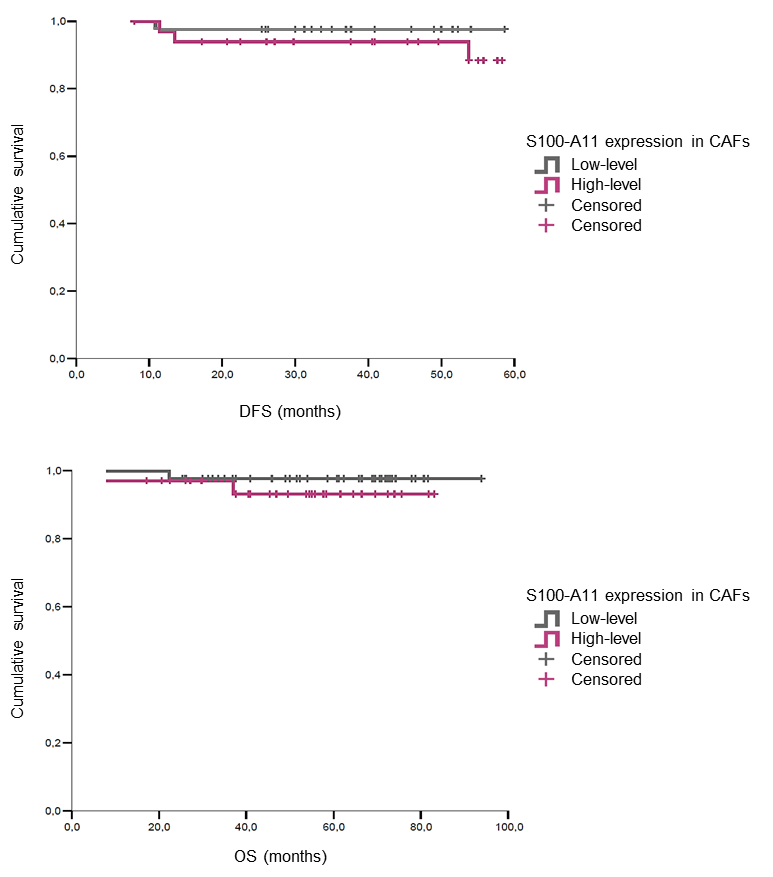


Figure S18. A Kaplan-Meier analysis was employed to assess disease-free survival (DFS) and overall survival (OS), respectively, in a cohort of 77 patients diagnosed with early HER2+ breast cancer who were treated with neoadjuvant anti-HER2 therapy in combination with chemotherapy.

| variable | ***p*-value** | **Hazard Ratio (HR)** | **95% CI (lower – upper)** |
| --- | --- | --- | --- |
| **ER status** | 0.137 | 1.92 | 0.81 – 4.54 |
| **Stromal S100-A11** | 0.001 | 3.61 | 1.71 – 7.62 |
| **Histological grade** | 0.854 | 1.08 | 0.49 – 2.40 |
| **TNM stage** | 0.445 | 1.31 | 0.66 – 2.61 |

Table S3. Multivariable regression analysis of factors associated with residual disease following neoadjuvant anti-HER2 therapy. Hazard ratios (HRs), 95% confidence intervals (CIs), and corresponding *p*-values are shown for each variable included in the model. The analysis included ER status, stromal S100-A11 expression, histological grade, and TNM stage. HR >1 indicates an increased likelihood of residual disease after neoadjuvant anti-HER2 therapy. *p* < 0.05 were considered statistically significant.


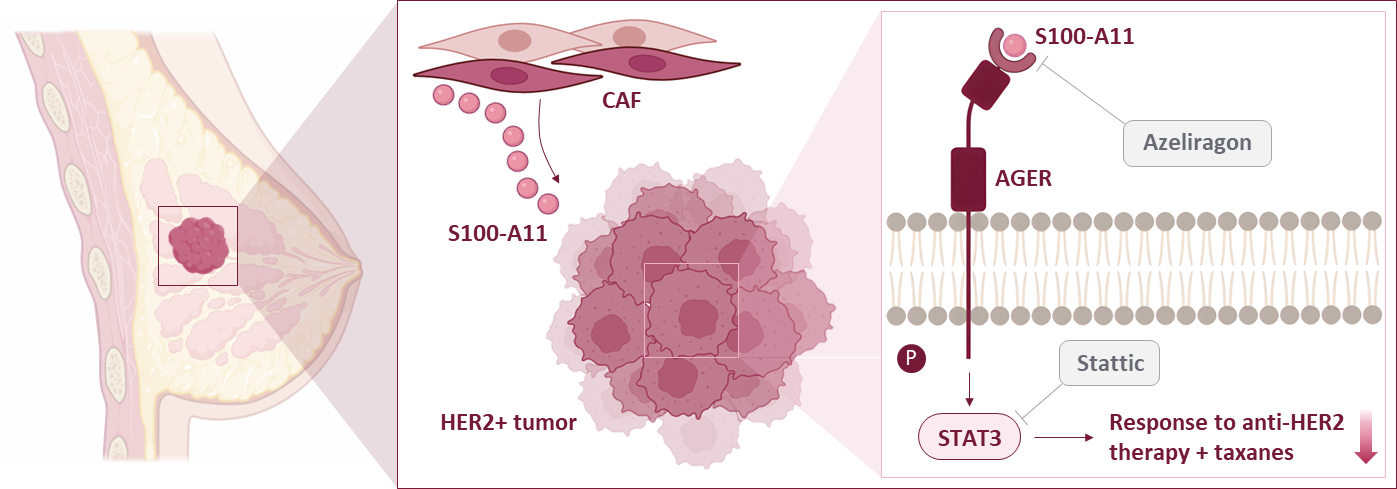


Figure S19. Proposal on the clinical significance of S100-A11 expression in CAFs in response to neoadjuvant anti-HER2 treatment in early-stage HER2+ breast tumours.

Figure S20. Full uncropped gels and blots images (supplementary file).
