# Supplementary material for: Cancer-associated fibroblast-derived protein S100-A11 influences the response to anti-HER2 therapies in HER2-positive breast cancer
Source: Neoplasia. 2026 May 19;78:101318. doi: 10.1016/j.neo.2026.101318 (PMC13213234; doi:10.1016/j.neo.2026.101318)
Supplement: Supplementary file 2 [file mmc2.pdf]

DONOR INFORMATION DOCUMENT FOR BIOLOGICAL SAMPLE DONATIONS TO THE FUNDACIÓN JIMÉNEZ DÍAZ BIOBANK

We request your permission to include in the FJD Biobank certain surplus biological material remaining after the tests being performed on you at the present time in this medical facility or may be performed at a later date, so that these samples may be used for purposes of biomedical research.

Pursuant to the provisions of Law 14/2007 on Biomedical Research, Spanish Royal Decree 1716/2011 regulating Biobanks, Regulation (EU) 2016/679 of General Data Protection of 27 april 2016, Organic Law 3/2018 on the Protection of Personal Data, and the regulations governing their implementation, we ask that you carefully read this document and the attached informed consent form, and sign the document if you agree to take part.

What is a biobank? *An institution for the advancement of research and health*

A biobank is a government-regulated institution that supports biomedical research, which is to say, research aimed at promoting people's health. The biobank acts as the steward of biological samples in adherence of the quality standards and safety guarantees established by law as well as the codes of conduct approved by the pertinent ethics committees. Samples included in a biobank may be transferred to third parties for the purposes medical research, and at all times such transfer takes place under the supervision of a scientific committee and an ethics committee. Samples may be transferred alongside personal information, although prior to any such transfer all personal information is encoded in order to protect the donor's identity.

Biological samples and associated information: *in no case will any testing be performed beyond that which is strictly necessary to provide you with proper medical care*

Excess biological material removed during your care process (i.e., blood samples, bodily fluids, and/or tissues) will be saved and made available for research without causing you any additional discomfort. Donating surplus samples obtained during your care will not prevent you or your family from using them should they be required for health reasons, provided these samples are available. Samples and information related to such samples will remain under the stewardship of and/or be stored in the Fundación Jiménez Díaz Biobank (FJDB) until they have become totally depleted. This biobank is a non-profit organization fully recognized under the National Biobank Registry, which exists under the aegis of the Carlos III Health Institute. The identification number of the FJDB is B.0000647.

Data protection and confidentiality: *samples are stored after being coded*

At all times, personal data will be collected, processed, and stored in such a way as to safeguard confidentiality to the extent provided for in current legislation regarding the protection of personal data. Biological samples forming part of the biobank will be encoded to protect donor identity. Each sample is assigned an identification code, and this code will be used by the researchers. Only personnel authorized by the biobank will be able to reveal your identity by using these codes. Your samples and the related clinical information will be entered into a file belonging to the biobank. This file has been registered with the Spanish Agency for Data Protection. Stewardship will be under the responsibility of the director of the biobank.

Destination and sample transferral to research projects:

By signing the consent form, you give authorization for your samples to be transferred for the purposes of biomedical research projects. You may also state any exceptions to this consent, indicating any and all research aims and methods for which you do not authorize the transfer of your samples. You are guaranteed that any transferral of your samples and associated information

to research projects shall be previously evaluated and approved by the FJD Ethics Committee and the FJDB Scientific Committee and in adherence of ethical and legal requirements. In this case, the FJDB shall only transfer the sample and associated data, including relevant clinical information, in coded form.

Benefits

Donation of samples to the FJDB is done on a voluntary and altruistic basis and at no cost to you. The samples collected may not used in ways directly related to for-profit undertakings. You will not receive any direct benefit stemming from the research performed with the sample material, nor will you receive clinical information of any kind related to such research. As a potential indirect benefit of your participation, your contribution may aid in predicting which patients may respond favorably to treatment or may help in the development of methods for early diagnosis or novel treatments.

Voluntary participation. *Declining to participate shall have NO impact whatsoever on present or future medical care*

Your participation is strictly voluntary. By signing the informed consent document, you indicate that you wish to take part. You may decline to participate or withdraw your consent at any time after you sign. You will not be required to explain the reasons for your withdrawal, and under no circumstances shall the withdrawal of consent have any impact on your present or future medical care.

Withdrawal of consent

If at any time in the future you wish to withdraw your consent, your biological samples will be destroyed along with any related data, and all samples and data will be removed from the biobank. Should you wish to revoke your consent, you must do so in writing, addressing your request to the director of the biobank at the address appearing below.

You may exercise your right to access, change, cancel, or correct this information as well as obtain information on the use of your samples and related data. To do so, contact

Fundación Jiménez Díaz Biobank
Mailing address: Avda. Reyes Católicos nº2 28040
Tel. (+34) 91 5504804
E-mail: biobancofjd@fjd.es

For any questions you may have now or which may arise in the future regarding any aspect related to this consent form, please ask the health-care professional who has provided you with this document. Additionally, you may ask your doctor, who will direct you to the medical staff authorized to handle such matters.

BFJD BIOBANCO Fundación Jiménez Díaz

Hospital Universitario Fundación Jiménez Díaz Grupo quirónsalud

INFORMED CONSENT FOR BIOLOGICAL SAMPLE DONATIONS TO THE FUNDACIÓN JIMÉNEZ DÍAZ BIOBANK

Patient copy

Donor name and surname(s)...
Medical record number…………………….
Staff member providing information...

If you have understood the information you have been given, have obtained answers to your questions, and decide to collaborate with the FJDB according to the terms explained above, please read and sign the form below.

The undersigned hereby authorizes the FJDB to include as part of its resources the excess biological material from the tests he or she has undergone or will undergo as part of the health care being provided. The undersigned also authorizes for this material to be transferred to third parties for the purpose of conducting biomedical research projects, provided these projects have been granted the required approval of the appropriate research ethics committee. In providing this authorization, the undersigned states that he or she has received verbal information on the matter and has read the accompanying information.

______________________	_________________________	___________________
Physician signature	Name in block letters	Date
______________________	_________________________	___________________
Patient signature	Name in block letters	Date
______________________	_________________________	___________________
Witness signature	Name in block letters	Date

I have expressed my wish that the following exceptions be made as concerns the research aims and methods to be carried out when using the samples donated:

………
………
………

INFORMED CONSENT FOR BIOLOGICAL SAMPLE DONATIONS TO THE FUNDACIÓN JIMÉNEZ DÍAZ BIOBANK
Copy for the FJD Biobank

Donor name and surname(s)..
Medical record number…………………….
Staff member providing information..

If you have understood the information you have been given, have obtained answers to your questions, and decide to collaborate with the FJDB according to the terms explained above, please read and sign the form below.
The undersigned hereby authorizes the FJDB to include as part of its resources the excess biological material from the tests he or she has undergone or will undergo as part of the health care being provided. The undersigned also authorizes for this material to be transferred to third parties for the purpose of conducting biomedical research projects, provided these projects have been granted the required approval of the appropriate research ethics committee. In providing this authorization, the undersigned states that he or she has received verbal information on the matter and has read the accompanying information.

______________________	_________________________	___________________
Physician signature	Name in block letters	Date
______________________	_________________________	___________________
Patient signature	Name in block letters	Date
______________________	_________________________	___________________
Witness signature	Name in block letters	Date

I have expressed my wish that the following exceptions be made as concerns the research aims and methods to be carried out when using the samples donated:
………
………
………

INFORMED CONSENT FOR BIOLOGICAL SAMPLE DONATIONS TO THE FUNDACIÓN JIMÉNEZ DÍAZ BIOBANK
Copy for the institution

Donor name and surname(s)..
Medical record number…………………….
Staff member providing information..

If you have understood the information you have been given, have obtained answers to your questions, and decide to collaborate with the FJDB according to the terms explained above, please read and sign the form below.

The undersigned hereby authorizes the FJDB to include as part of its resources the excess biological material from the tests he or she has undergone or will undergo as part of the health care being provided. The undersigned also authorizes for this material to be transferred to third parties for the purpose of conducting biomedical research projects, provided these projects have been granted the required approval of the appropriate research ethics committee. In providing this authorization, the undersigned states that he or she has received verbal information on the matter and has read the accompanying information.

______________________ Physician signature	_________________________ Name in block letters	___________________ Date
______________________ Patient signature	_________________________ Name in block letters	___________________ Date
______________________ Witness signature	_________________________ Name in block letters	___________________ Date

I have expressed my wish that the following exceptions be made as concerns the research aims and methods to be carried out when using the samples donated:
……
……
……
