# Supplementary material for: Cancer-associated fibroblast-derived protein S100-A11 influences the response to anti-HER2 therapies in HER2-positive breast cancer
Source: Neoplasia. 2026 May 19;78:101318. doi: 10.1016/j.neo.2026.101318 (PMC13213234; doi:10.1016/j.neo.2026.101318)
Supplement: Supplementary file 3 [file mmc3.pdf]

INFORME DEL COMITE DE ÉTICA DE LA INVESTIGACION

Dra. Lucía Llanos Jiménez, Secretaria Técnica del COMITE DE ÉTICA DE LA INVESTIGACION DE LA FUNDACION JIMENEZ DIAZ

CERTIFICA:

Que en la reunión del CEIm-FJD que tuvo lugar el 22/02/2022 (acta nº 03/22) y el 22/03/2022 (acta nº 05/22) se evaluó el estudio referido y, ha decidido

A P R O B A R

La propuesta para que se realice el estudio:

Titulo: "PI21/00142. Predicción de la enfermedad residual tras la terapia neoadyuvante en el cáncer de mama HER2 positivo e identificación de estrategias para superar la resistencia**" .**
Investigador Principal: FEDERICO ROJO TODO. Hospital Universitario Fundación Jiménez Díaz
Servicio: Anatomía Patológica
Promotor: FIIS-FJD
Código: PI21/00142
Documentos con versiones:
PROTOCOLO Versión de Marzo de 2022

Además, hace constar que:

1. En dicha reunión se cumplieron los requisitos establecidos en la legislación vigente –Decreto 39/94 de la CAM– para que la decisión del citado CEIm sea válida.

2. El Estudio reúne las normas éticas estándar de nuestra Institución para la realización de este tipo de estudios.

3. Se cumplen los preceptos éticos formulados en la Declaración de Helsinki de la Asociación Médica mundial sobre principios éticos para las investigaciones médicas en seres humanos y en sus posteriores revisiones, así como aquellos exigidos por la normativa aplicable en función de las características del estudio.

4. El CEImFJD, tanto en su composición como en sus procedimientos, cumple con las normas de BPC (CPMP/ICH/135/95) y con la legislación vigente que regula su funcionamiento, y que la composición del CEIm FJD es la indicada en el anexo I, teniendo en cuenta que en el caso de que algún miembro participe en el estudio o declare algún conflicto de interés no habrá participado en la evaluación ni en el dictamen.

5. Asimismo, hacemos constar que existe contraprestación económica para el centro, según convocatoria de la ayuda.

6. Además, este comité recuerda al Promotor la obligación, en el caso de que se trate de un estudio prospectivo, de realizar el registro del estudio en una base de datos de acceso público antes de reclutar el primer paciente así como el seguimiento del estudio de acuerdo a la legislación vigente.

Lo que firmo en Madrid a 22/03/2022

FUNDACIÓN JIMÉNEZ DÍAZ COMITÉ DE INVESTIGACIÓN CLÍNICA MADRID

Dra. Lucía Llanos Jiménez
Secretaria Técnica CEImFJD

Anexo I

PIC047-22_FJD

COMPOSICIÓN DEL CEIm

Presidente:
JAVIER BECARES MARTÍNEZ (Farmacéutico - Hospital Universitario Fundación Jiménez Díaz)
Vicepresidente:
MACARENA BONILLA PORRAS (Farmacéutico - Hospital Universitario Fundación Jiménez Díaz)
Secretario:
LUCÍA LLANOS JIMÉNEZ (Farmacóloga - Miembro Comité de Investigación - Hospital Universitario Fundación Jiménez Díaz)

Vocales:
NICOLÁS ALEJANDRE ALBA (Médico Asistencial - Oftalmología - Hospital Universitario Fundación Jiménez Díaz)
MIRIAM BLANCO RODRÍGUEZ (Médico Asistencial - Pediatría - Hospital Universitario Fundación Jiménez Díaz)
ALFONSO CABELLO UBEDA (Médico Asistencial - Medicina Interna - Hospital Universitario Fundación Jiménez Díaz)
RAÚL CÓRDOBA MASCUÑANO (Médico Asistencial - Hematología - Hospital Universitario Fundación Jiménez Díaz)
BERNARD GASTON DOGER DE SPEVILLE URIBE (Médico Asistencial - Oncología - Hospital Universitario Fundación Jiménez Díaz)
ANA GARCIA DIAZ (Abogado-a)
MARÍA DEL MAR JIMENEZ DEL CASTILLO (DUE - Hospital Universitario Fundación Jiménez Díaz)
GONZALO PIZARRO SÁNCHEZ (Médico Asistencial - Cardiología - Hospital Universitario Quirón Madrid)
FRANCISCO JAVIER RUIZ HORNILLOS (Médico Asistencial. Miembro Comité de Investigación y Miembro del Comité de Ética Asistencial - Hospital Universitario Infanta Elena)
OLGA SANCHEZ PERNAUTE (Médico Asistencial - Reumatología / Bioquímica Clínica - Hospital Universitario Fundación Jiménez Díaz)
ANA DÍEZ ALCÁNTARA (Farmacéutico de Atención Primaria. Dirección Asistencial Noroeste)
SERGIO HOYOS SIMÓN (Médico Asistencial - Oncología - Hospital Universitario Rey Juan Carlos)
SANDRA ZAZO HERNÁNDEZ (Bióloga Asistencial - Miembro Comité de Investigación - Anatomía Patológica - Hospital Universitario Fundación Jiménez Díaz)
ROSA SANCHEZ HERNANDEZ (Médico Asistencial - Nefrología - Hospital General de Villalba)
LORENA PINGARRON MARTÍN (Médico Asistencial - Miembro Comité de Investigación - Cirugía Maxilofacial / Ortodoncia - Hospital Universitario Rey Juan Carlos)
MARTA MARIN CRESPO (Abogado-a)
FERNANDO ABELLAN-GARCIA SANCHEZ (Abogado-a)
MIGUEL MIR CORDERO (Lego no vinculado a la Institución)
CAROLINA GOTERA RIVERA (Médico Asistencial - Neumología - Hospital Universitario Fundación Jiménez Díaz)
MONTIEL JIMÉNEZ FUENTES (Médico Asistencial - Cirugía Digestivo / General - Hospital Universitario Fundación Jiménez Díaz)

Anexo II
CENTROS E INVESTIGADORES PRINCIPALES Y COLABORADORES

Titulo: “PI21/00142. Predicción de la enfermedad residual tras la terapia neoadyuvante en el cáncer de mama HER2 positivo e identificación de estrategias para superar la resistencia**” .**
Investigador Principal: FEDERICO ROJO TODO. Hospital Universitario Fundación Jiménez Díaz
Promotor: FIIS-FJD
Código: PI21/00142
Documentos con versiones:
PROTOCOLO Versión de Marzo de 2022

Fecha de actualizacion del anexo II: **22/03/2022**

FEDERICO ROJO TODO. Hospital Universitario Fundación Jiménez Díaz. IP
JUAN MADOZ GURPIDE. Hospital Universitario Fundación Jiménez Díaz. Colaborador/a
ALICIA CAZORLA JIMENEZ. Hospital Universitario Fundación Jiménez Díaz. Colaborador/a
SANDRA ZAZO HERNANDEZ. Hospital Universitario Fundación Jiménez Díaz. Colaborador/a
NEREA CARVAJAL GARCIA. Hospital Universitario Fundación Jiménez Díaz. Colaborador/a
MELANIA LUQUE MARIN. Hospital Universitario Fundación Jiménez Díaz. Colaborador/a
YANN IZARZUGAZA PERON. Hospital Universitario Fundación Jiménez Díaz. Colaborador/a
MARTA SANZ ALVAREZ. Hospital Universitario Fundación Jiménez Díaz. Colaborador/a
NATALIA RAMIREZ MERINO. Hospital Universitario Infanta Elena. Colaborador/a

PIC047-22_FJD
