# Supplementary material for: Cancer-associated fibroblast-derived protein S100-A11 influences the response to anti-HER2 therapies in HER2-positive breast cancer
Source: Neoplasia. 2026 May 19;78:101318. doi: 10.1016/j.neo.2026.101318 (PMC13213234; doi:10.1016/j.neo.2026.101318)
Supplement: Supplementary file 4 [file mmc4.docx]

**Figures**

**Figure 1B**

**BT-474**

**p-stat3:**


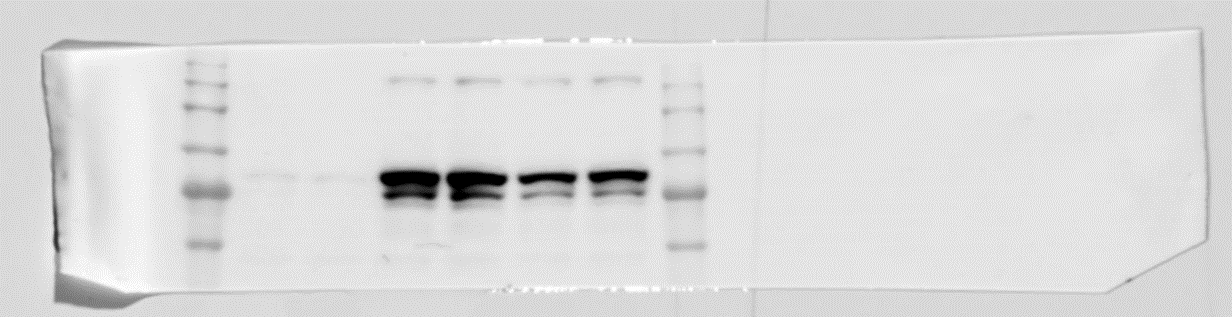


**STAT3:**


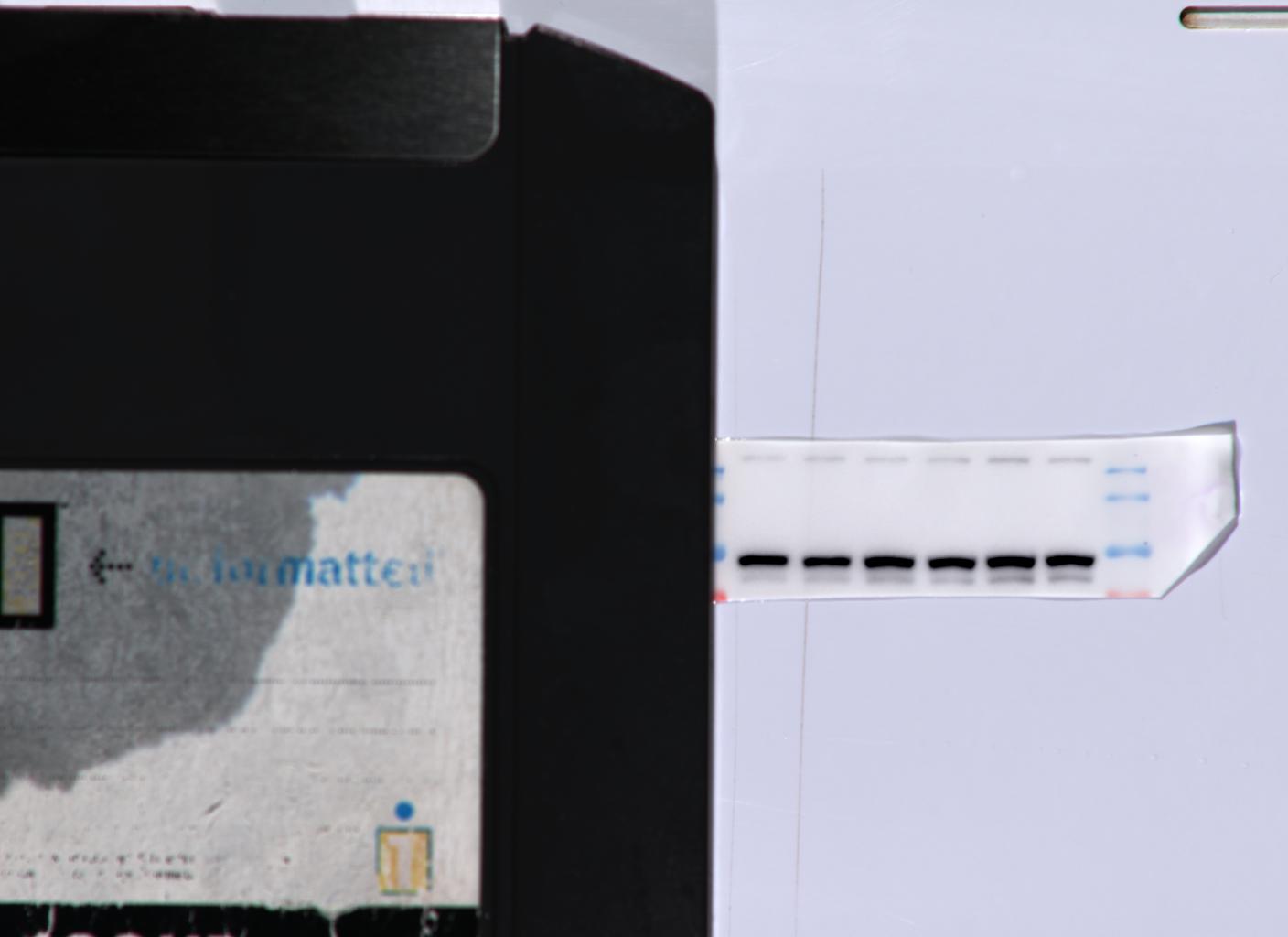


**p-AKT(Thr308):**

**
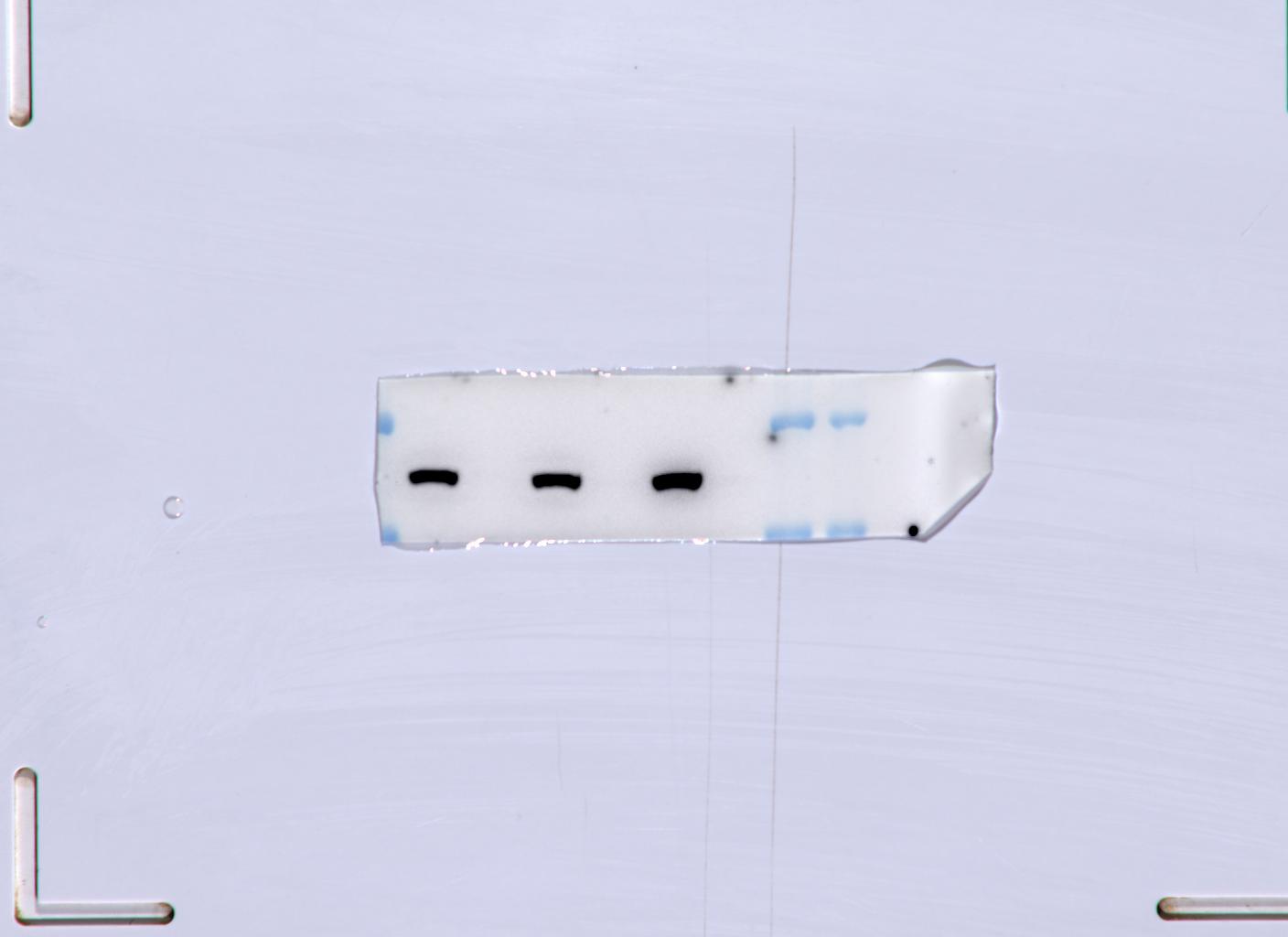
**

**p-AKT(Ser473):**

**
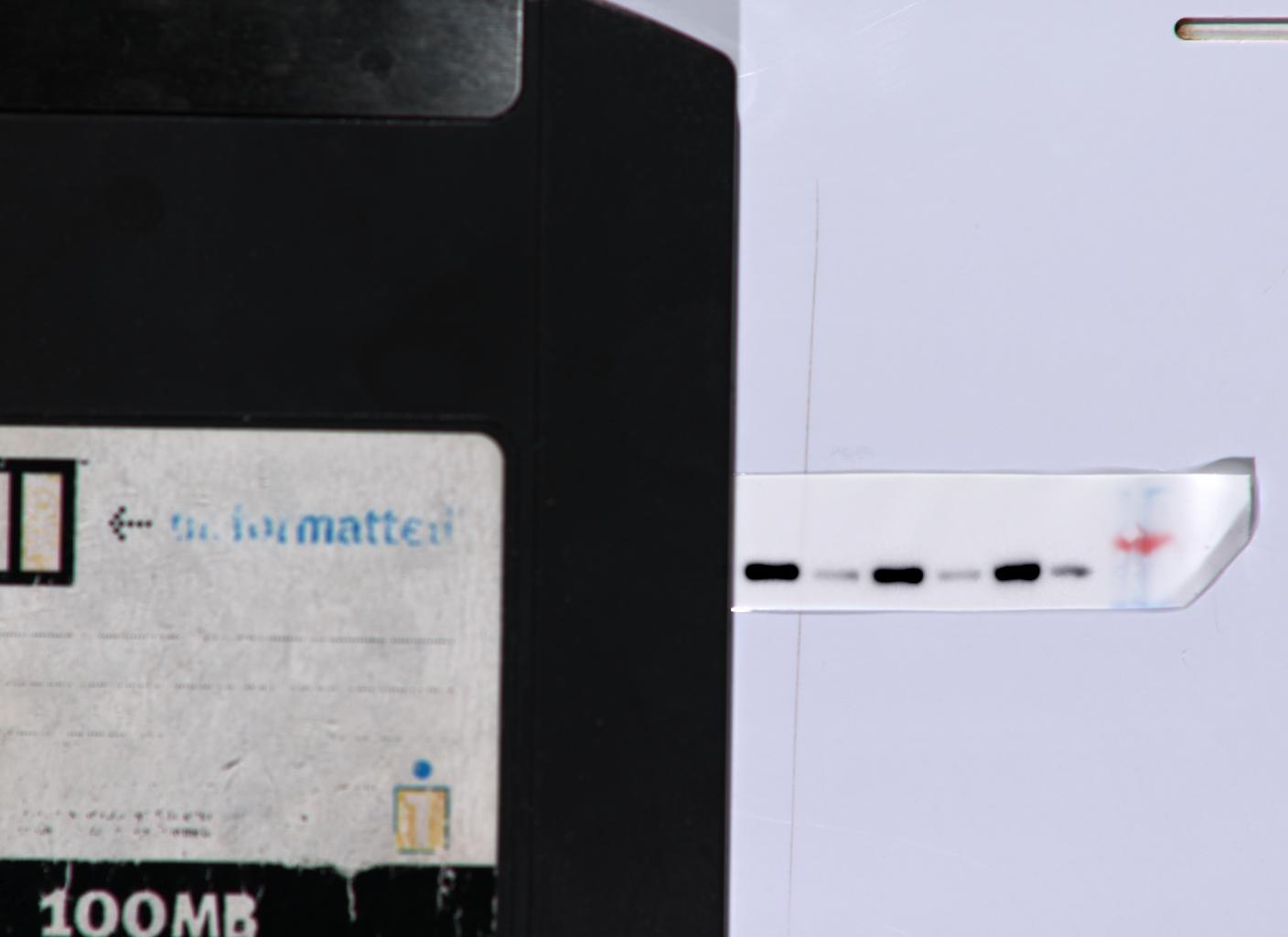
**

**AKT:**

**
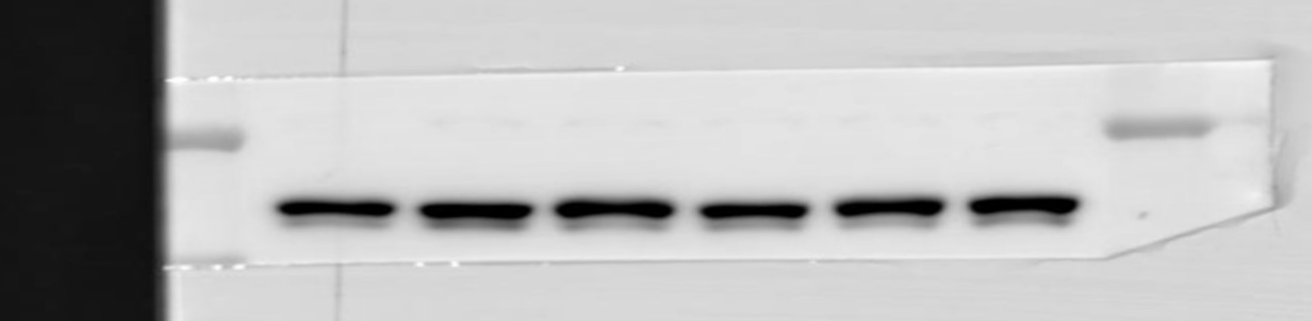
**

**p-ERK:**

**
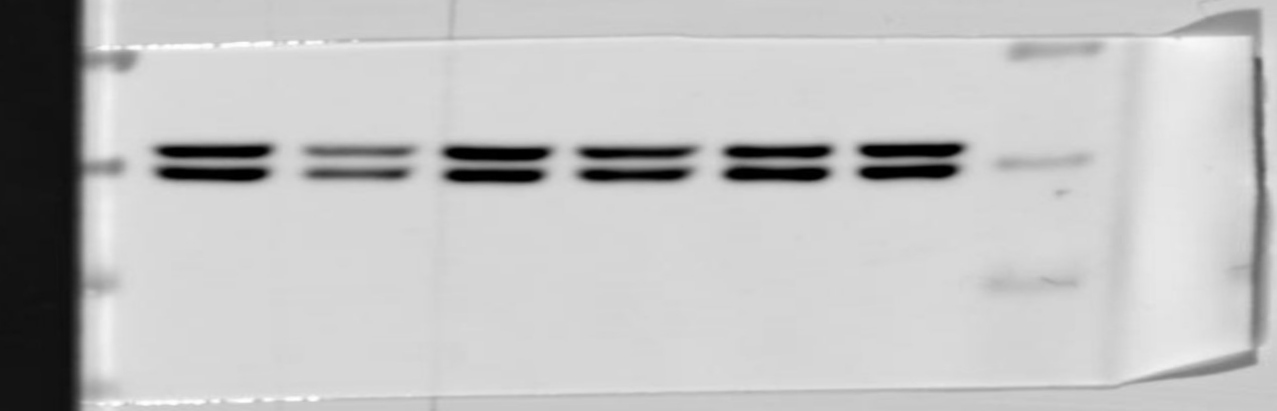
**

**ERK:**

**
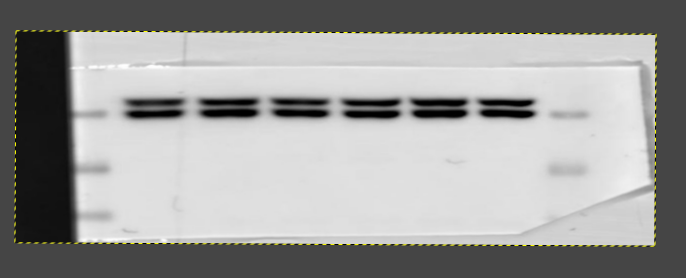
**

**Beta-actina:**

**
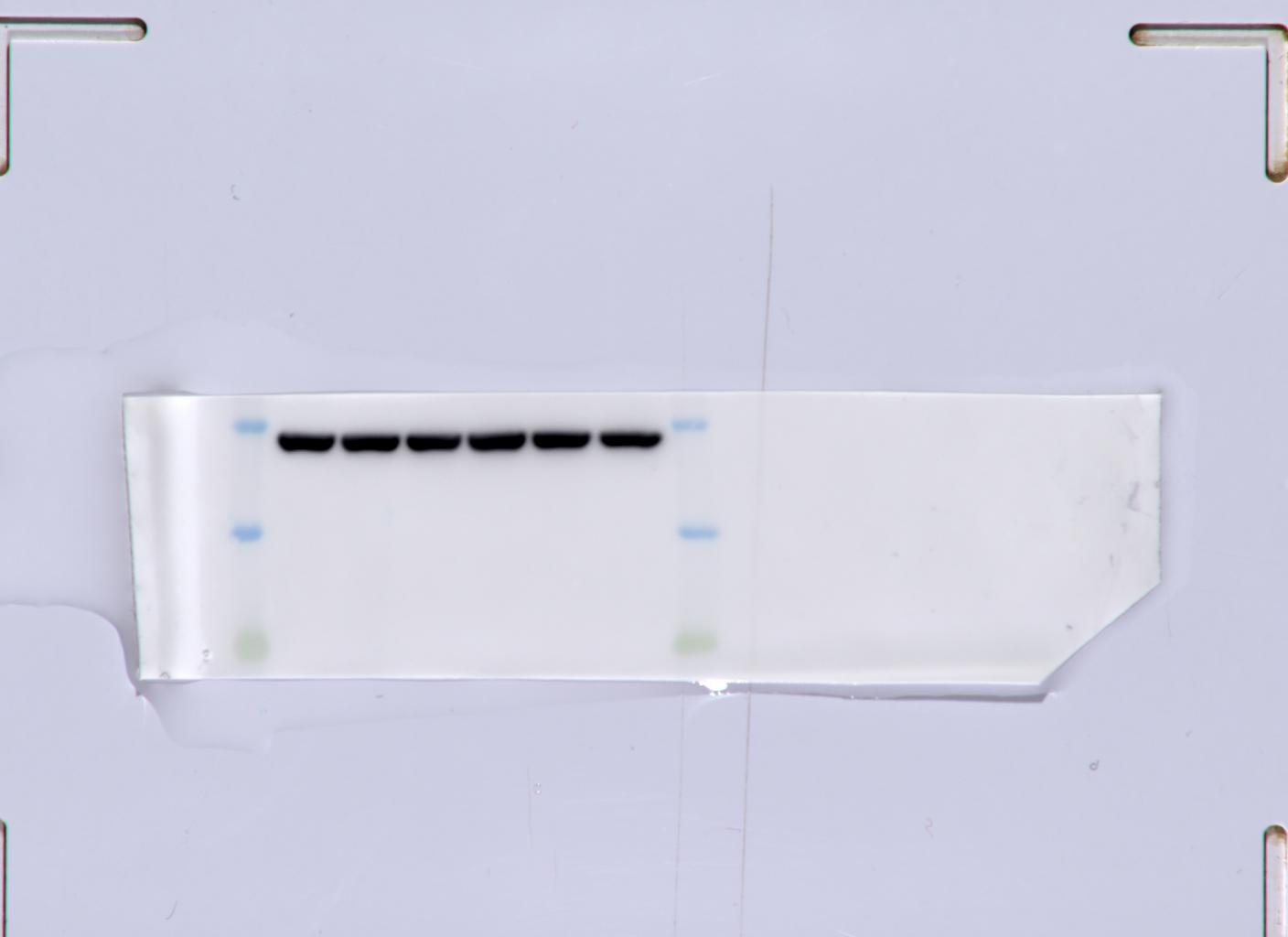
**

**Figure 1B**

**EFM-192A**

**p-STAT3:**

**
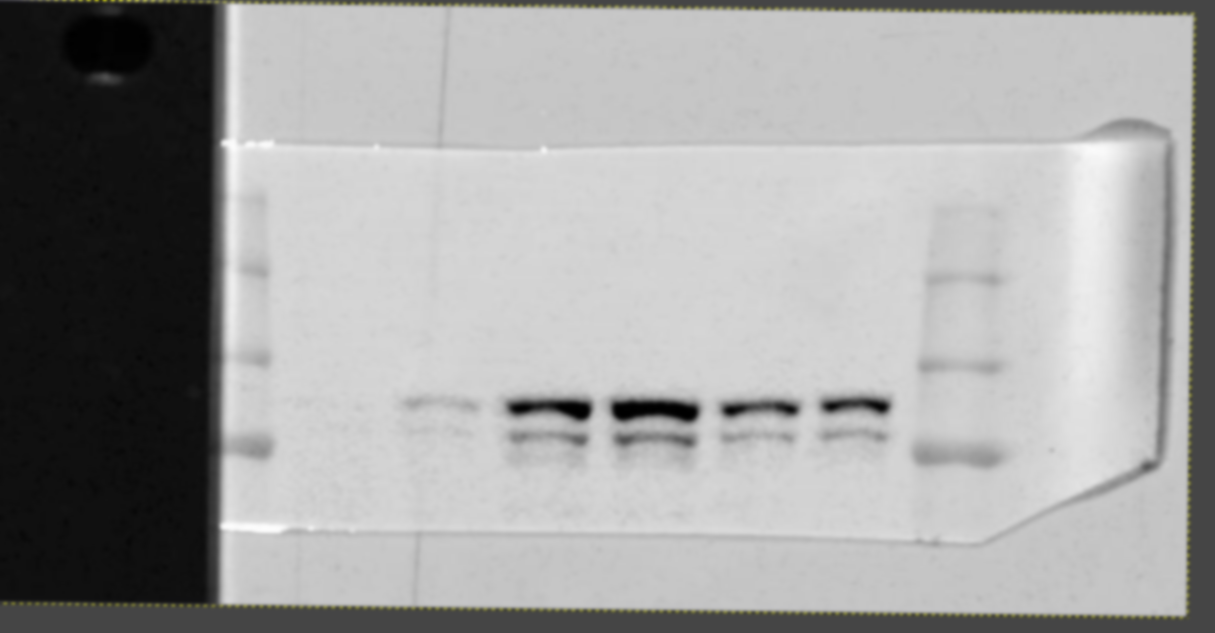
**

**STAT3:**

**
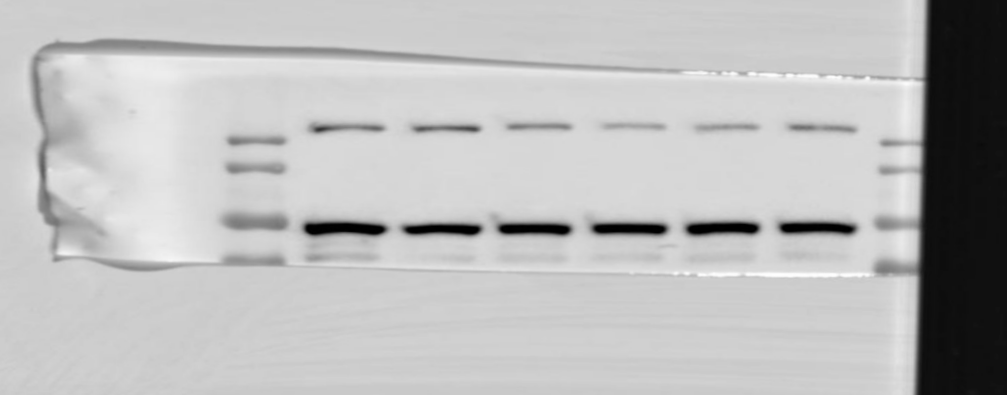
**

**p-AKT(Thr308):**

**
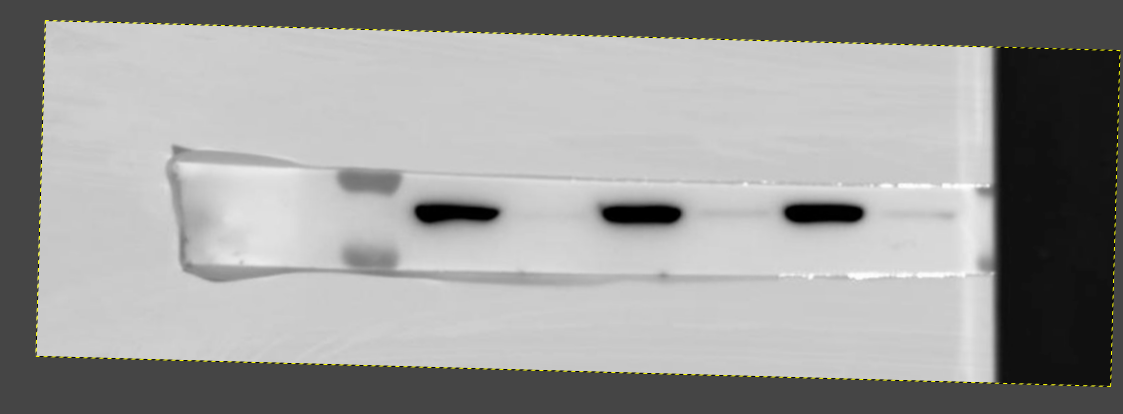
**

**p-AKT(Ser473):**

**
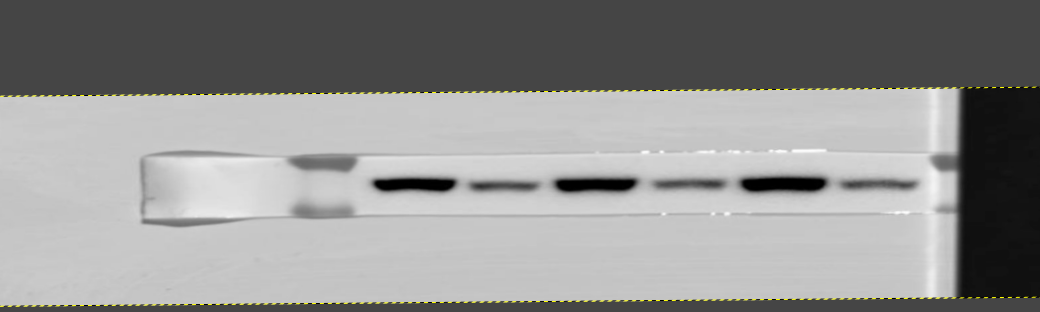
**

**AKT:**

**
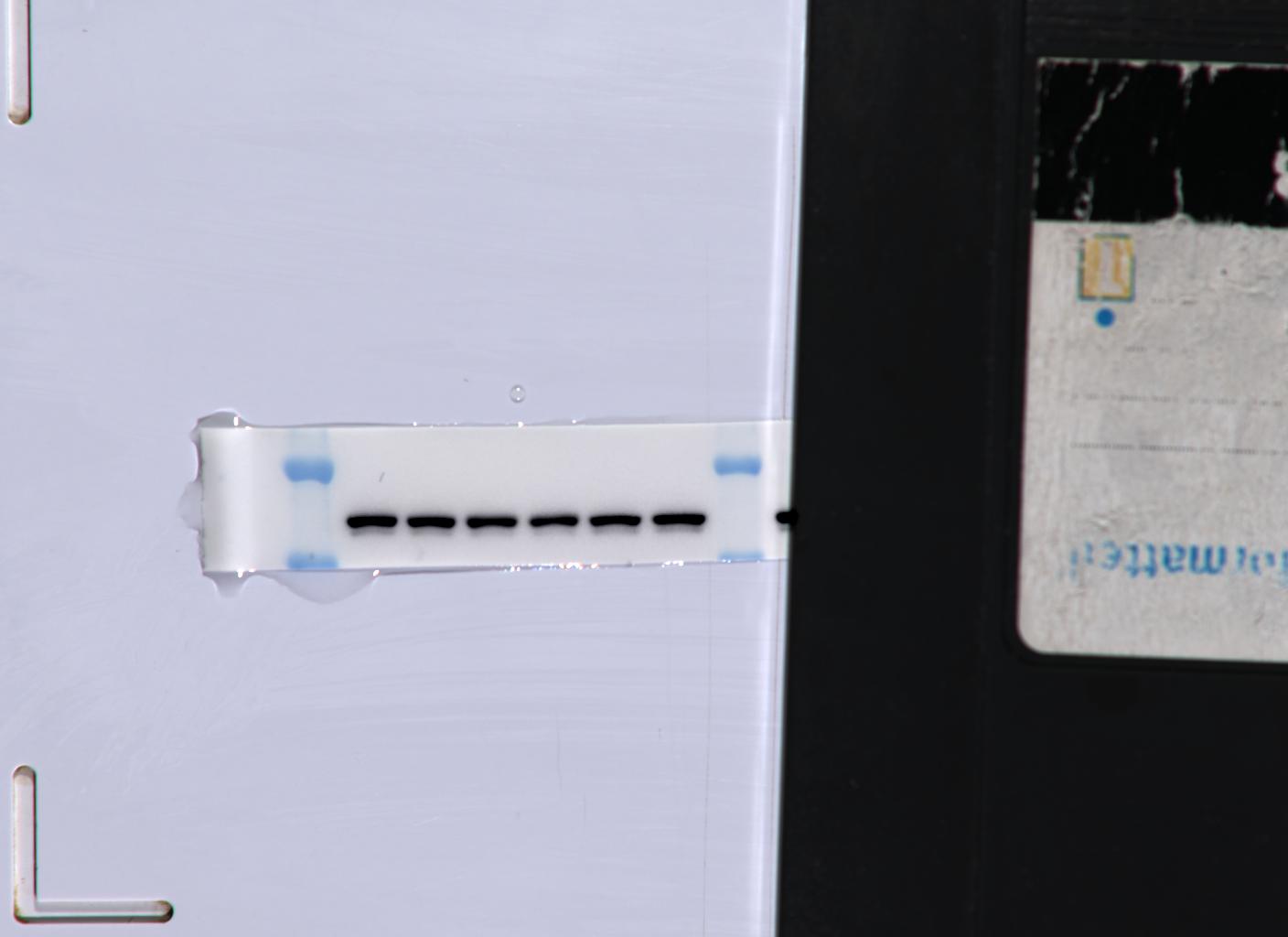
**

**p-ERK:**

**
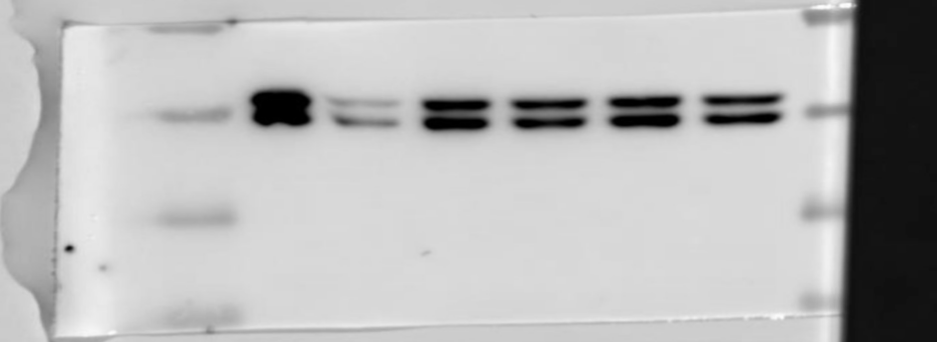
**

**ERK:**

**
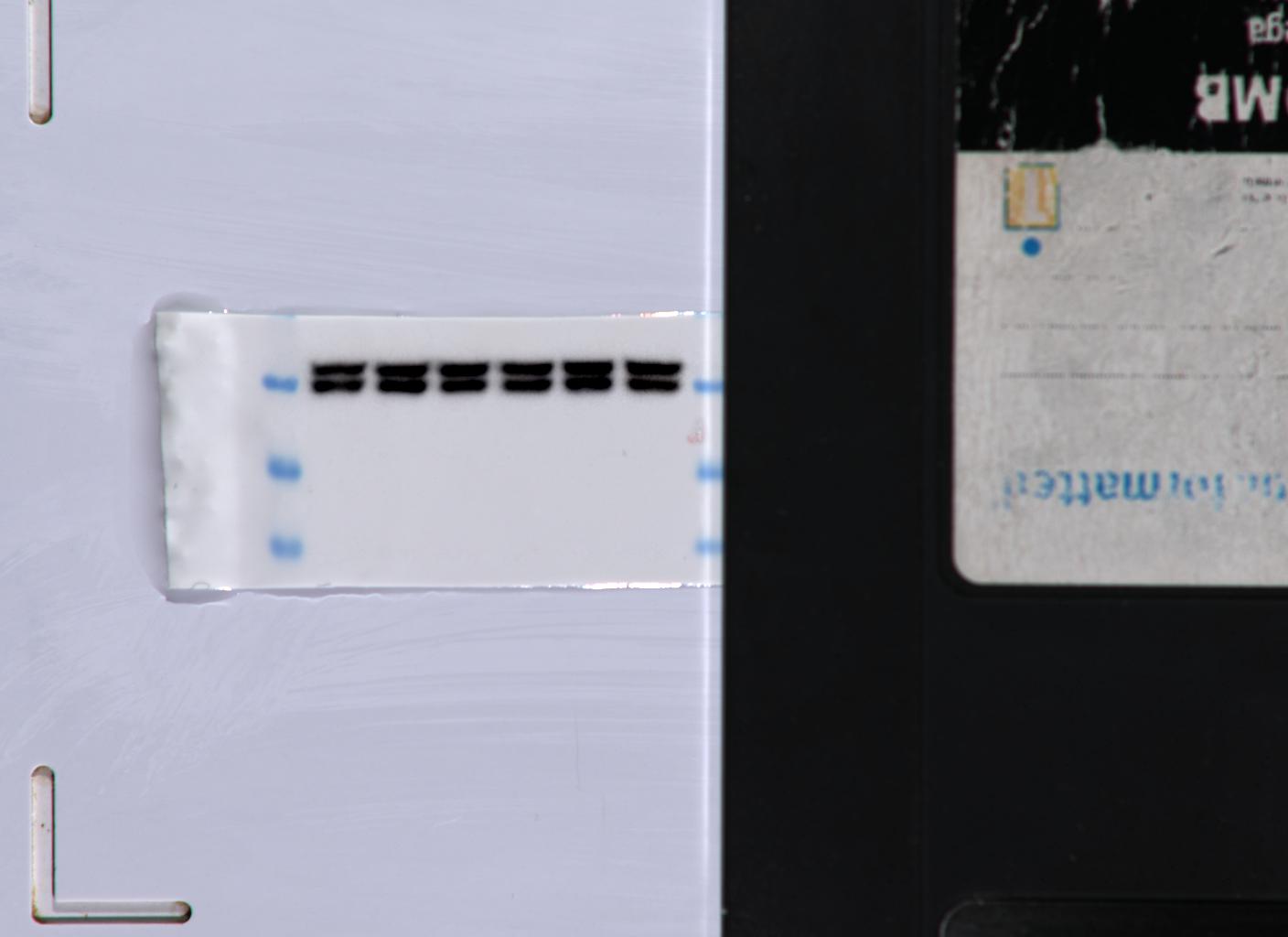
**

**Beta-actina:**

**
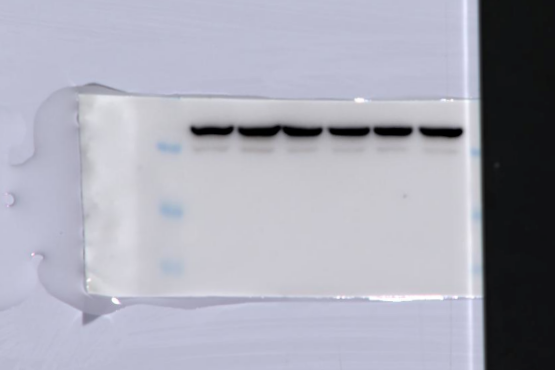
**

**Figure 2B**

**BT-474**

**p-STAT3:**

**
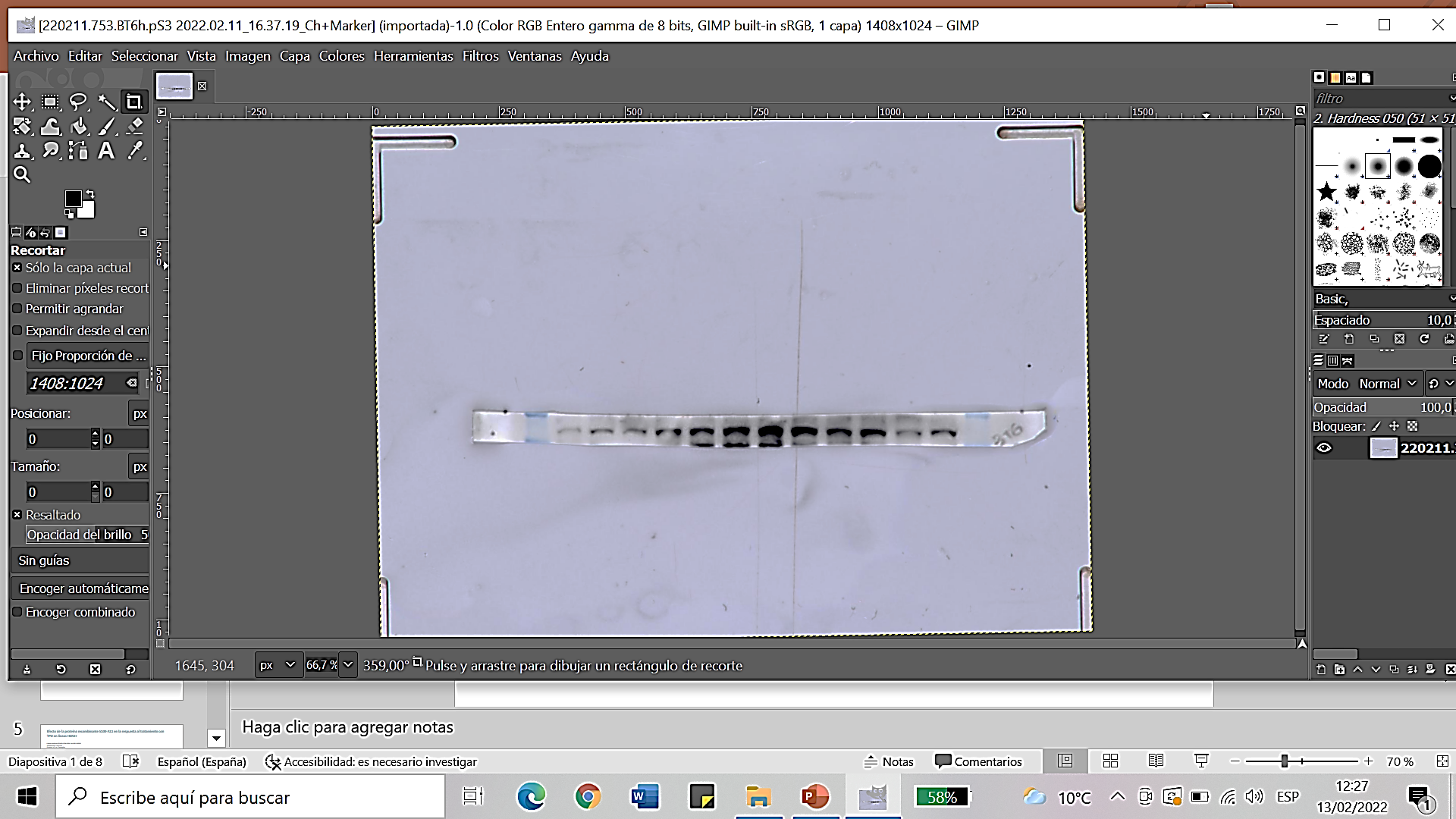
**

**STAT3:**

**
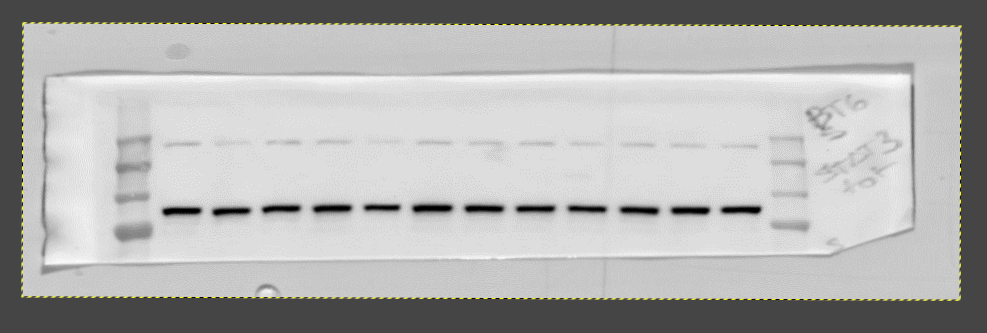
**

**p-AKT(Thr308):**

**
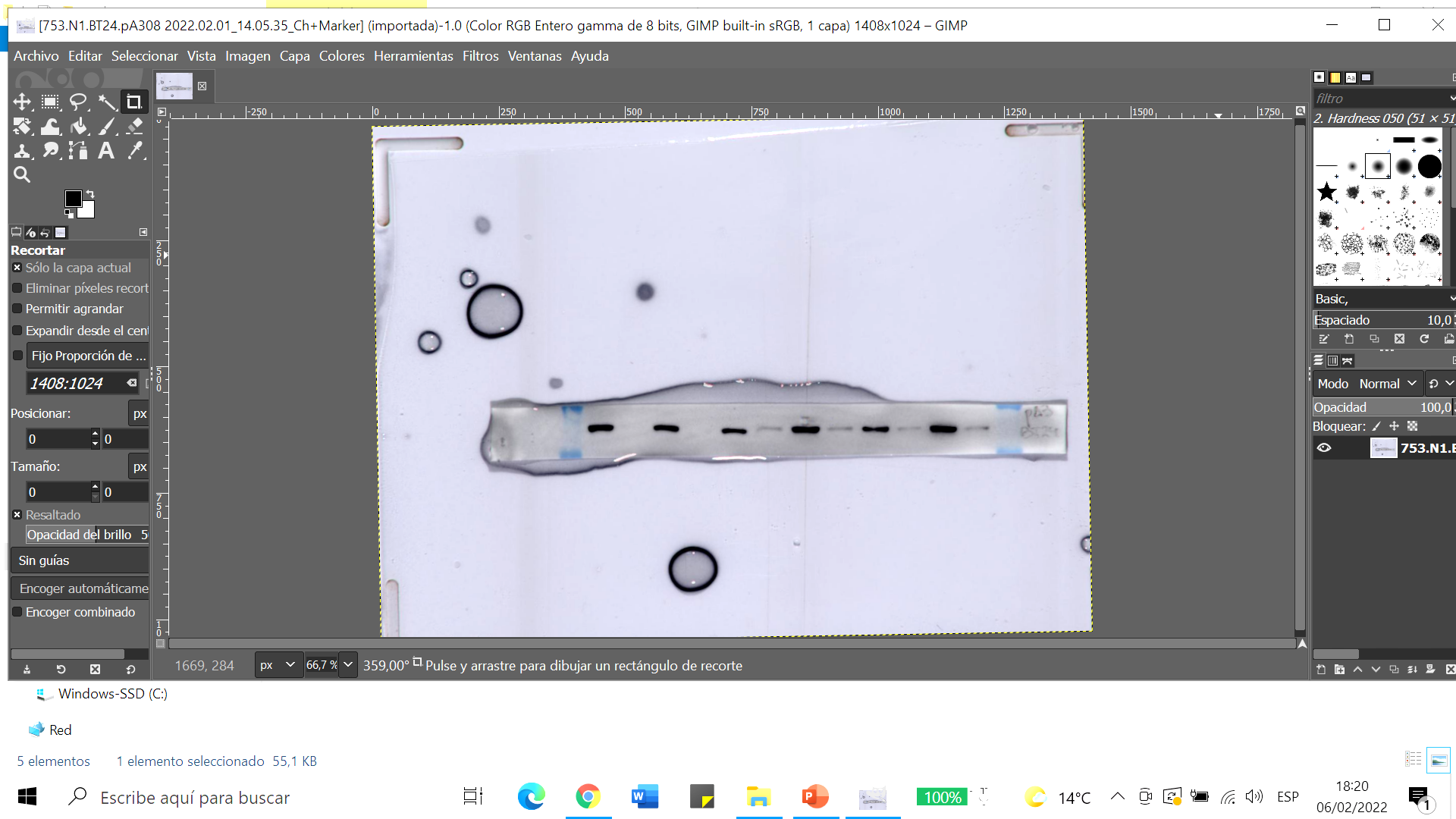
**

**p-AKT(Ser473):**

**
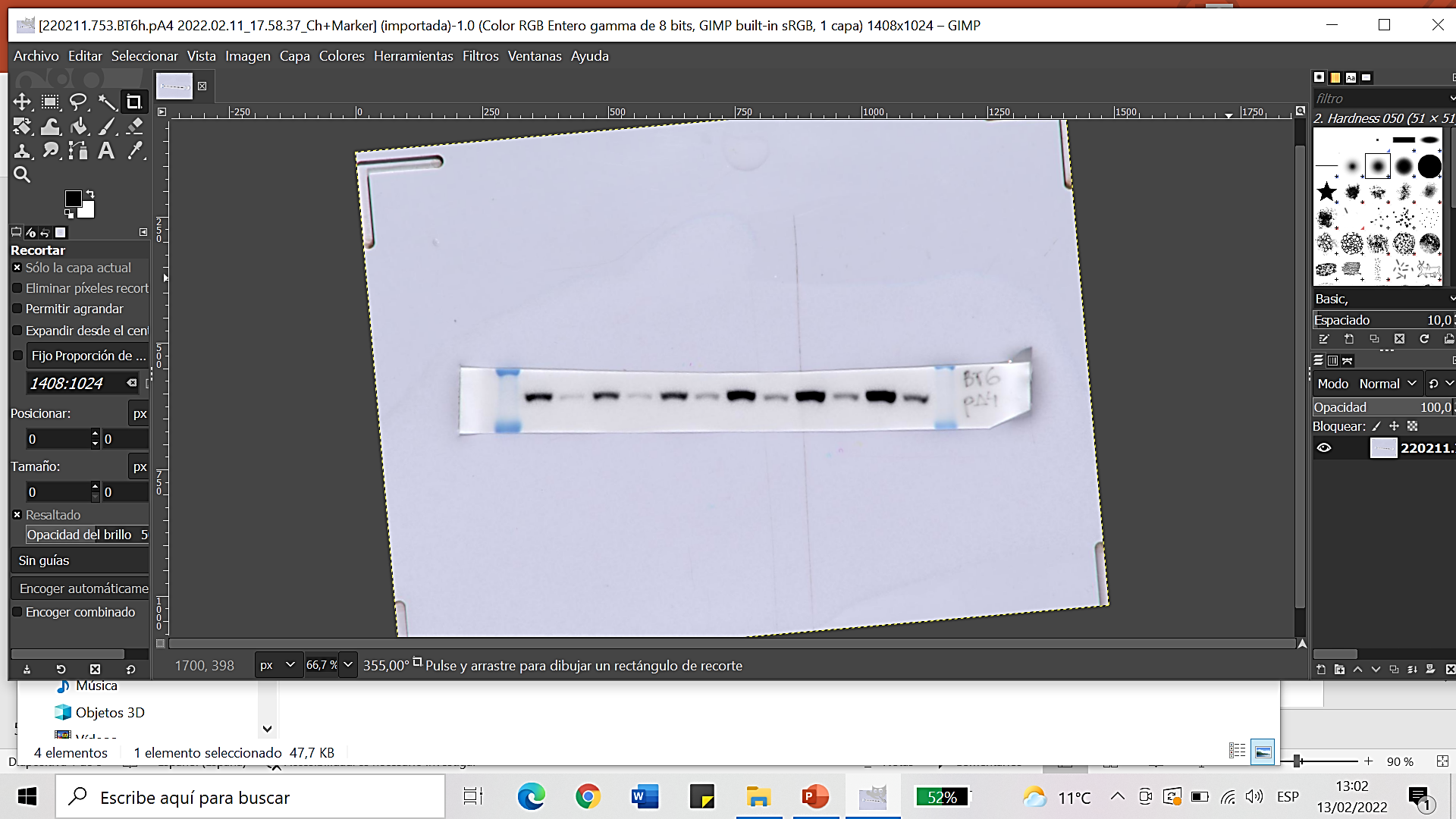
**

**AKT:**

**
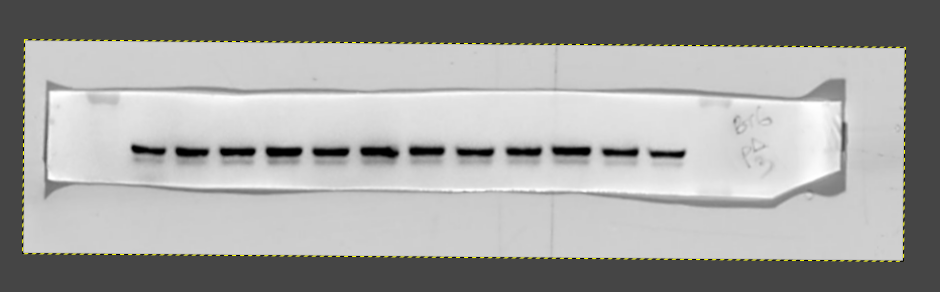
**

**p-ERK:**

**
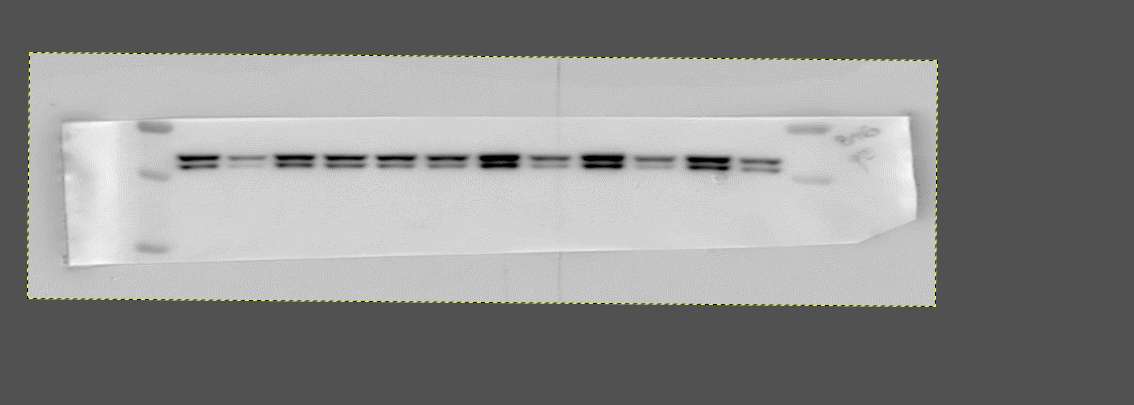
**

**ERK:**

**
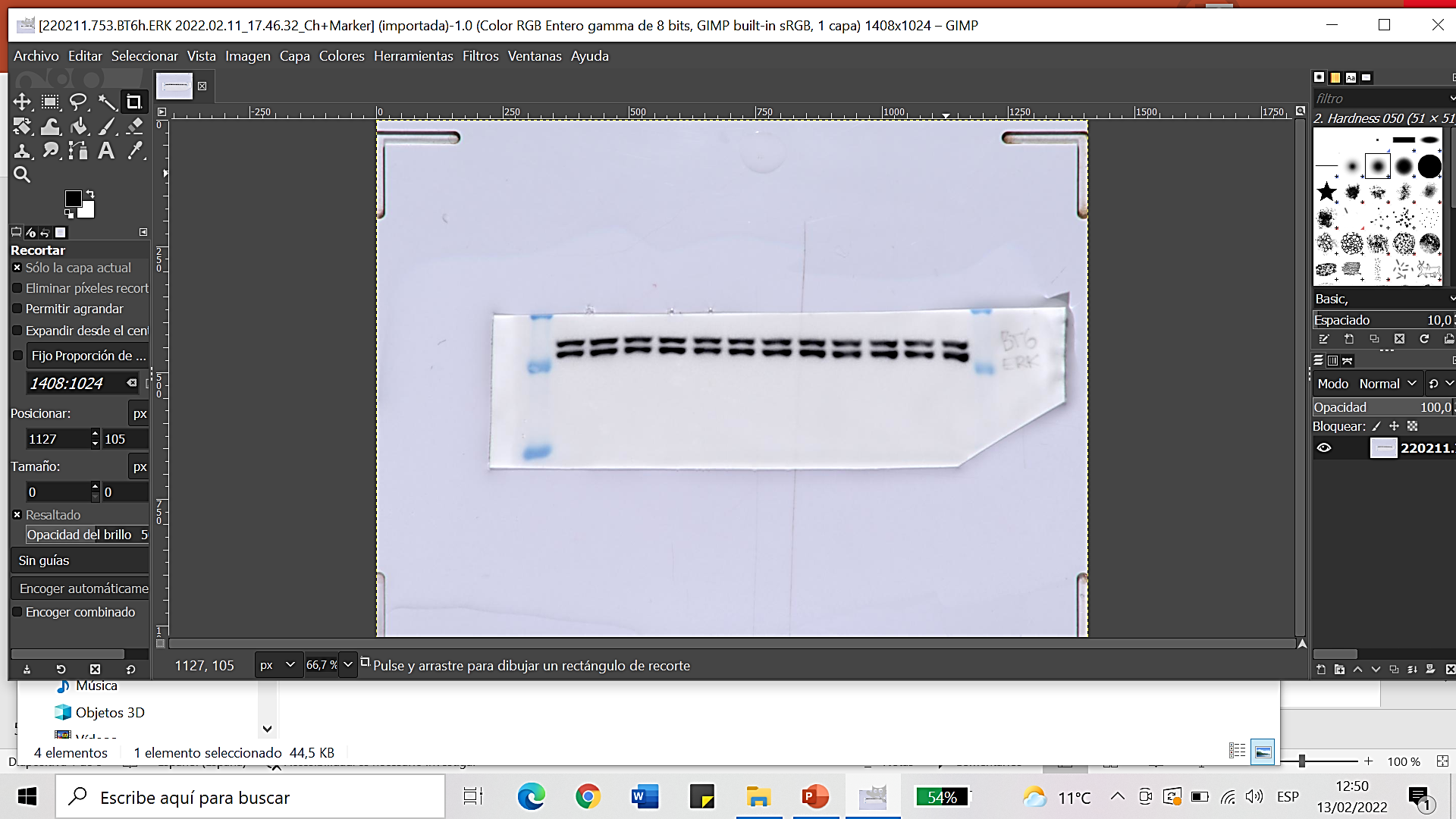
**

**Beta-actina:**

**
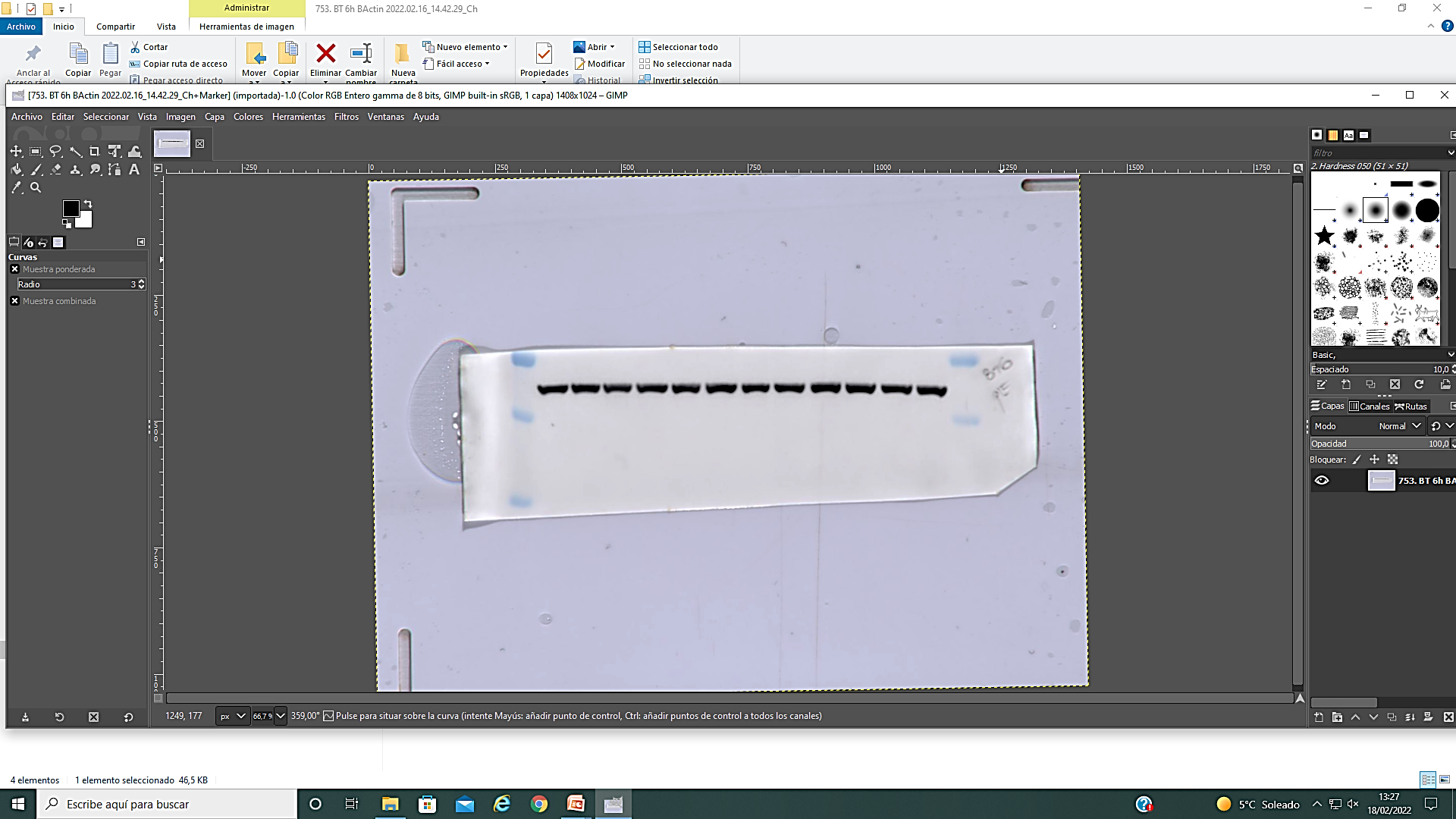
**

**Figure 2B**

**EFM-192A**

**p-STAT3:**

**
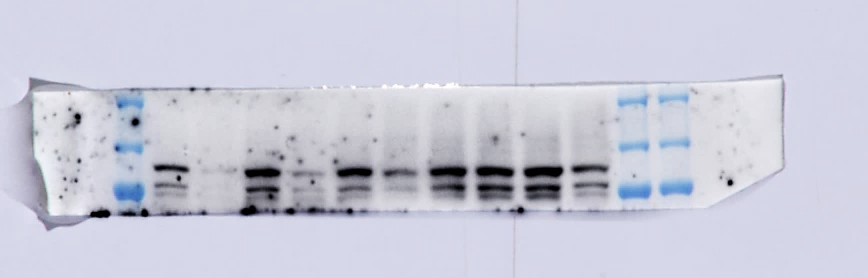
**

**STAT3:**

**
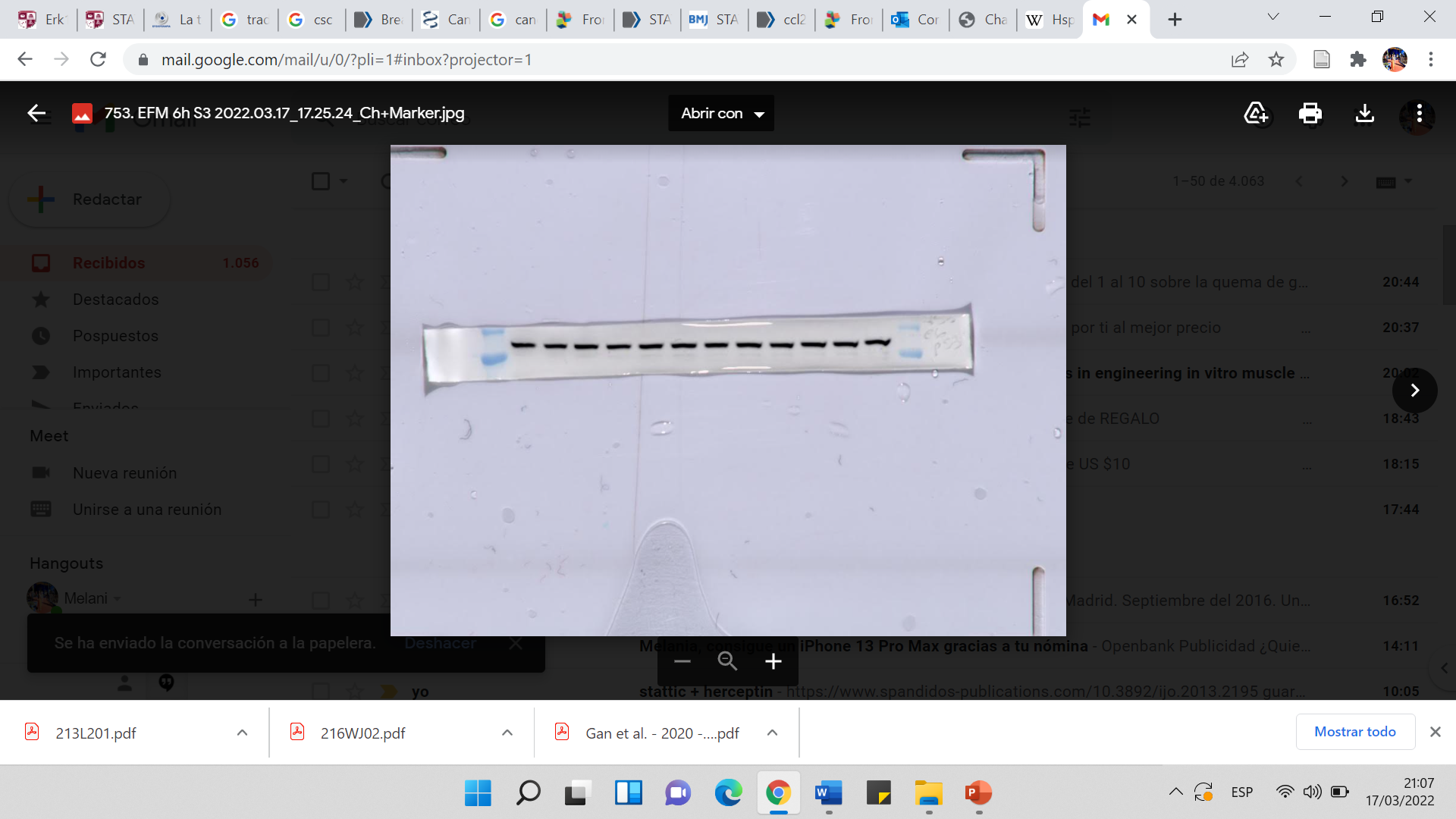
**

**p-AKT(Thr308):**

**
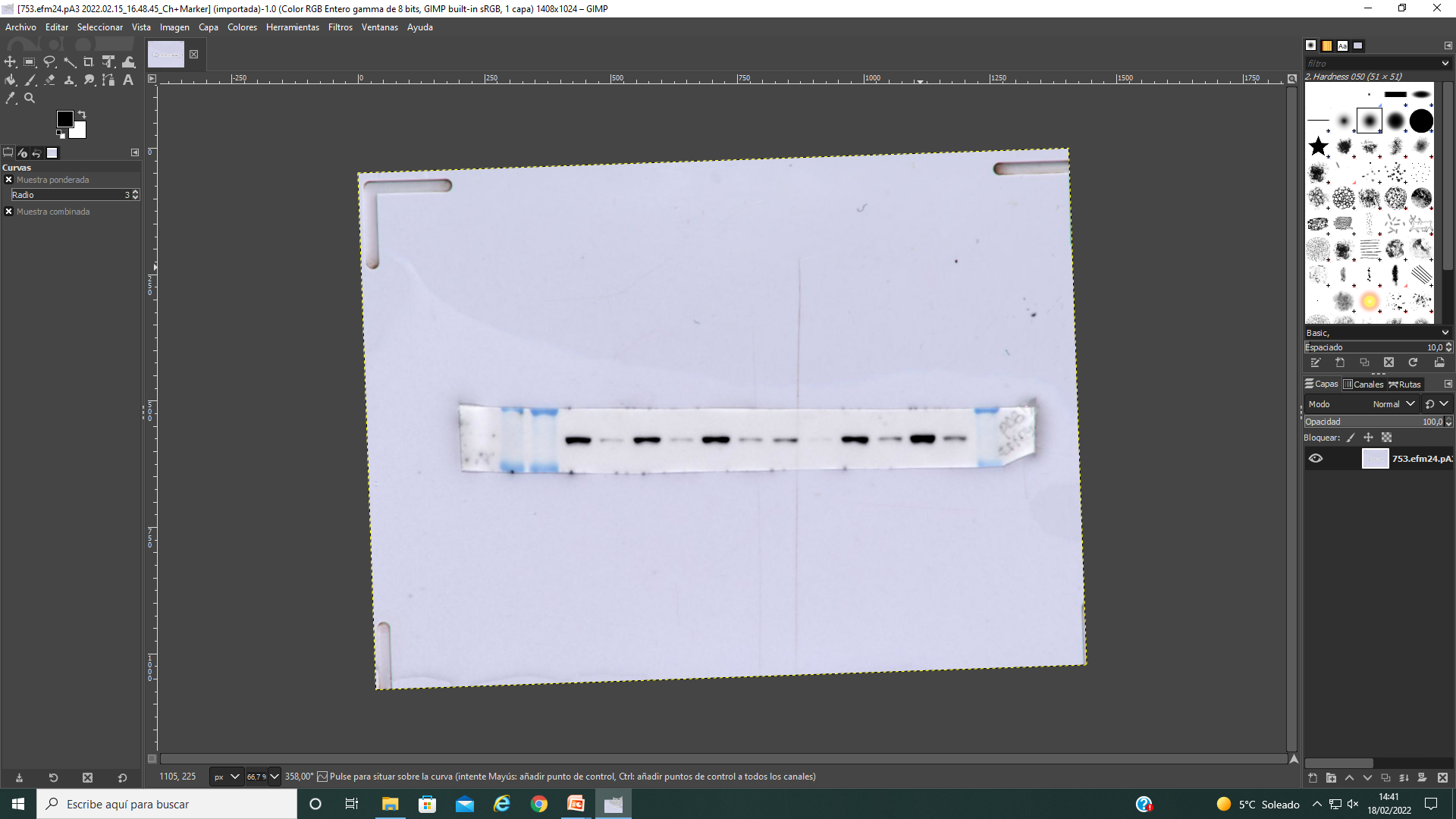
**

**p-AKT(Ser473):**

**
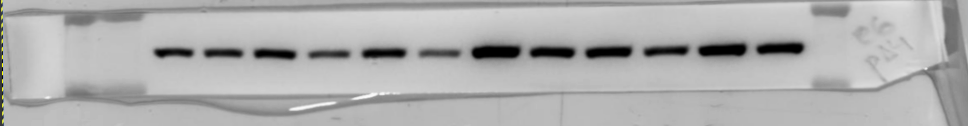
**

**AKT:**

**
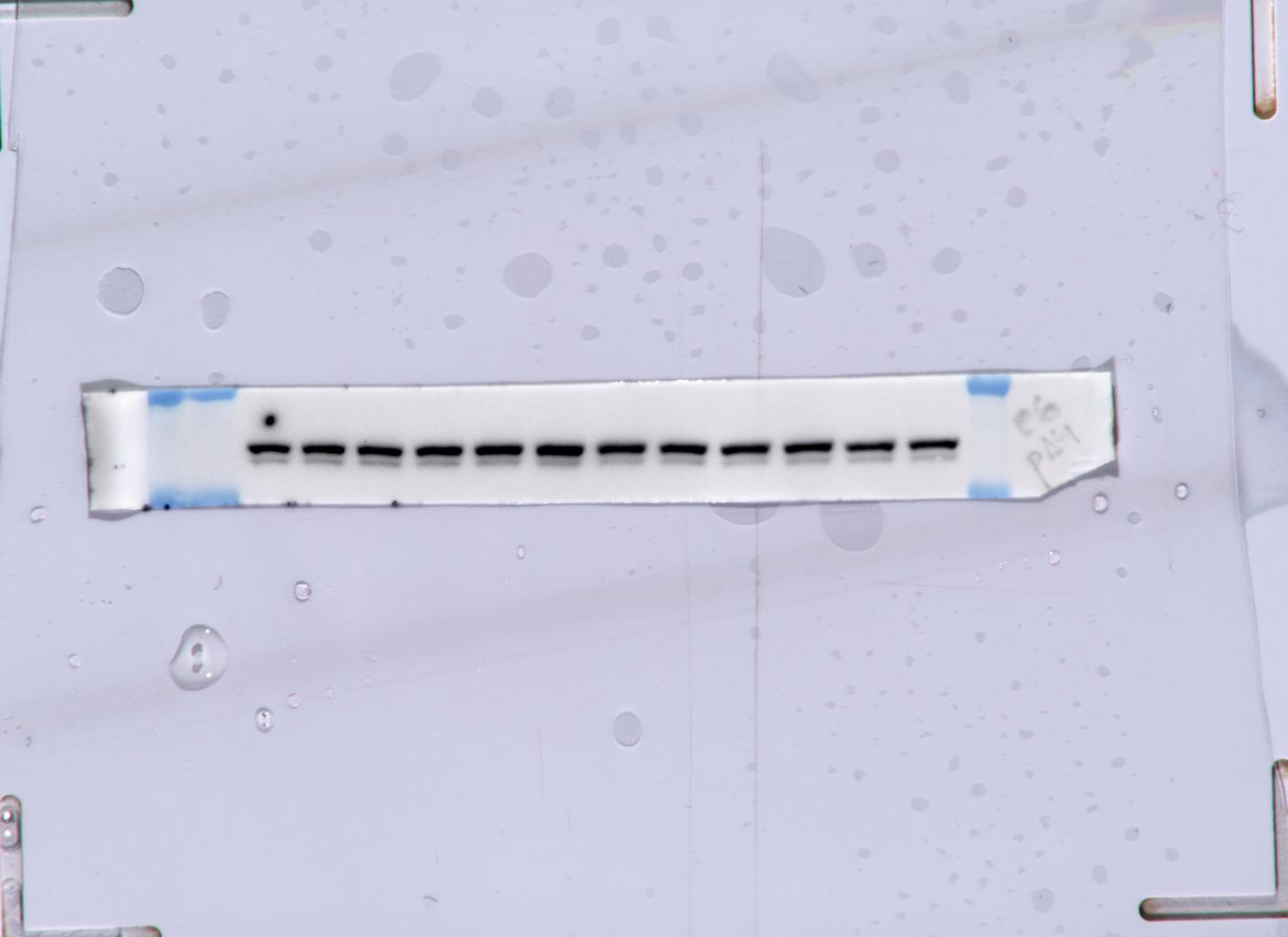
**

**p-ERK:**

**
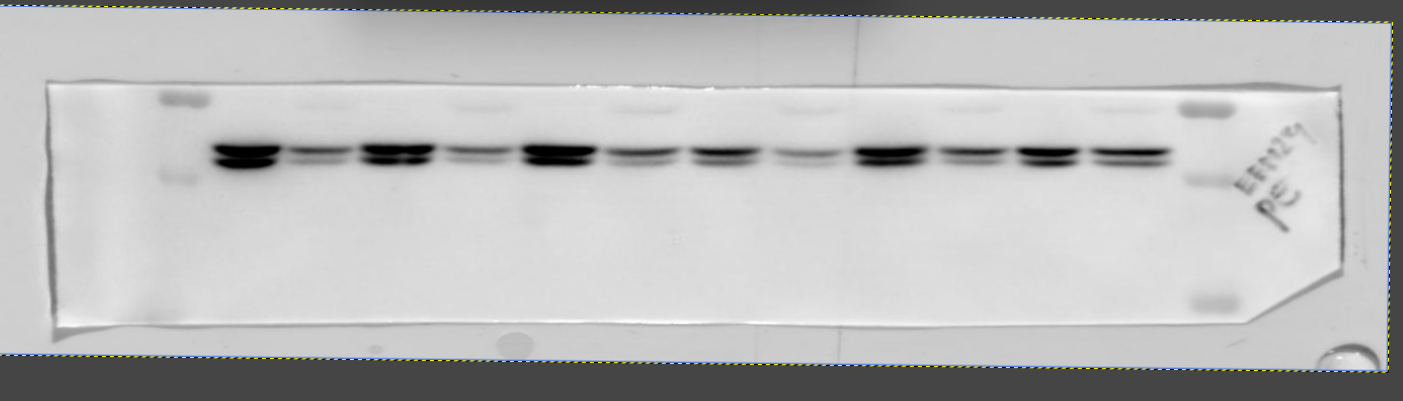
**

**ERK:**

**
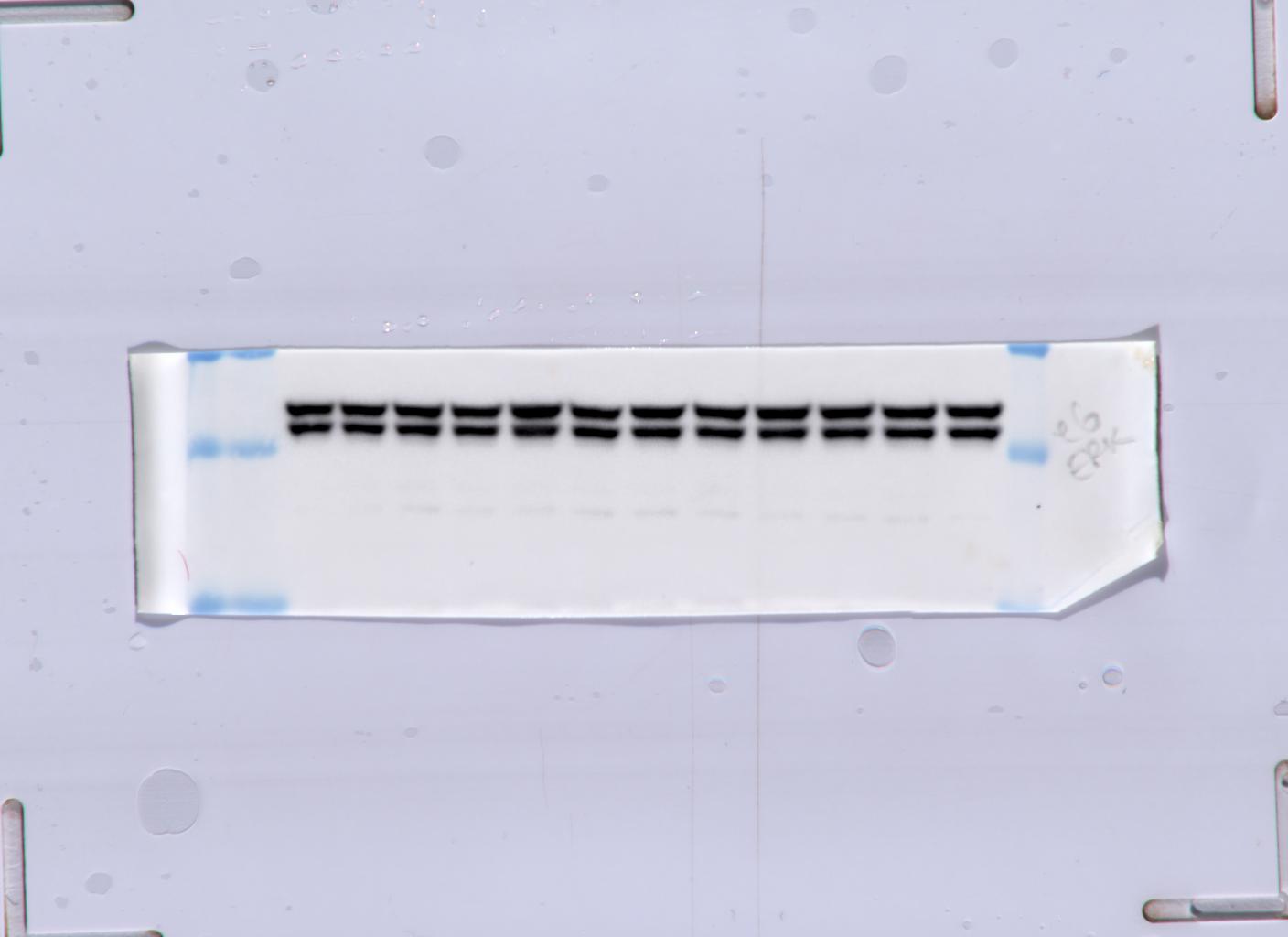
**

**Beta-actina:**

**
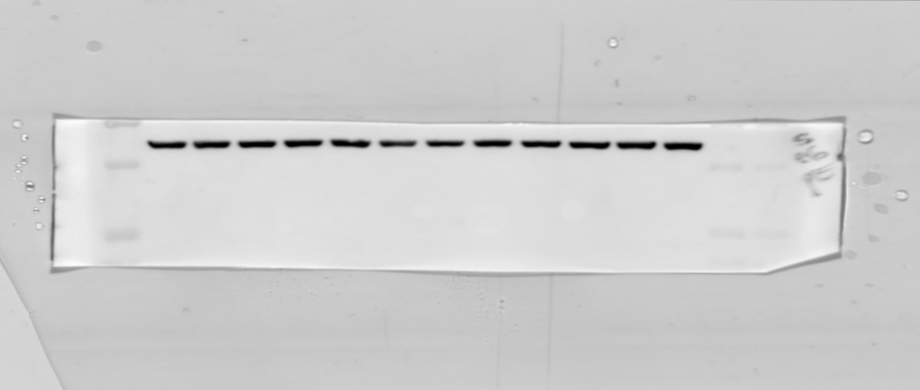
**

**Figure 4B**

**BT-474**

**Basal**

**no TPD TPD**

**p-STAT3:**

**
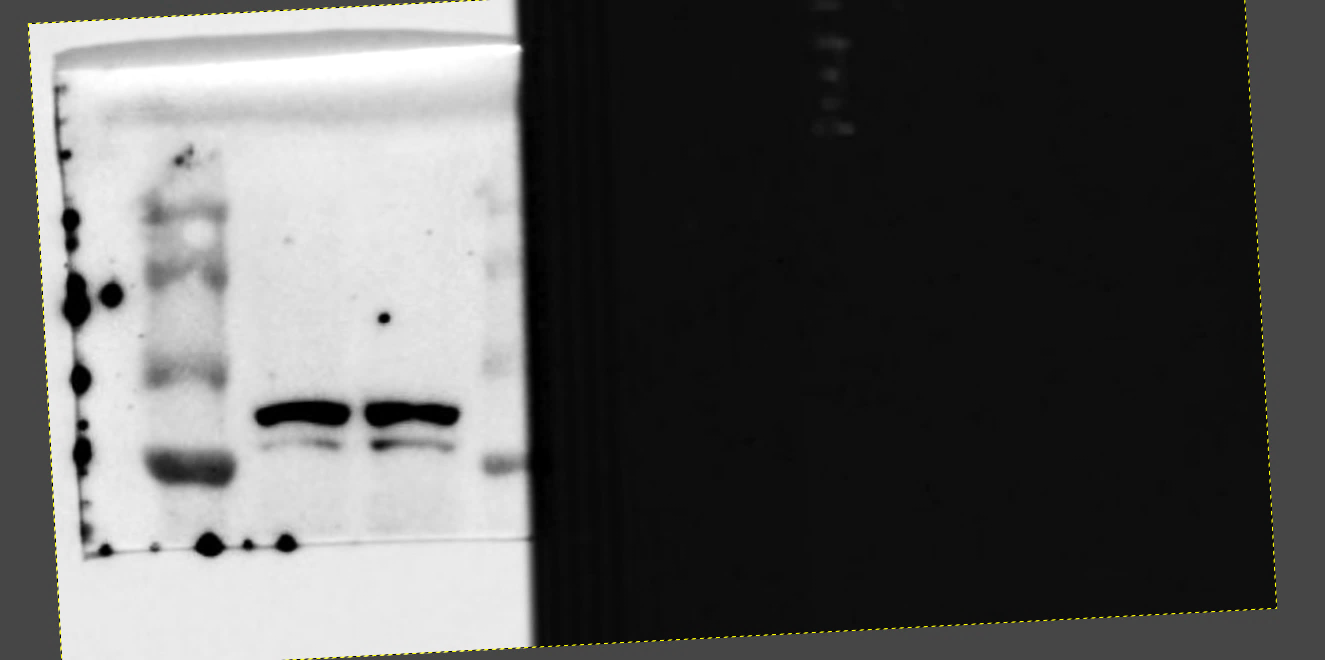
** **
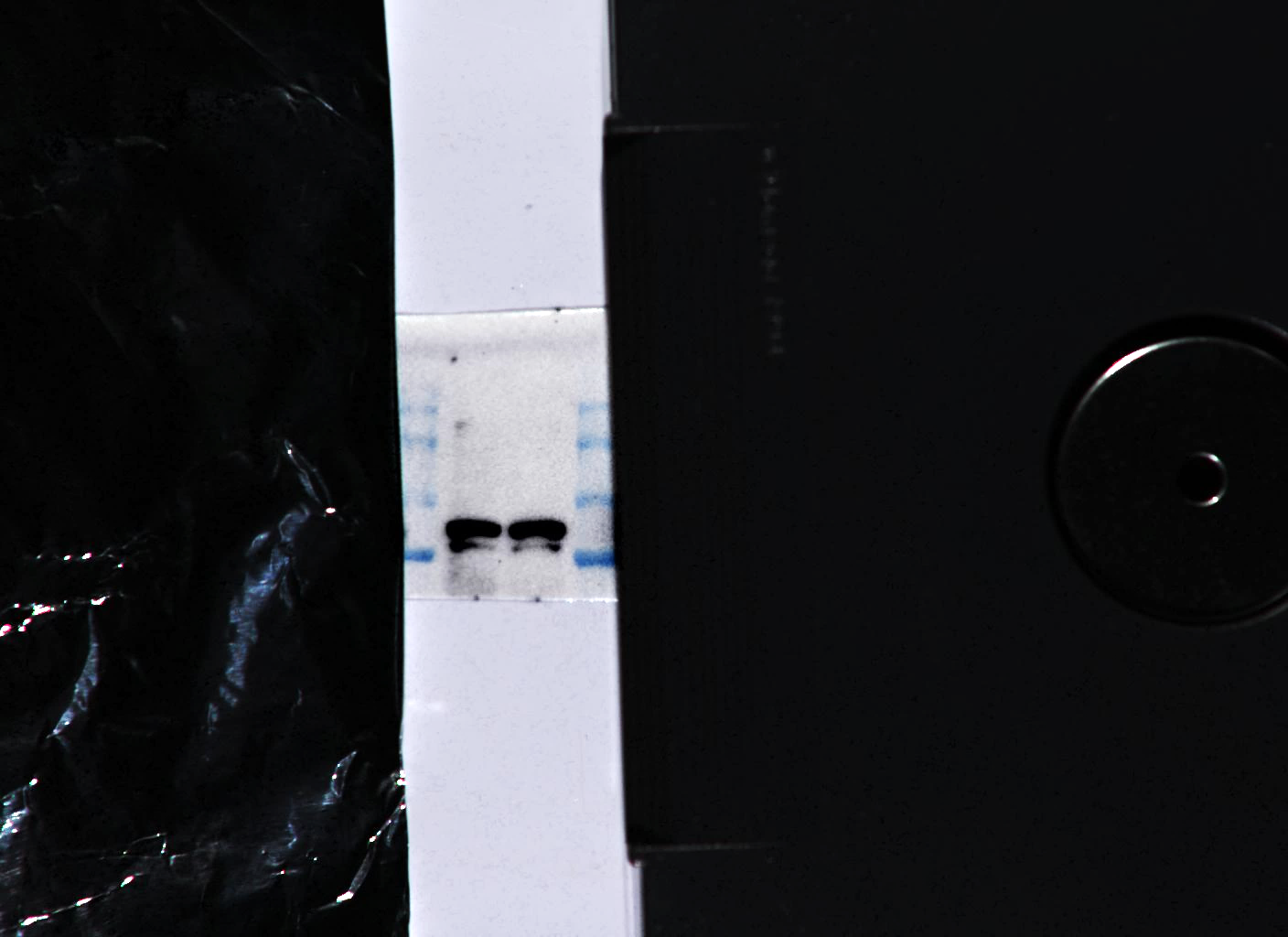
**

**STAT3:**

**
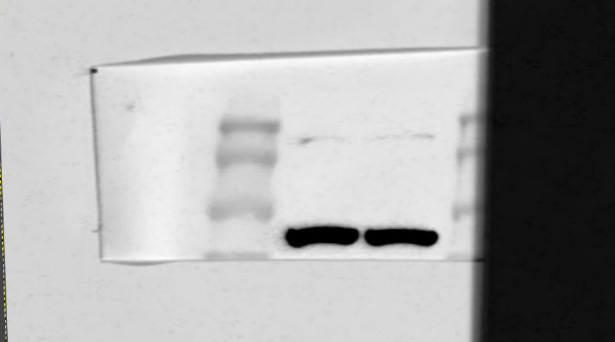

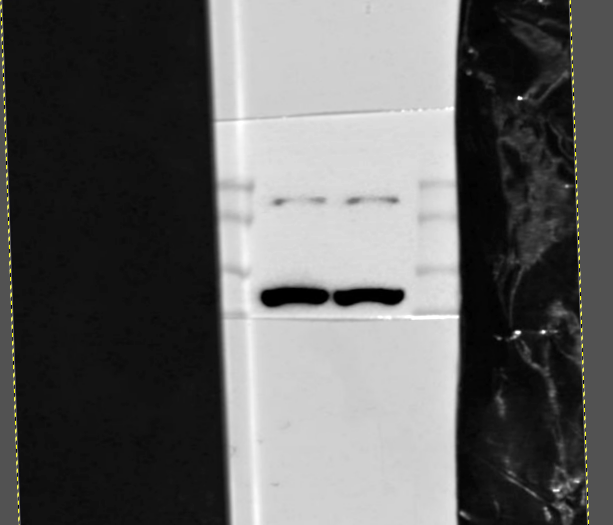
**

**Beta-actina:**


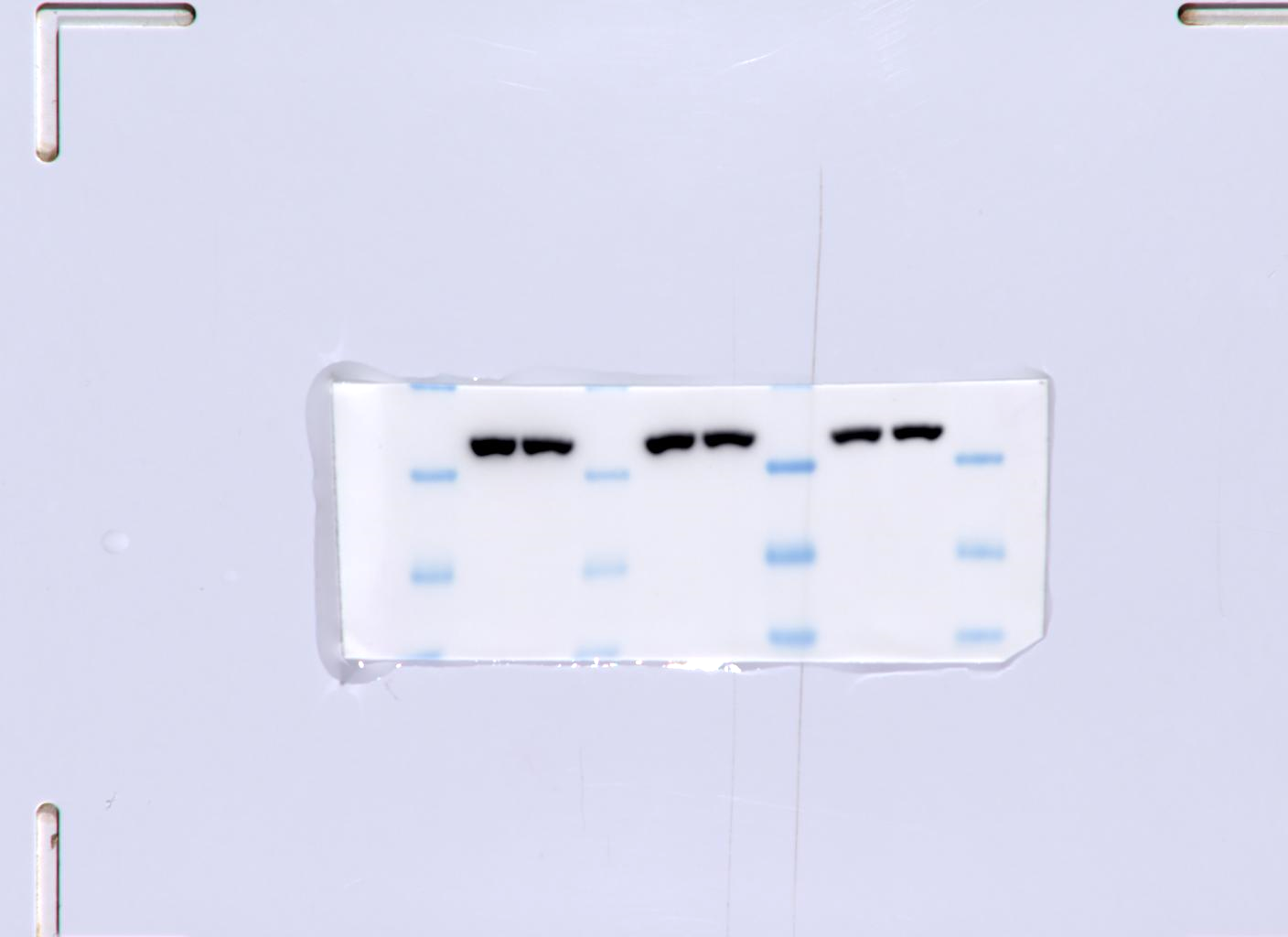

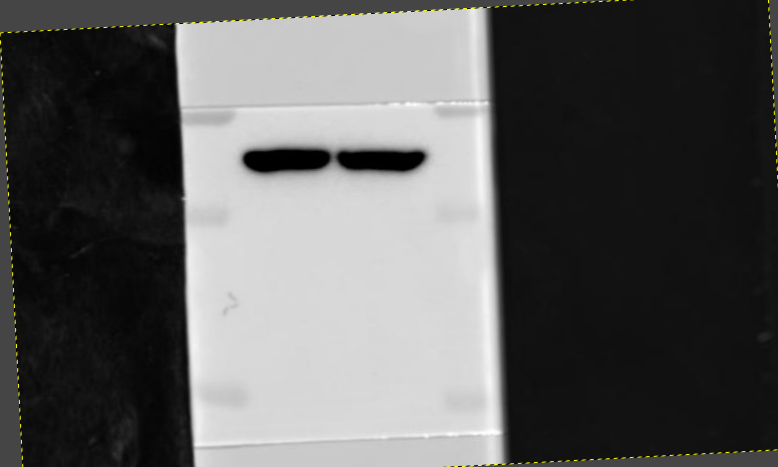


**Figure 4B**

**BT-474**

**S100-A11r**

**no TPD TPD**

**p-STAT3:**

**
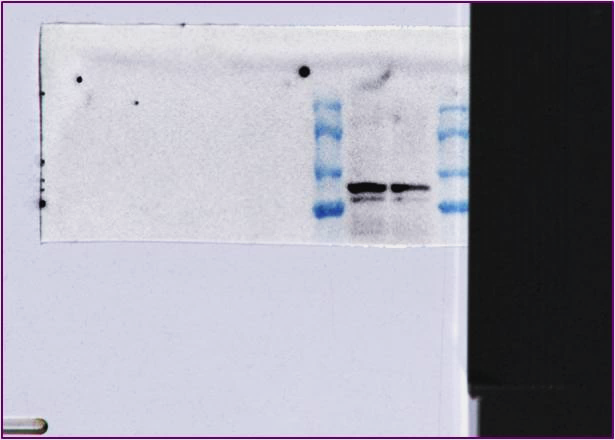

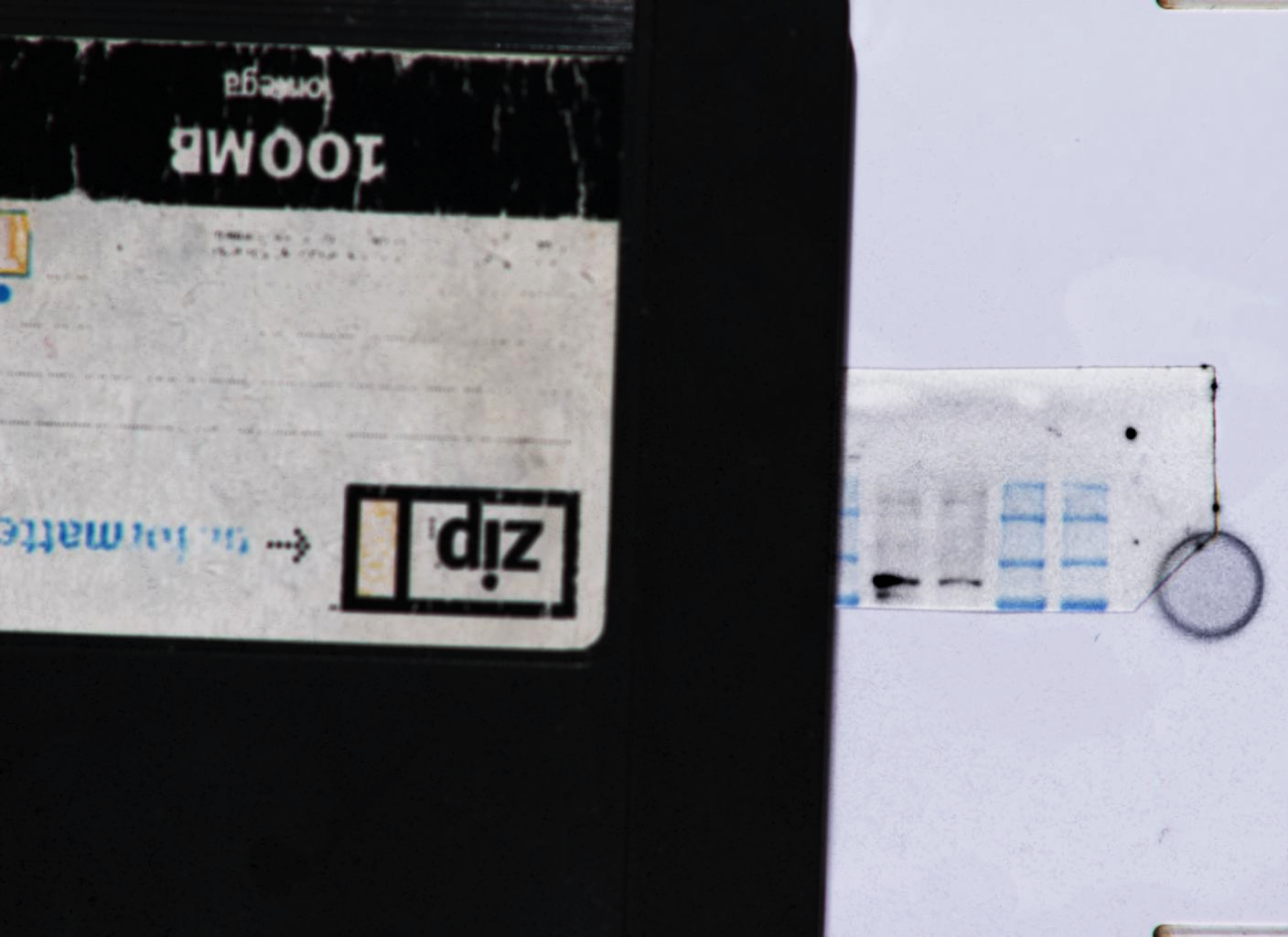
**

**STAT3:**

**
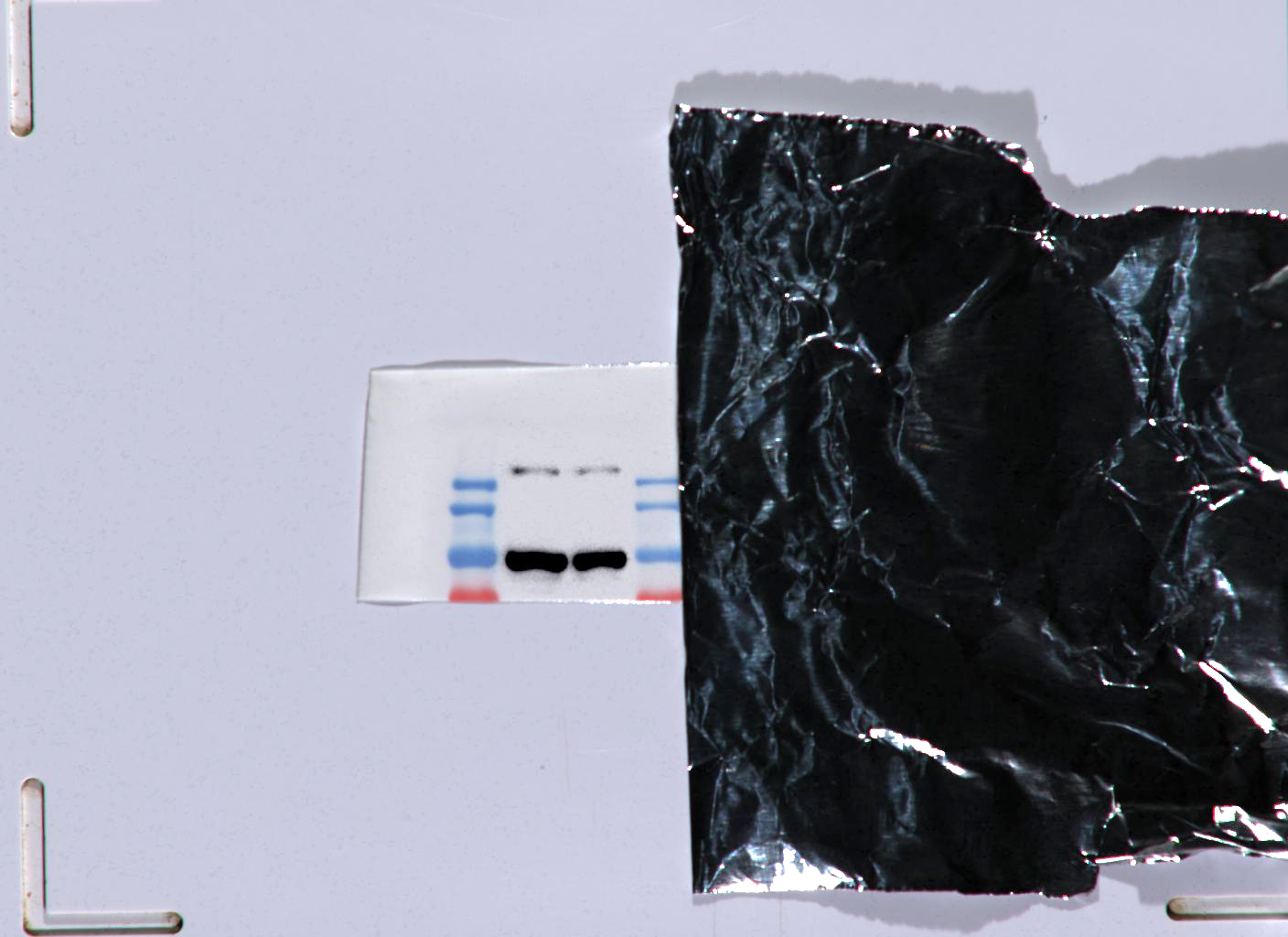

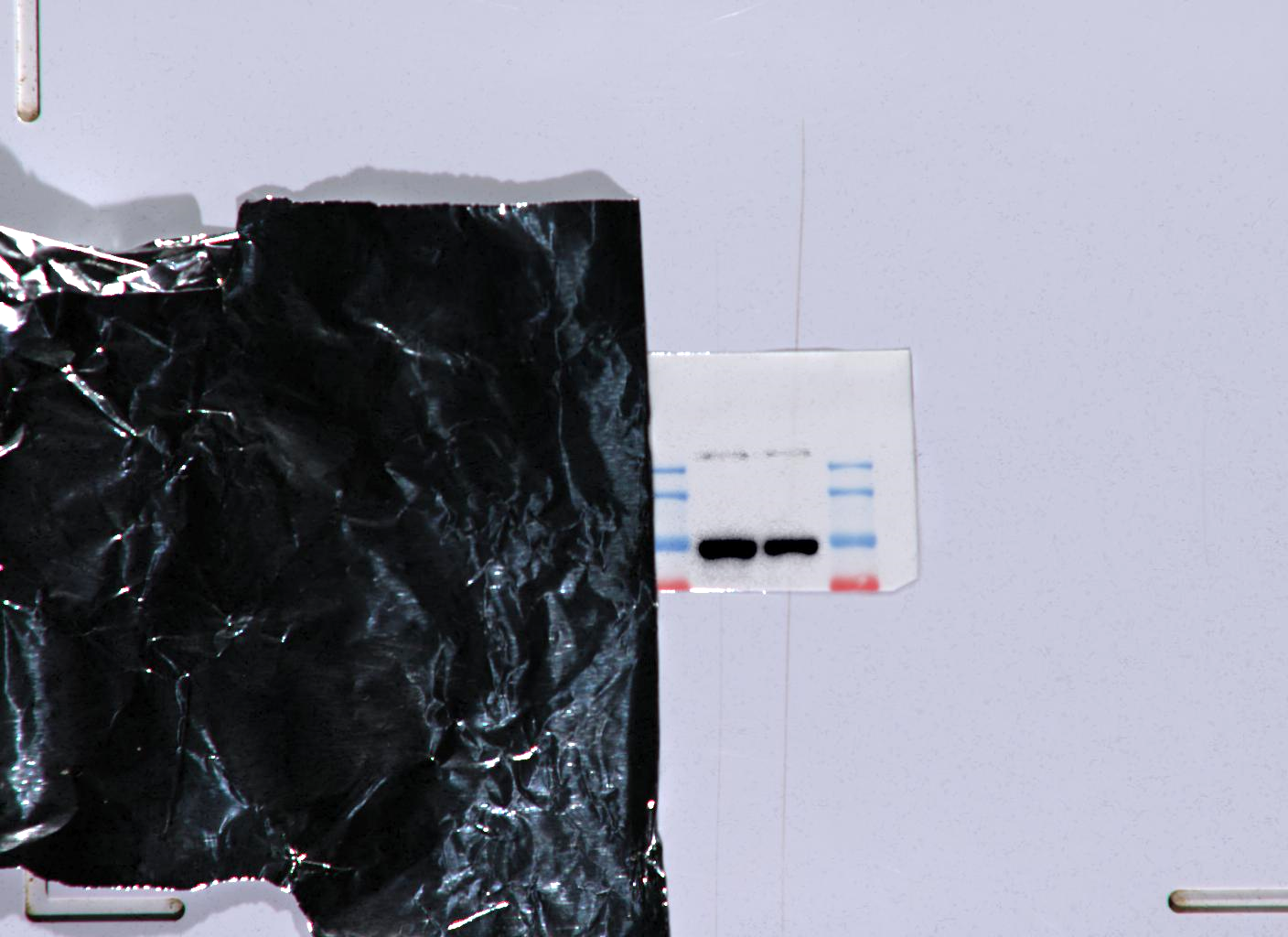
**

**Beta-actina:**

**
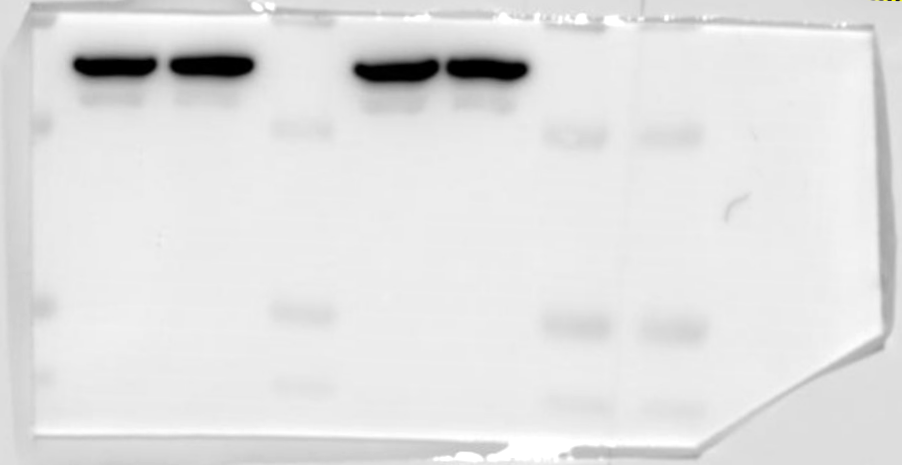

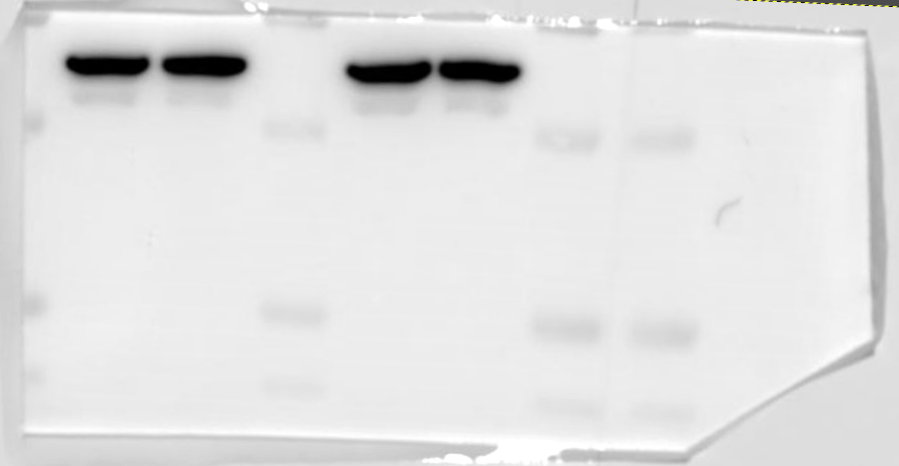
**

**Figure 4B**

**EFM-192A**

**Basal**

**no TPD TPD**

**p-STAT3:**

**
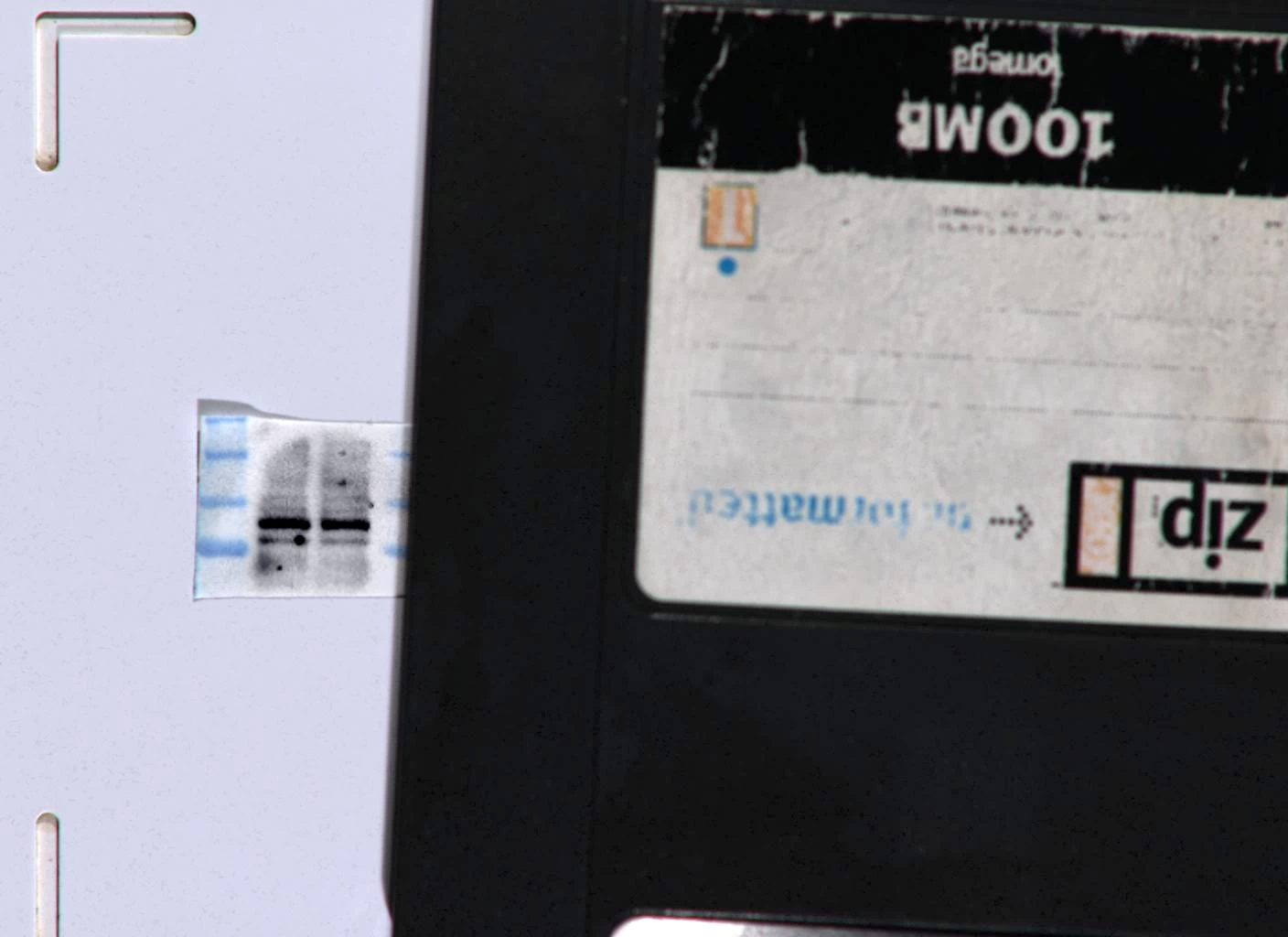

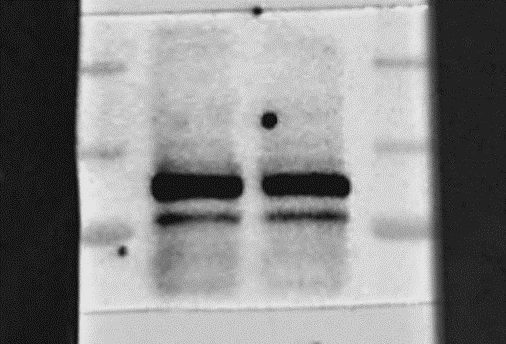
**

**STAT3:**


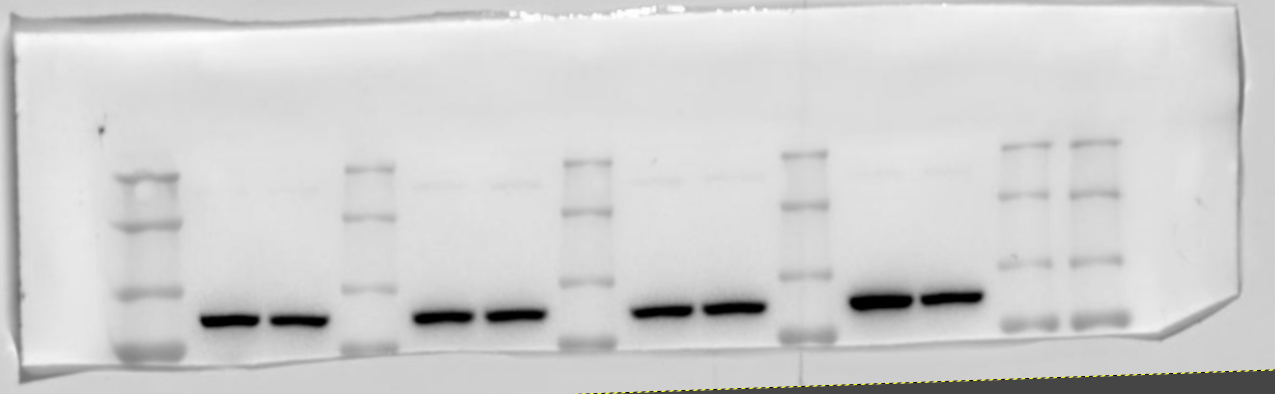

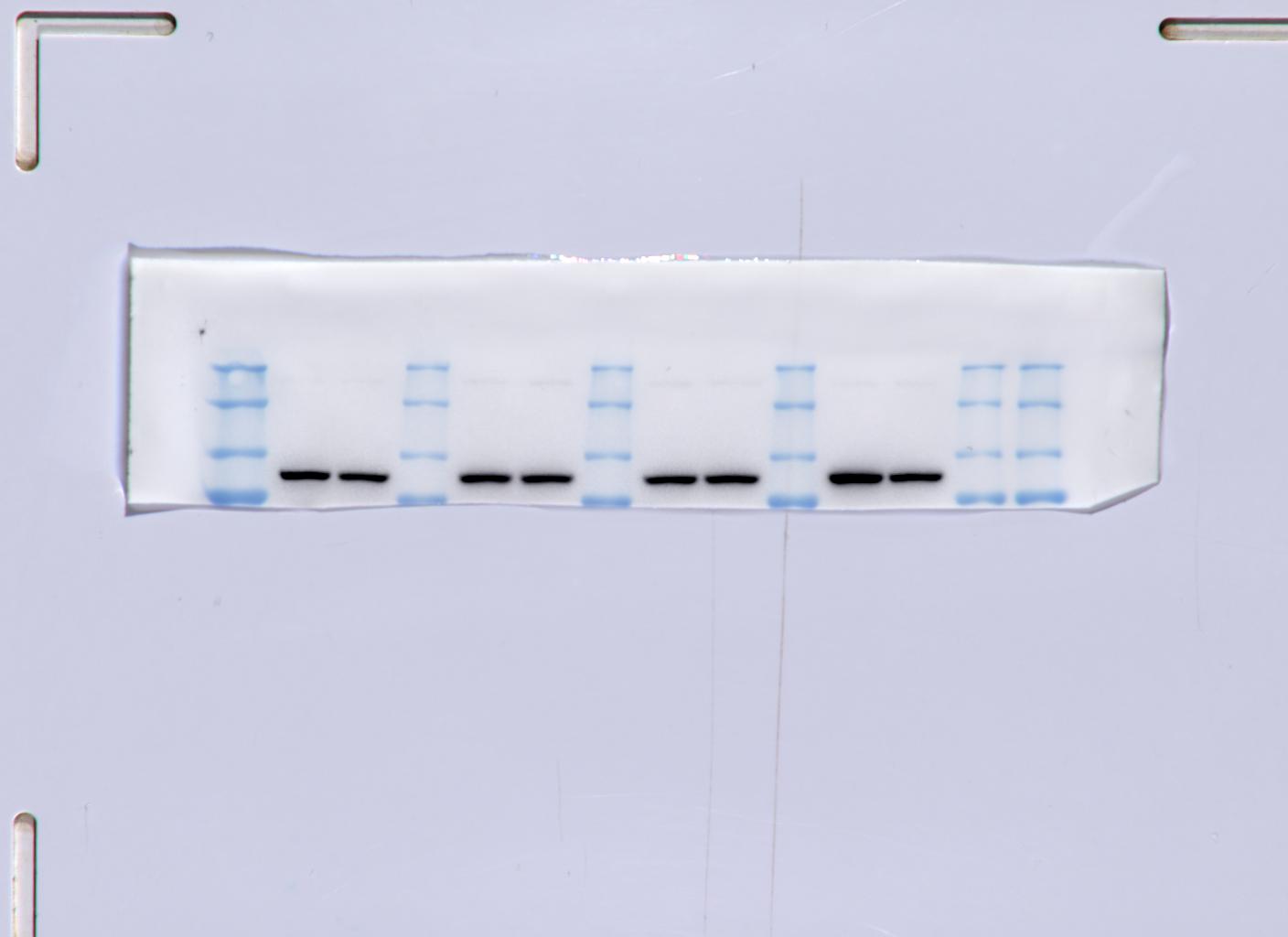


**Beta-actina:**


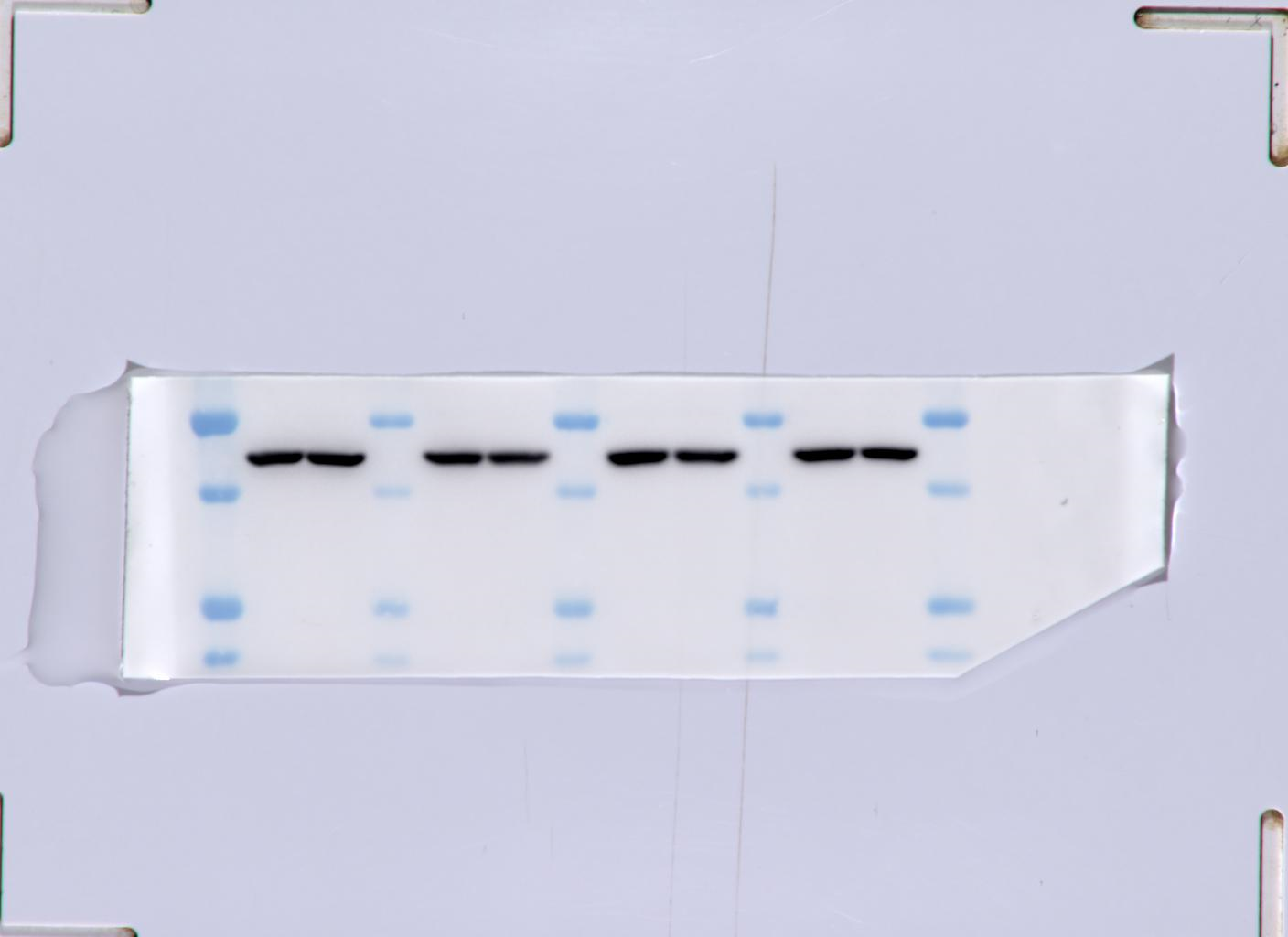

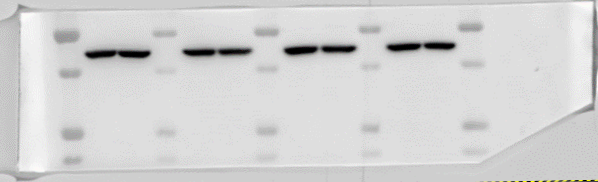


**Figure 4B**

**EFM-192A**

**S100-A11r**

**no TPD TPD**

**p-STAT3:**

**
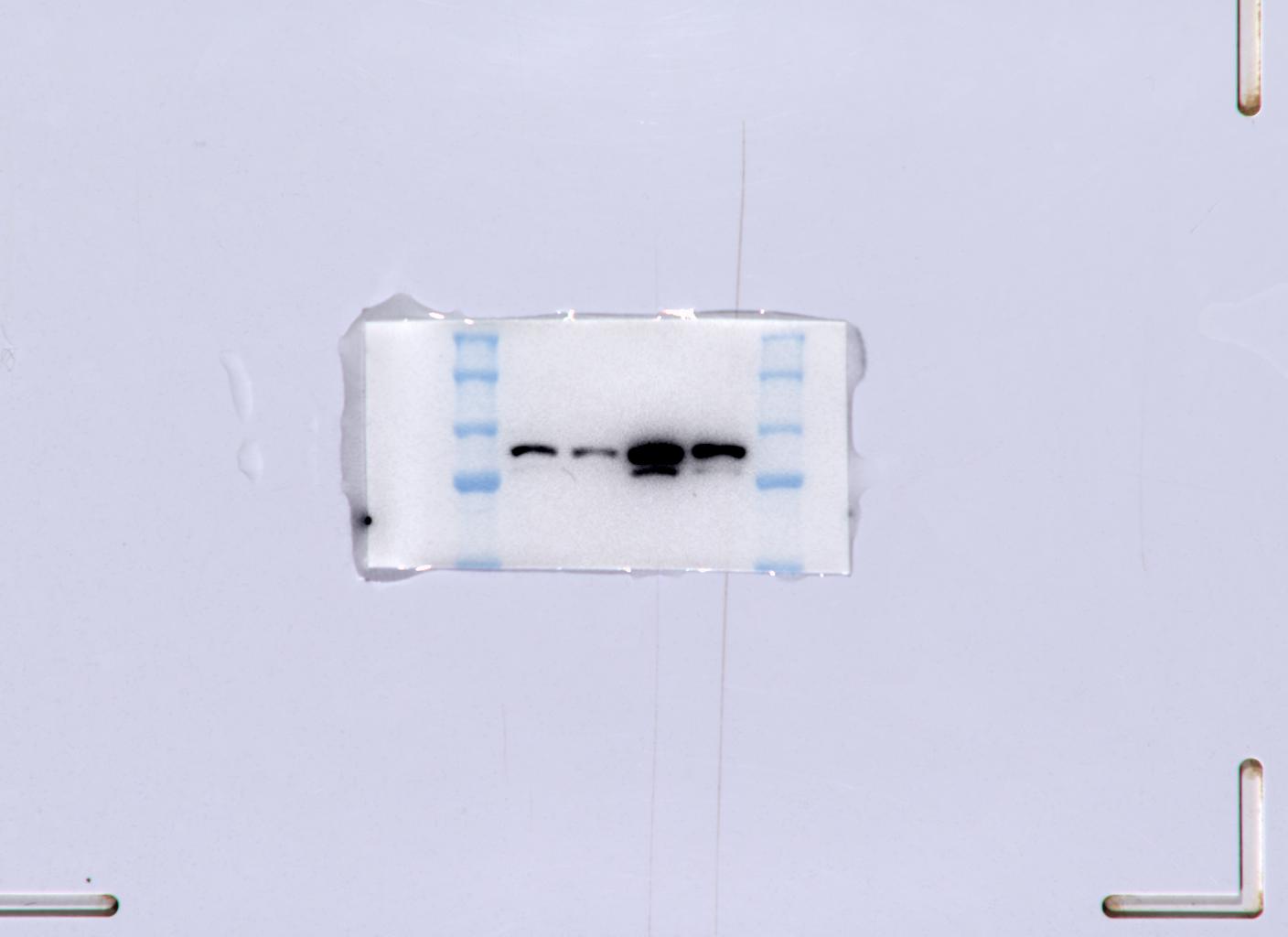

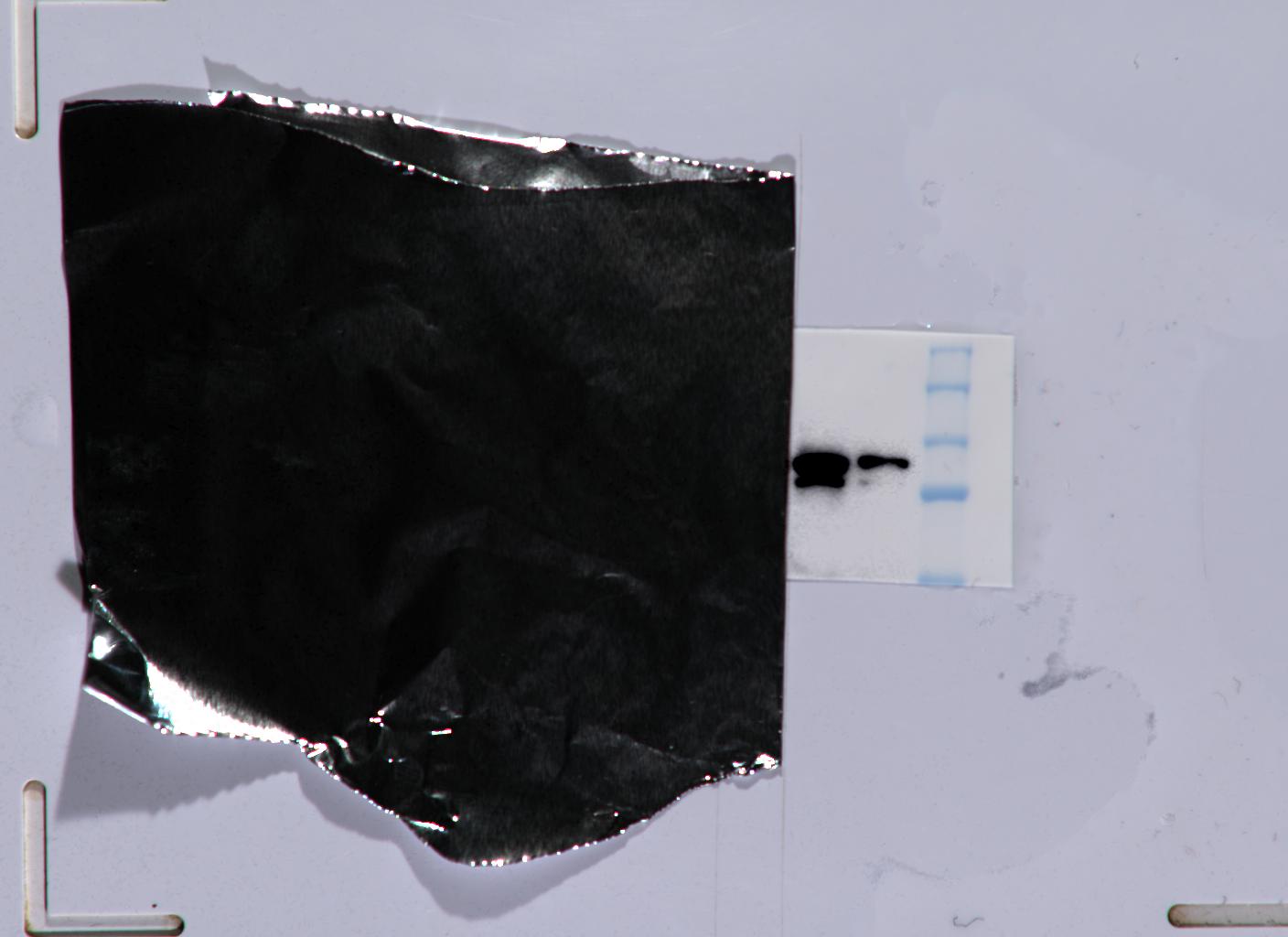
**

**STAT3:**

**
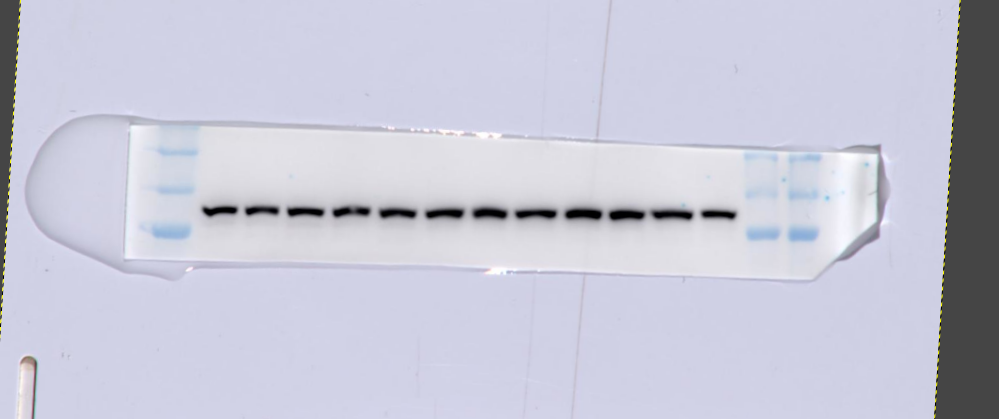

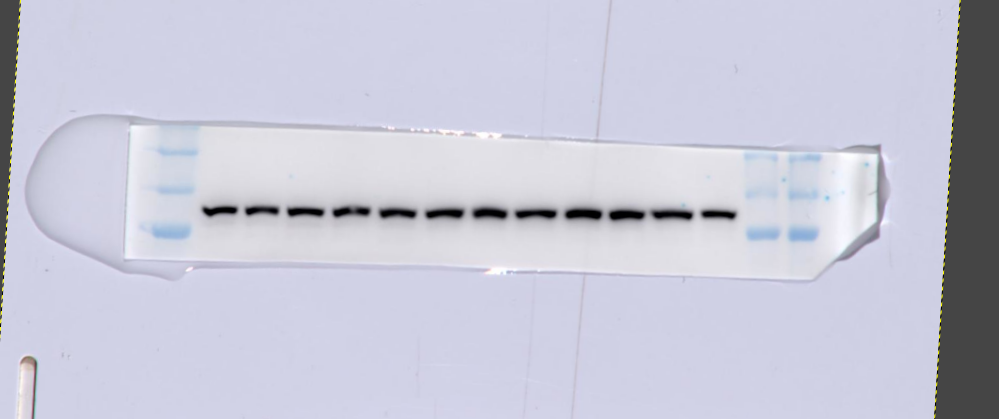
**

**Beta-actina:**

**
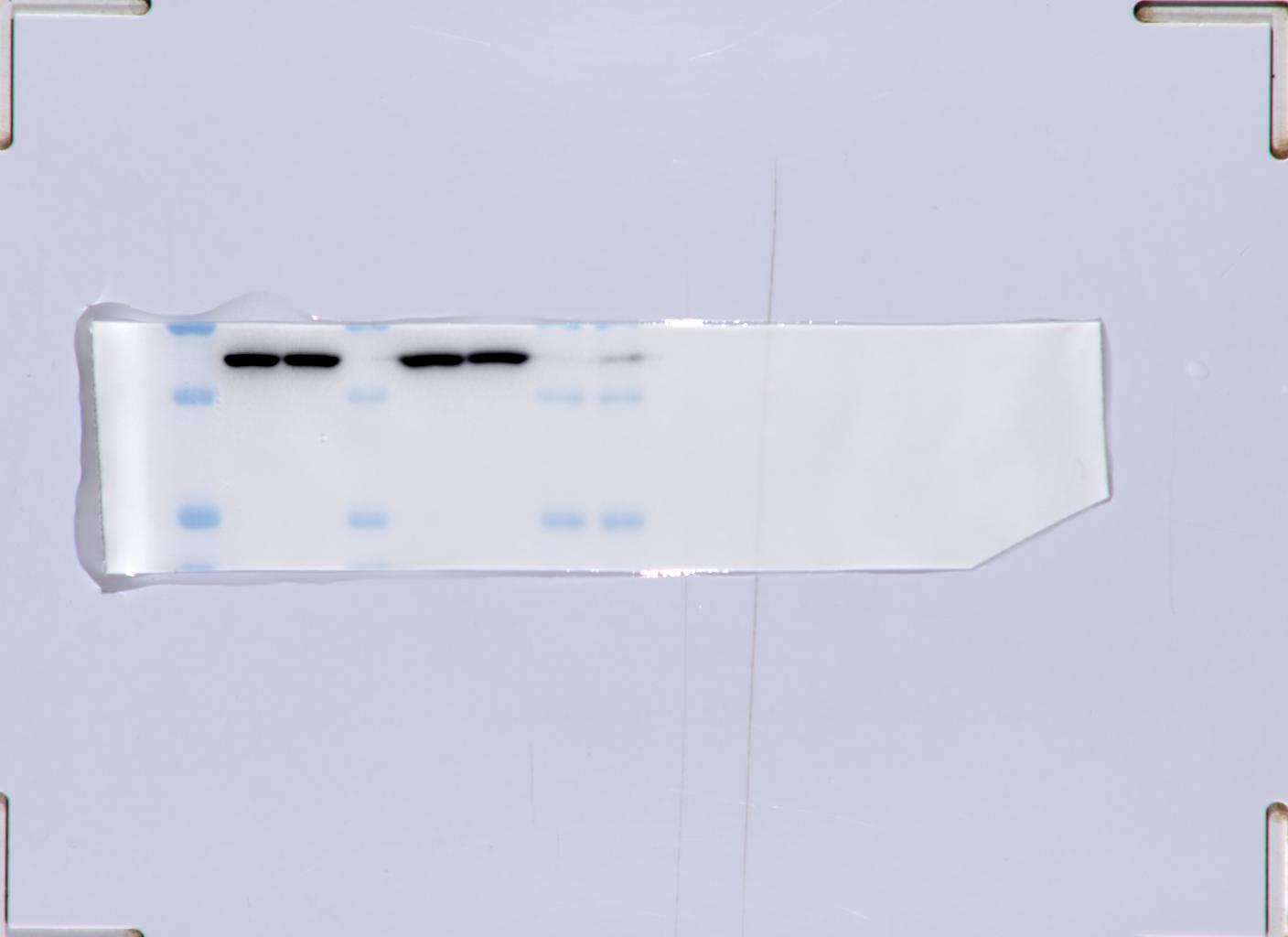
**

**Figure S3**

**BT-474**

**p-STAT3:**

**
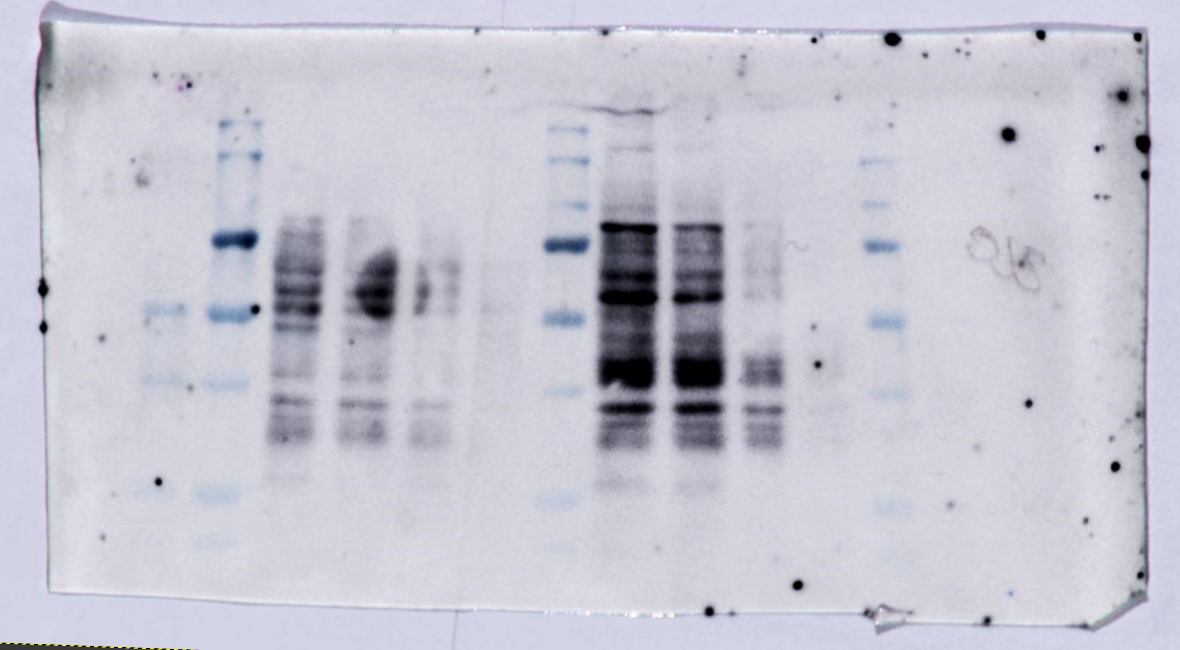
**

**STAT3:**

**
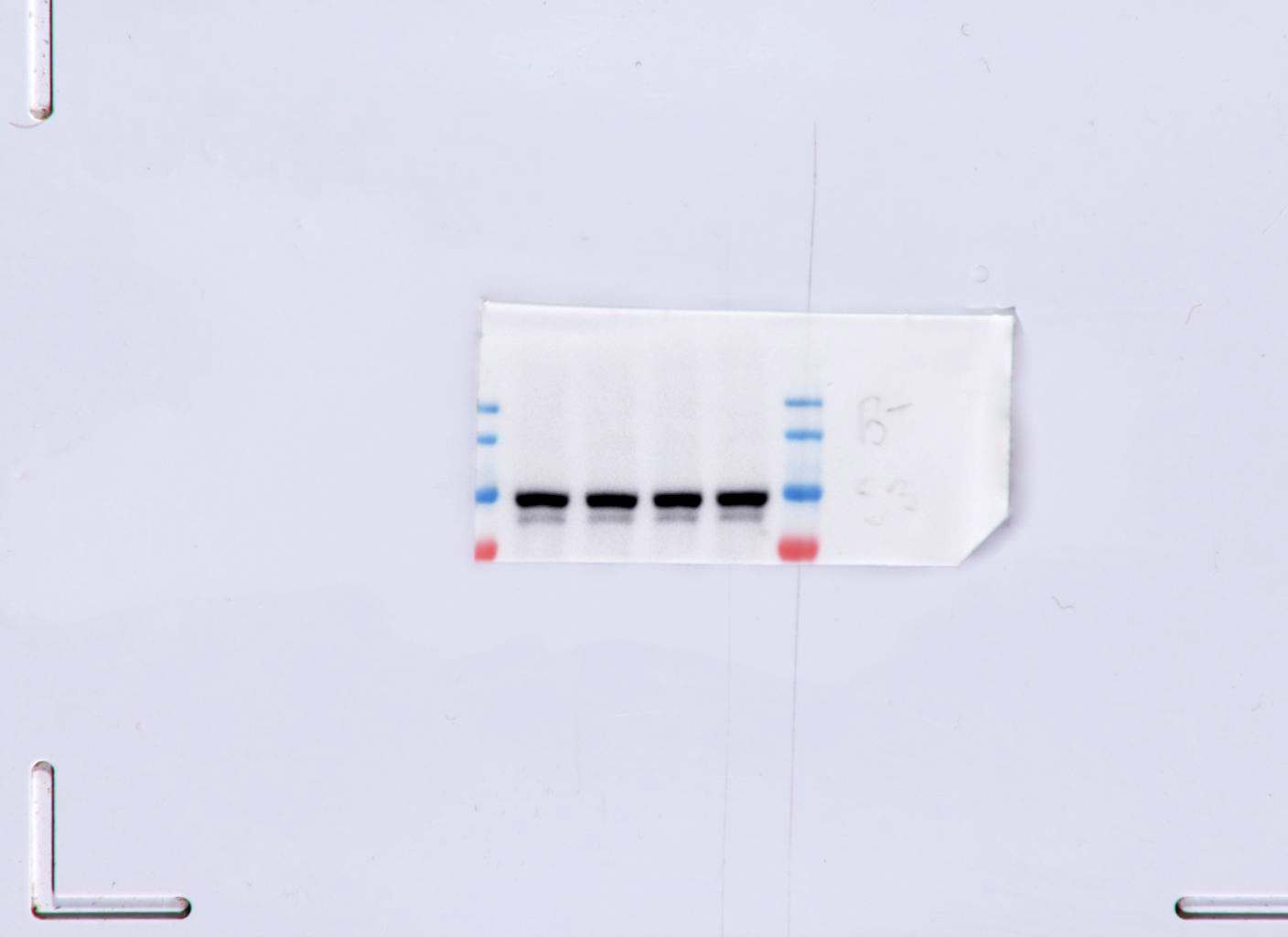
**

**Beta-actina:**

**
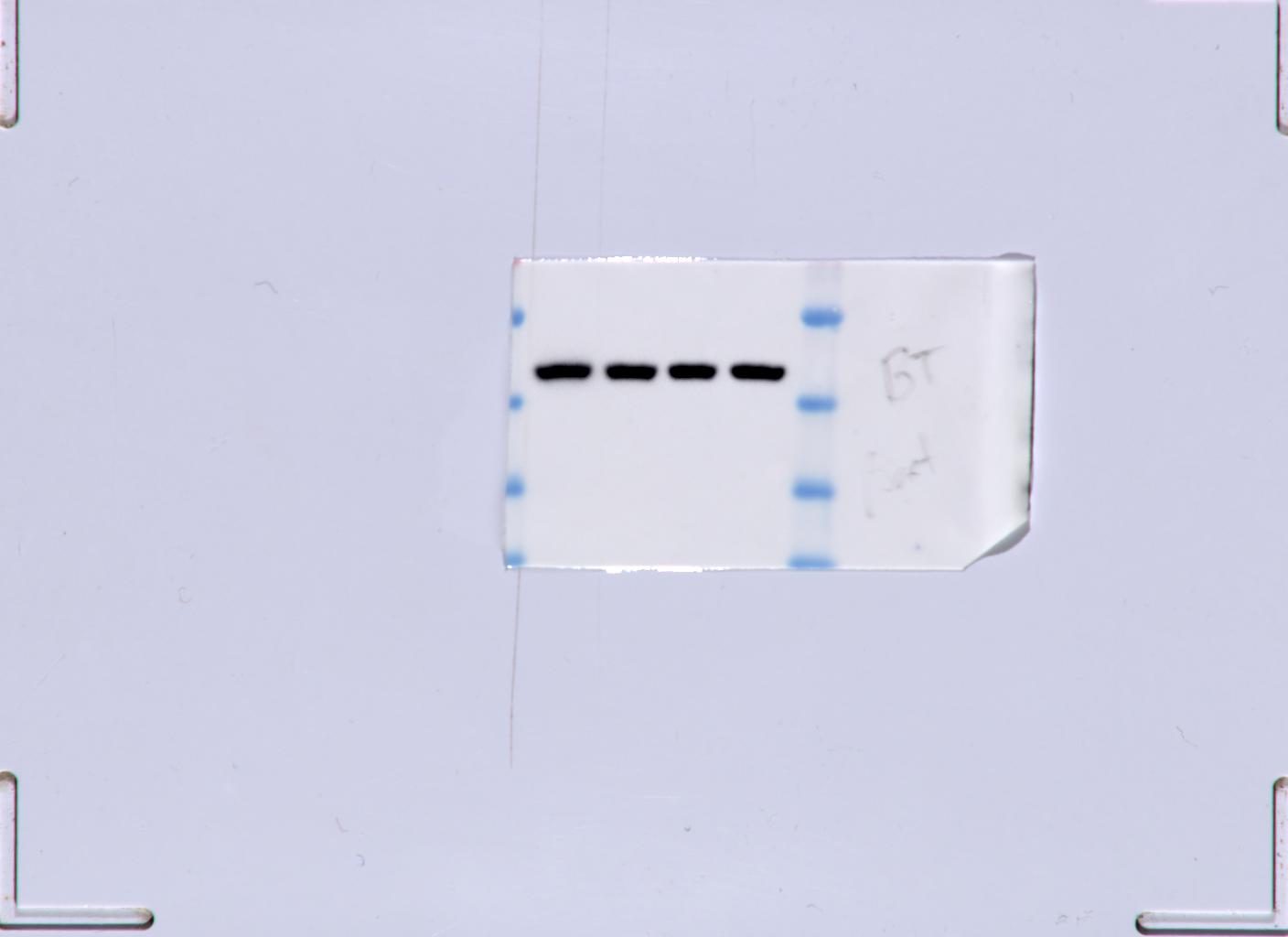
**

**Figure S3**

**EFM-192A**

**p-STAT3:**

**
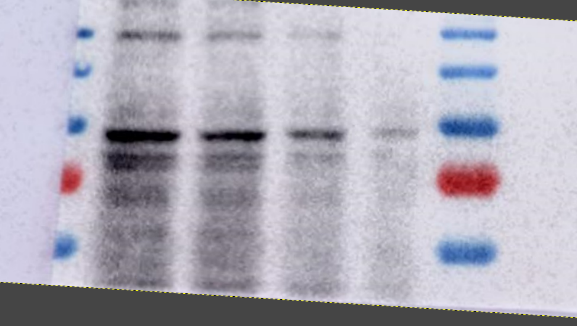
**

**STAT3:**

**
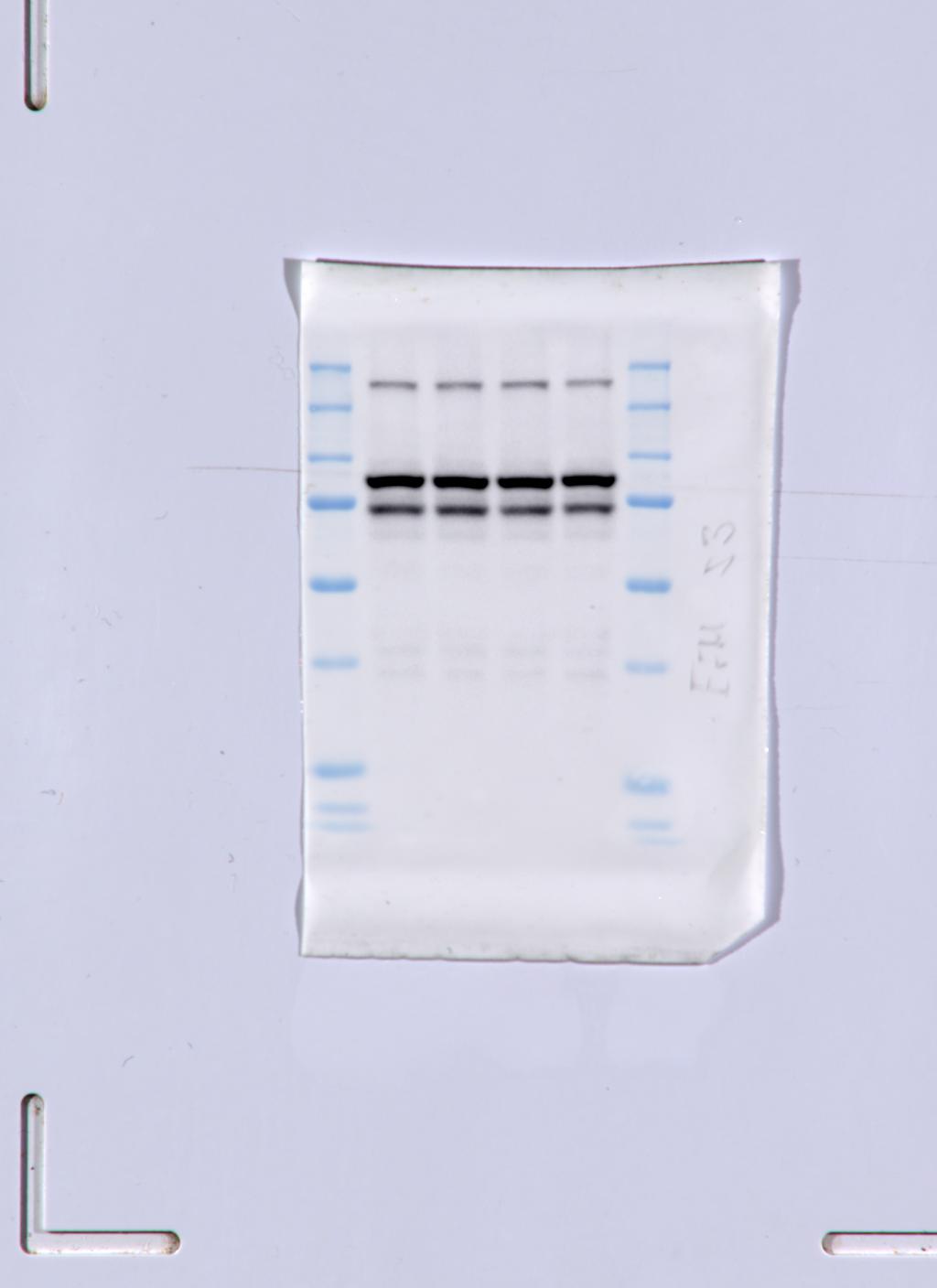
**

**Beta-actina:**

**
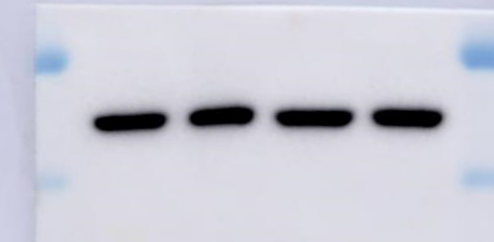
**

**Figure S6**

**AGER/RAGE:**


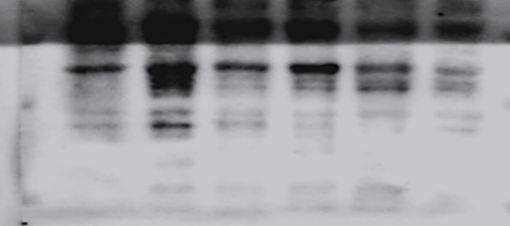


**Beta-actina:**

**
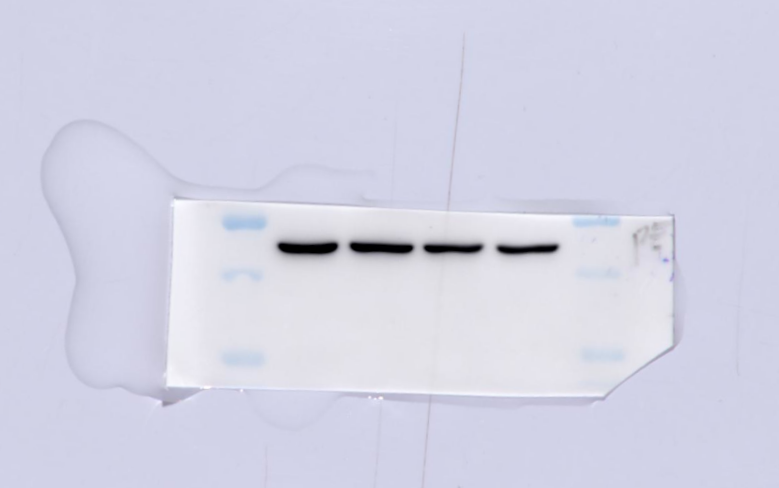
**

**Figure S9**

**BT-474**

**CM[CAF-200]**

**No TPD TPD**

**p-STAT3**


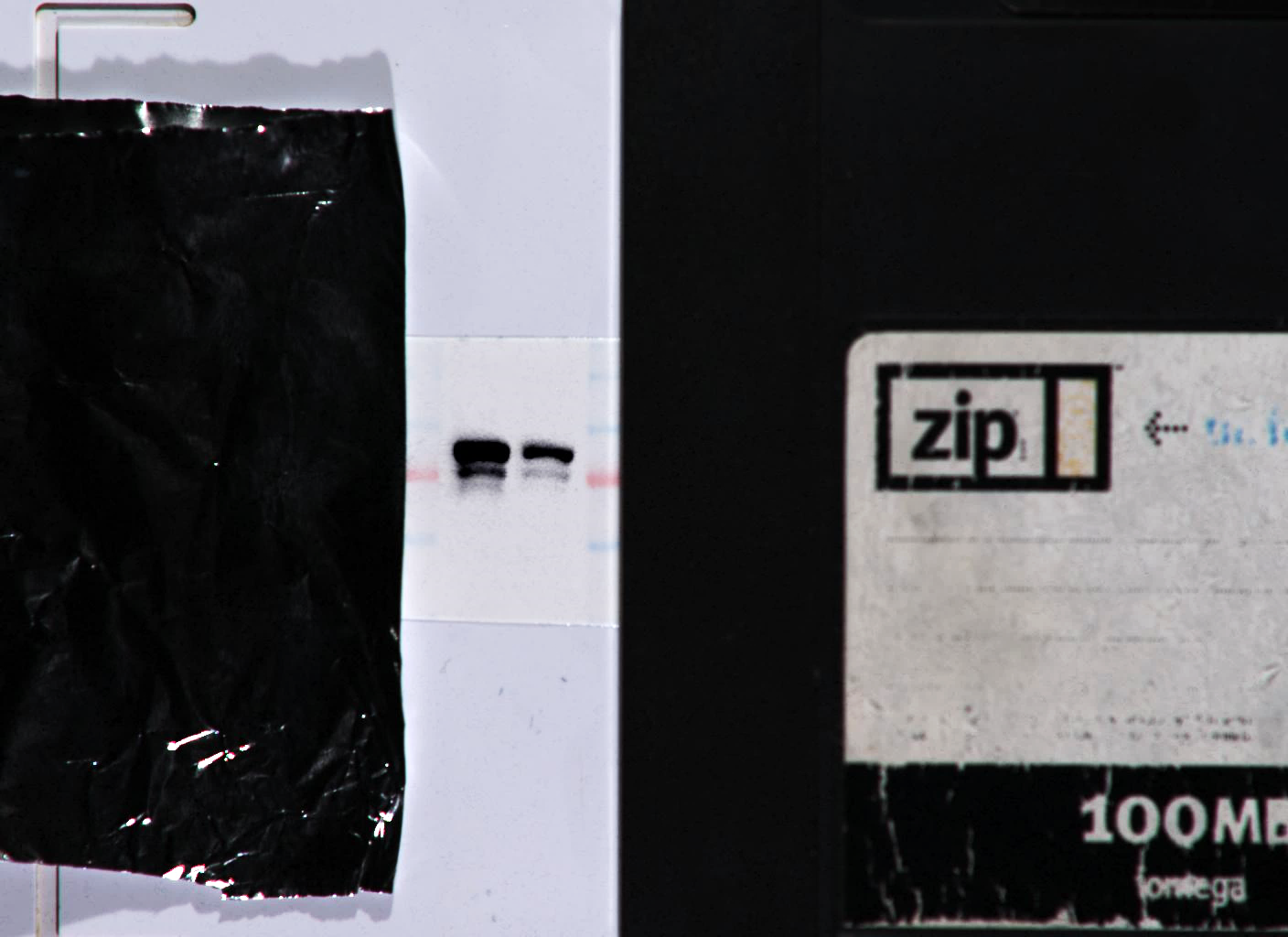

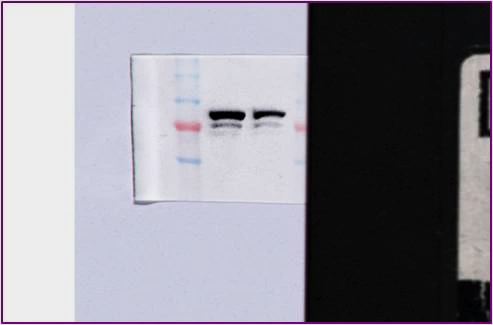


**STAT3:**


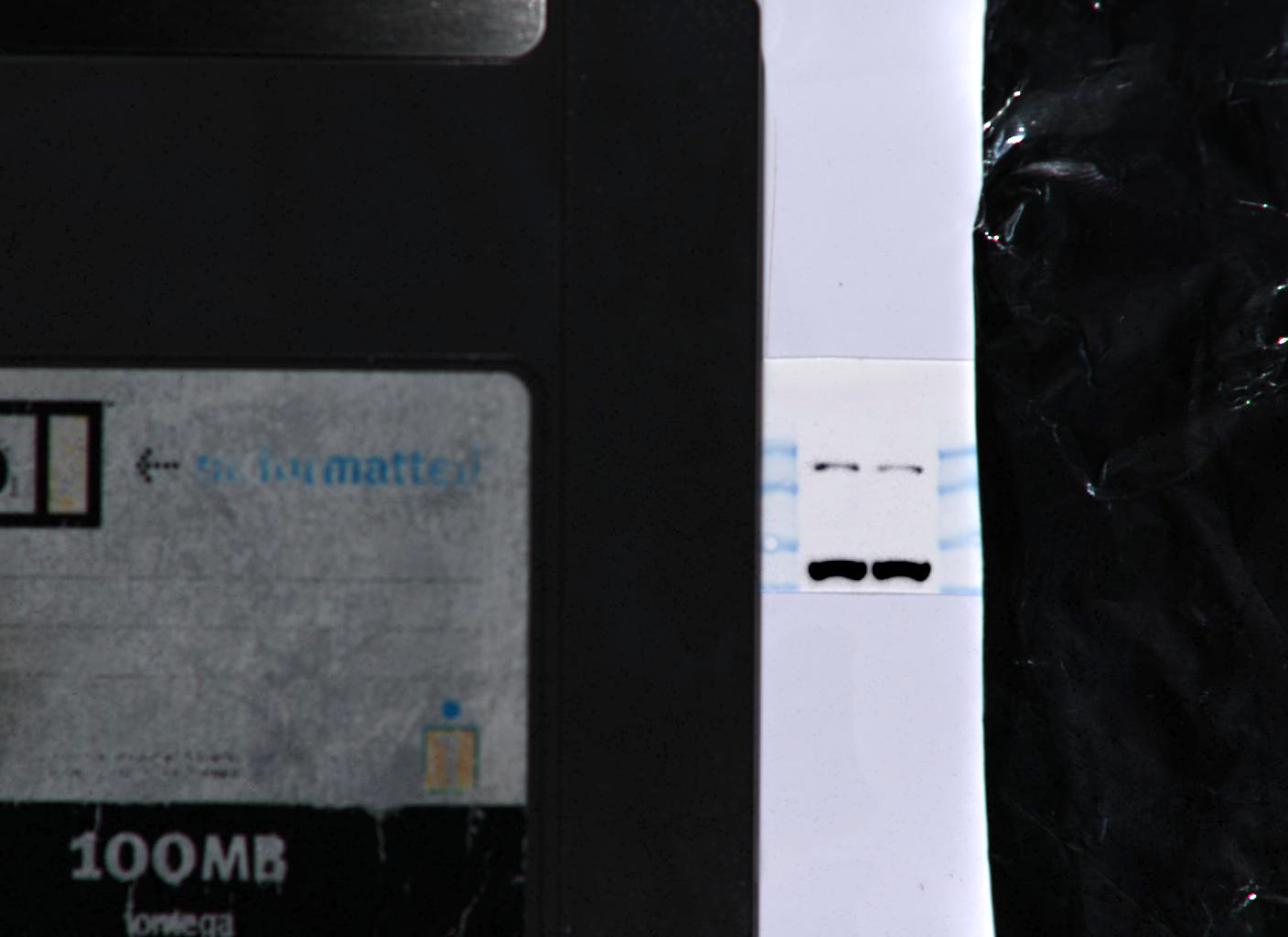

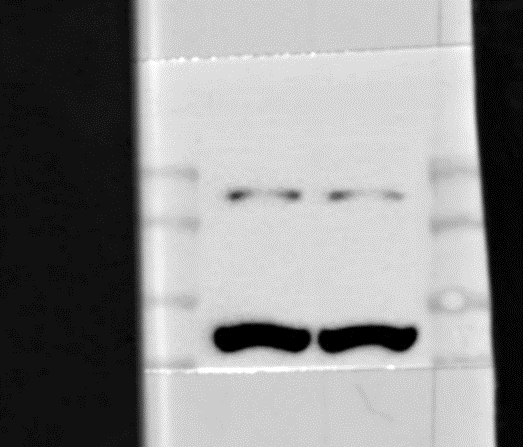


**Beta-actina:**


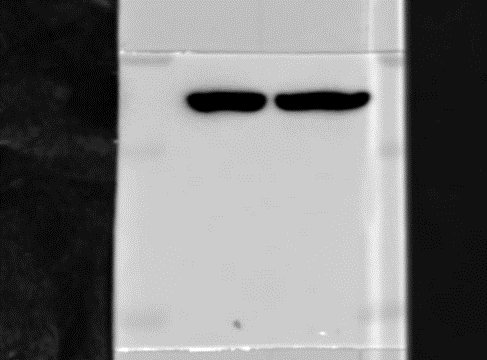
 **
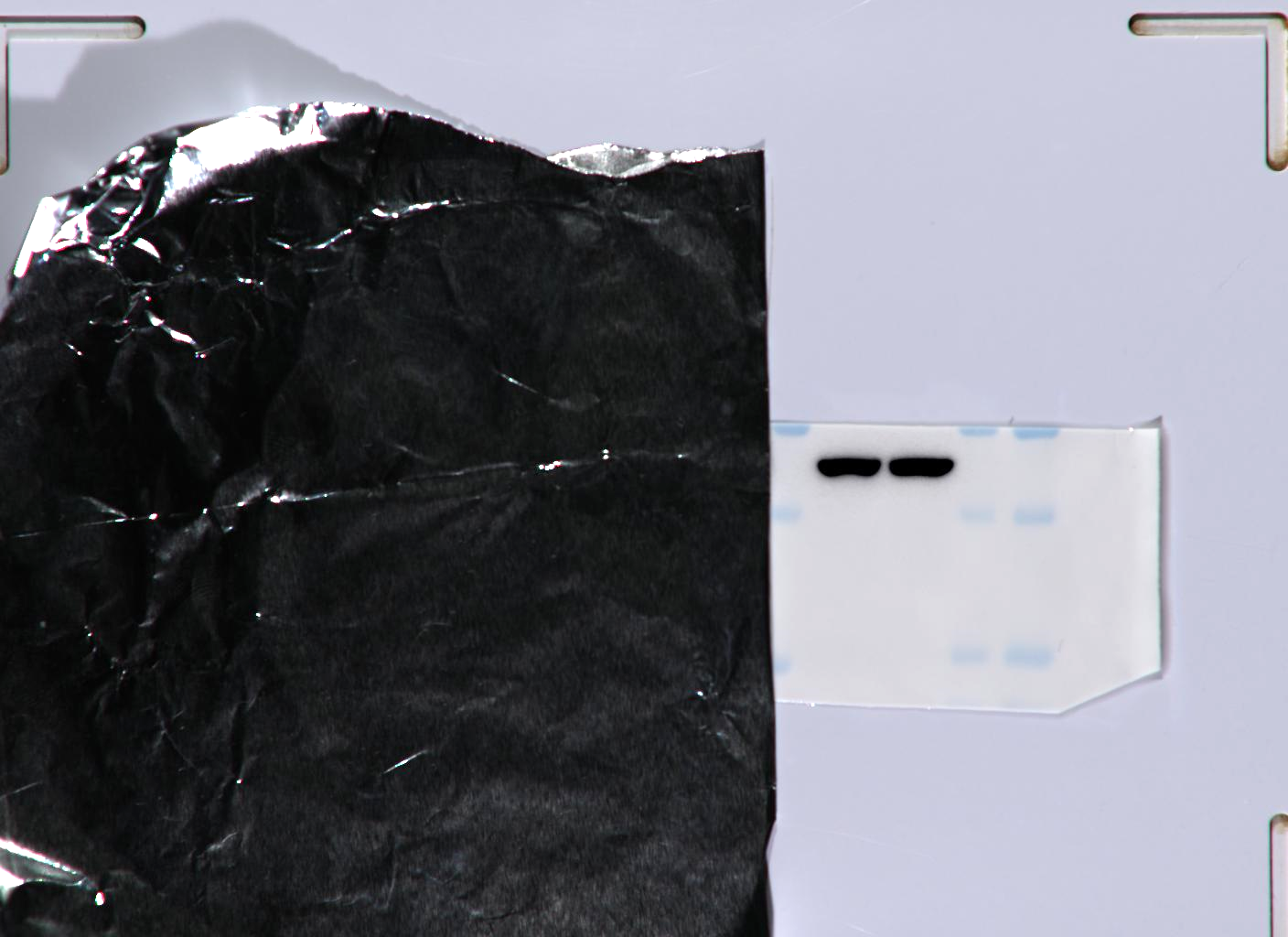
**

**Figure S9**

**EFM-192A**

**CM[CAF-200]**

**No TPD TPD**

**p-STAT3:**


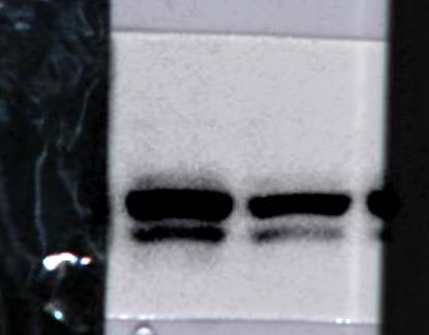

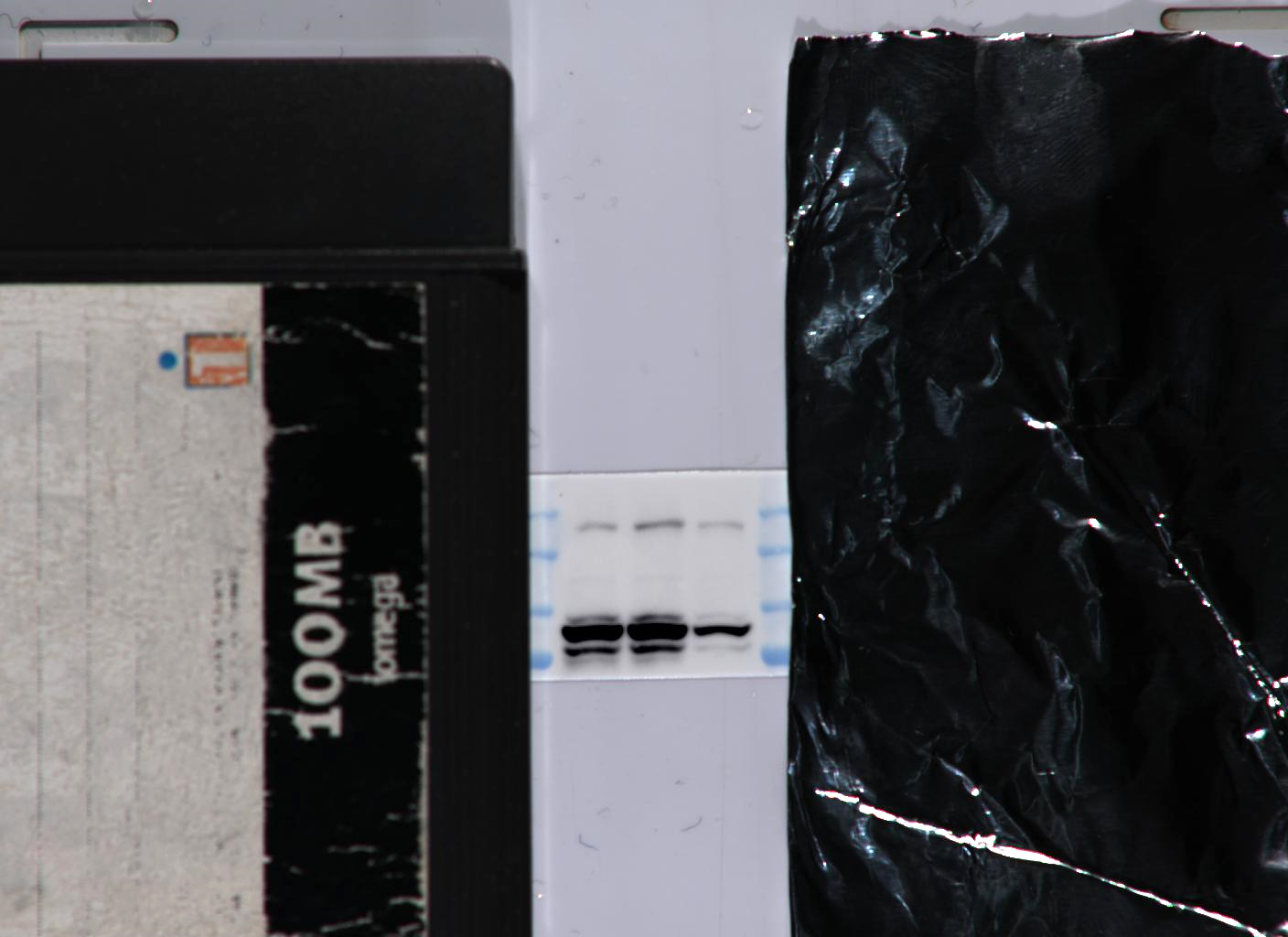


**STAT3:**


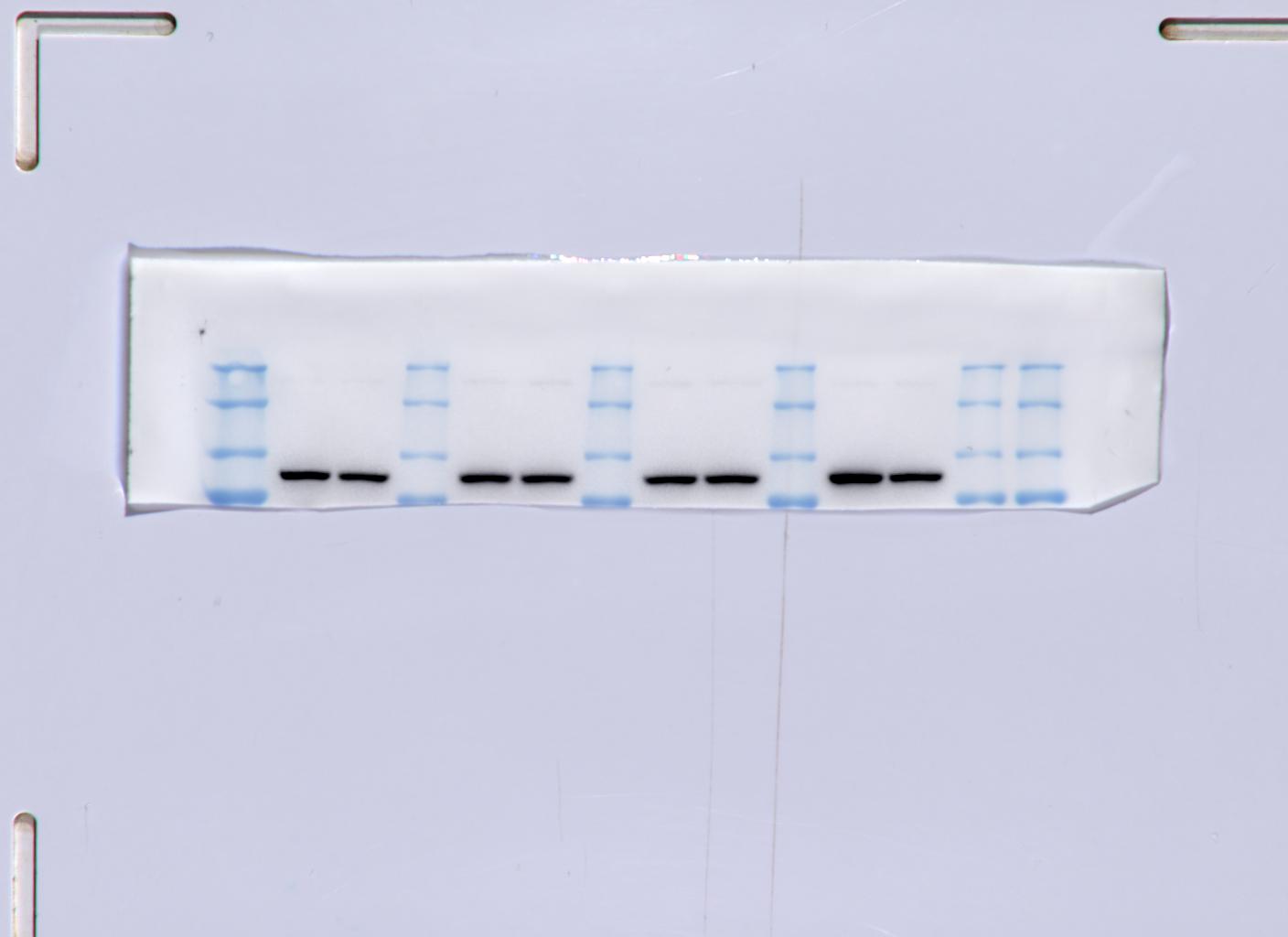

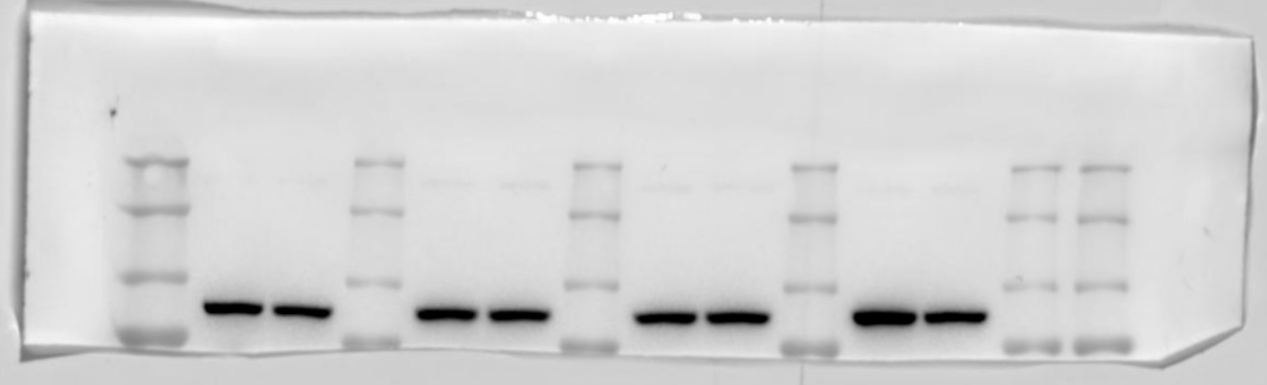


**Beta-actina:**


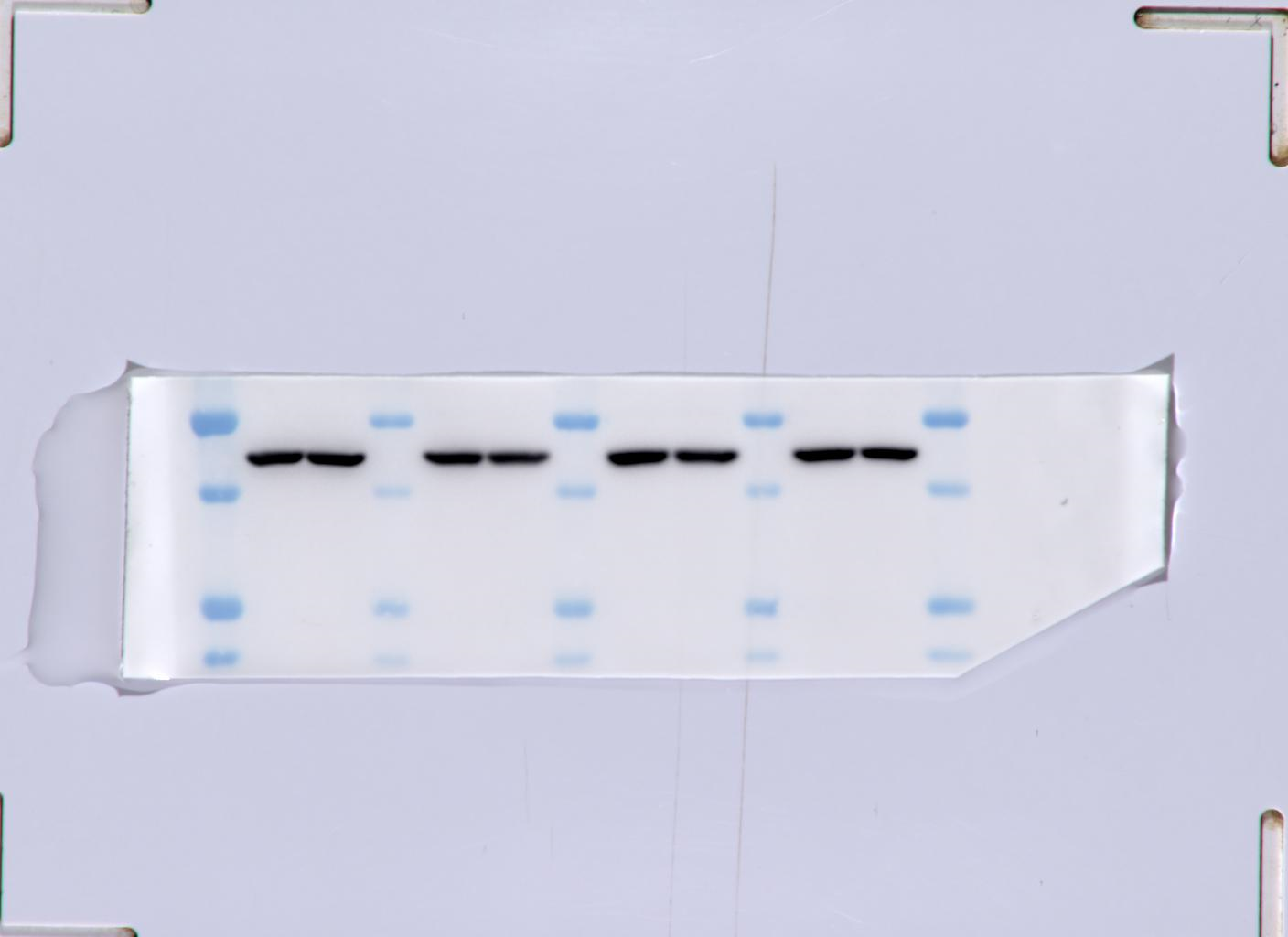

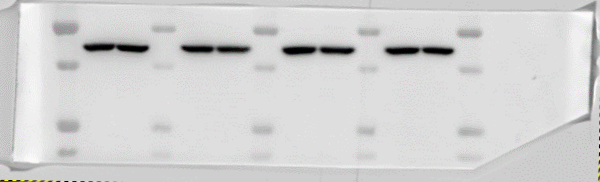


**Figure S10**

**BT-474**

**CM[CAF-200]**

**No TPD (izda) TPD (dcha)**

**p-AKT(Thr308):**

**
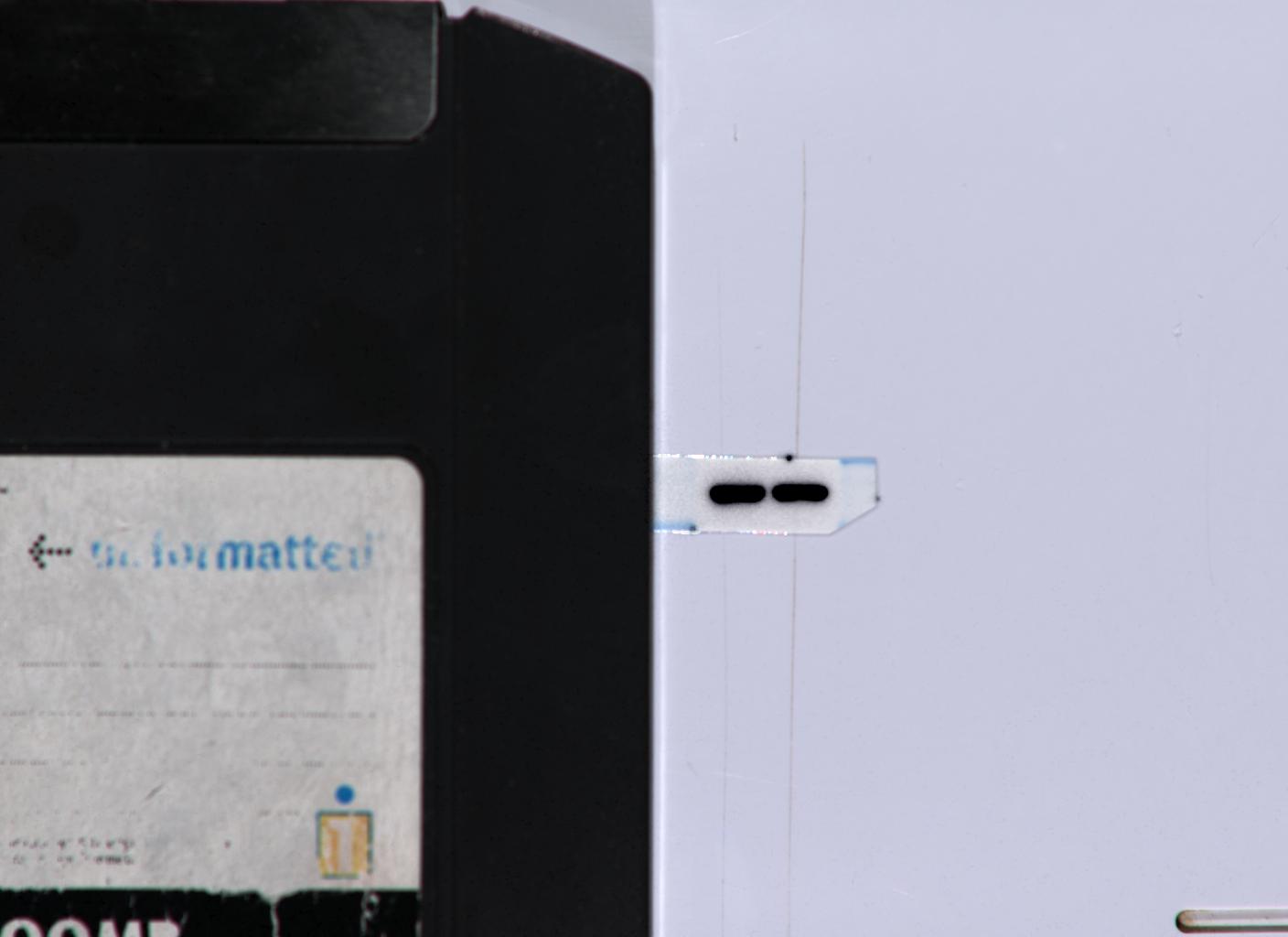

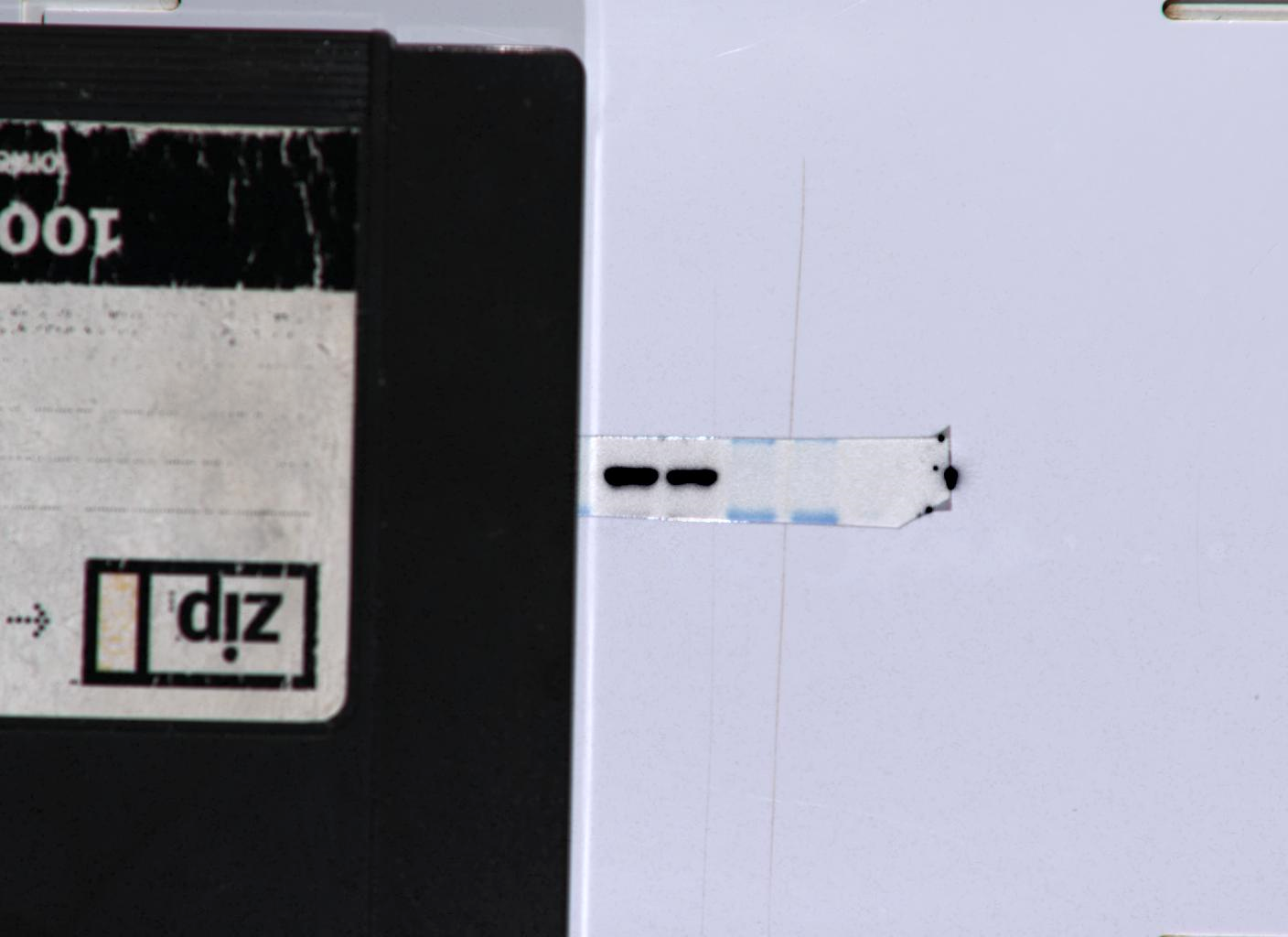
**

**p-AKT(Ser473):**

**
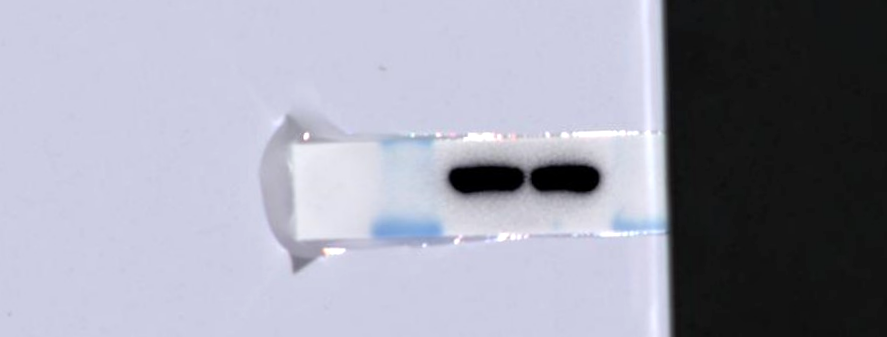

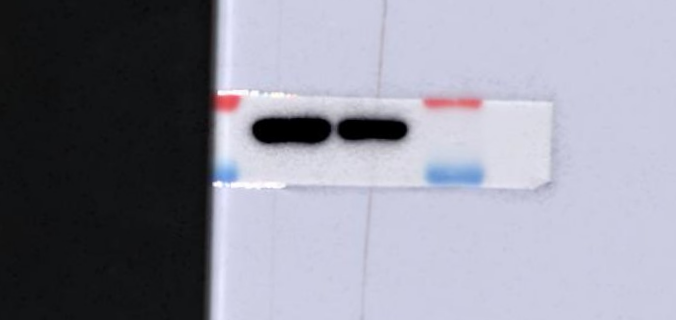
**

**AKT:**

**
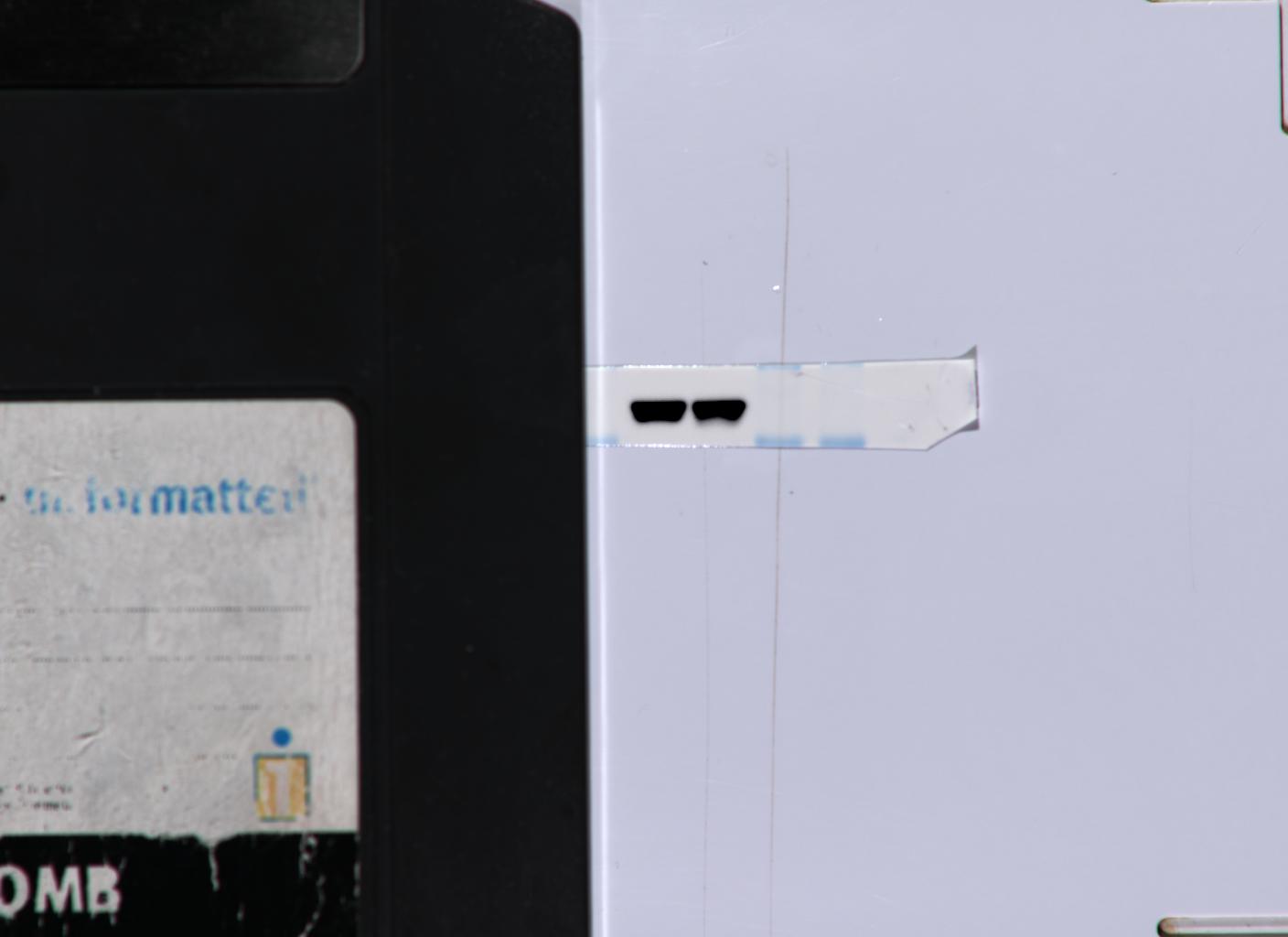

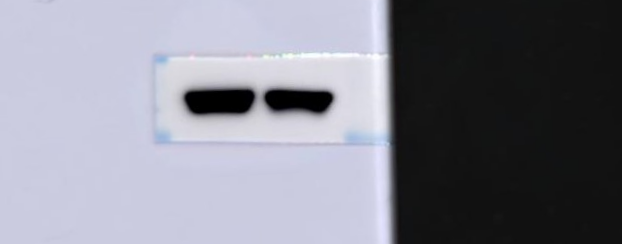
**

**p-ERK:**

**
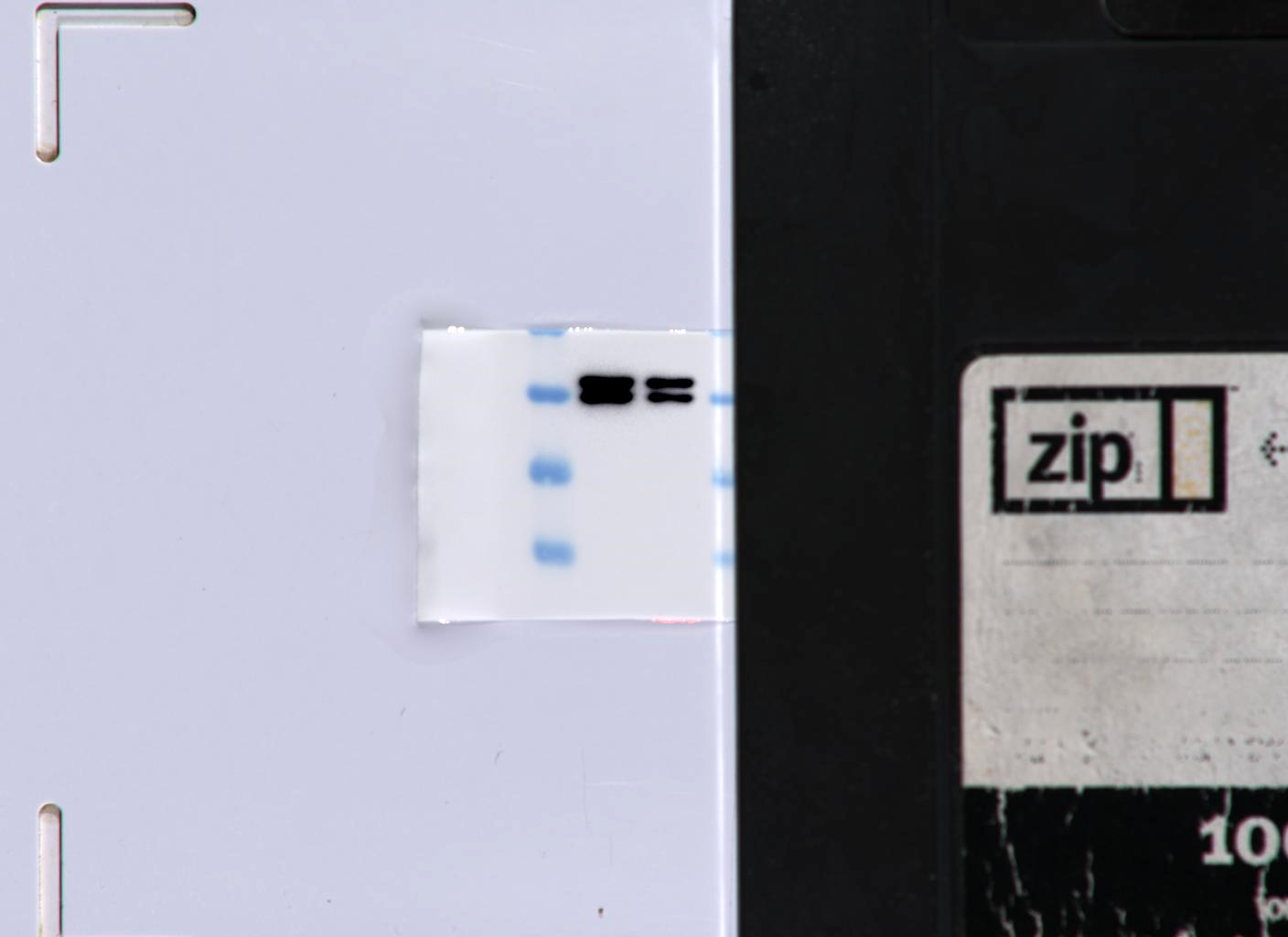

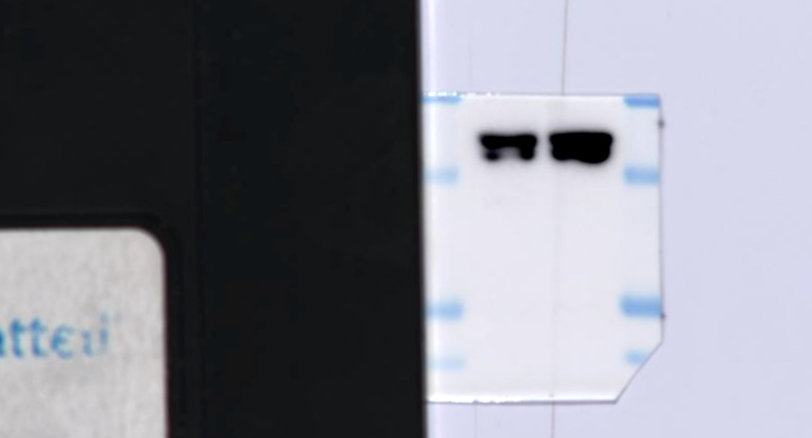
**

**ERK:**

**
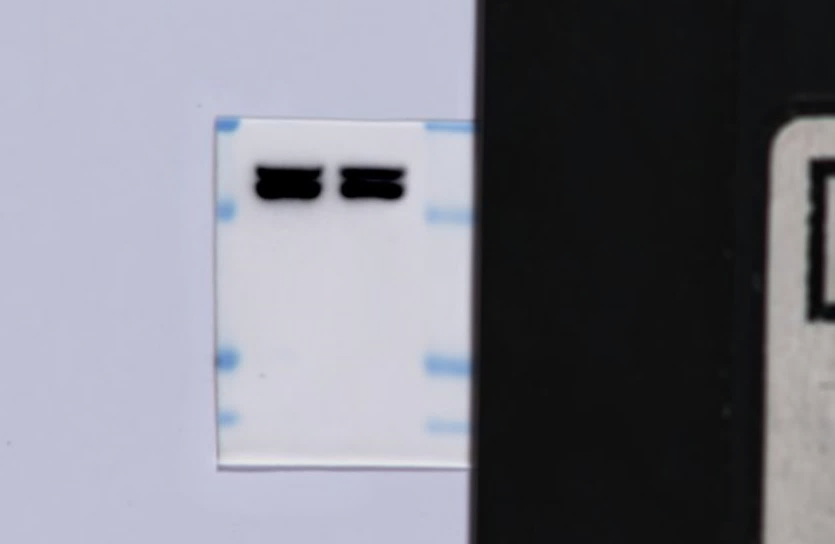

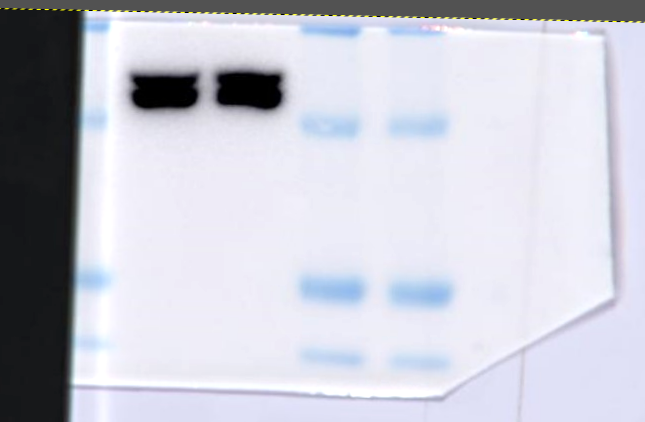
**

**Beta-actina:**

**
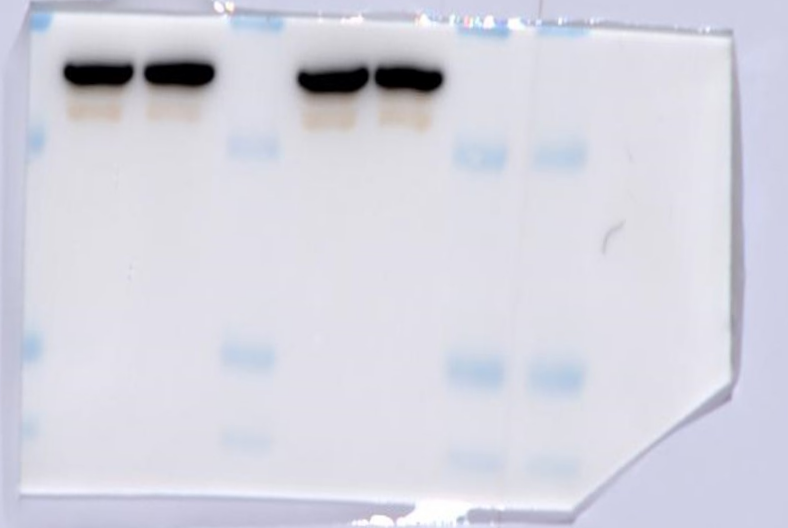
**

**Figure S10**

**EFM-192A**

**CM[CAF-200]**

**No TPD (izda) TPD (dcha)**

**p-AKT(Thr308):**

**
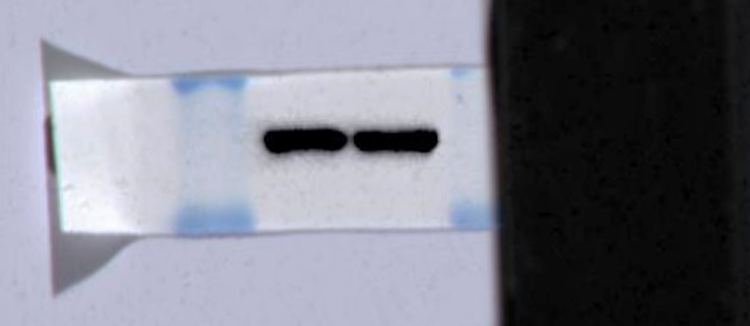

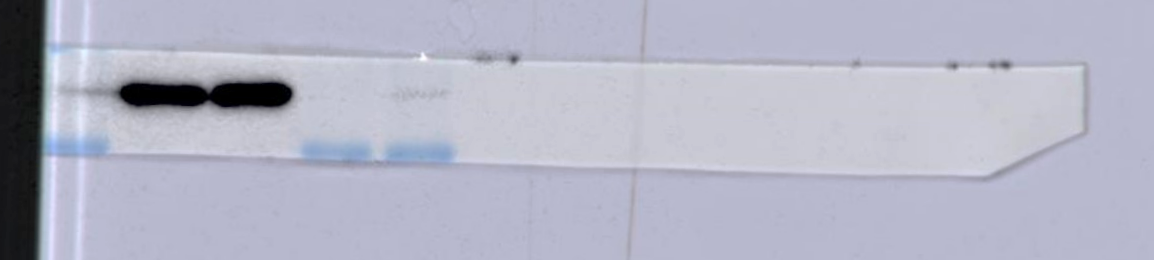
**

**p-AKT(Ser473):**

**
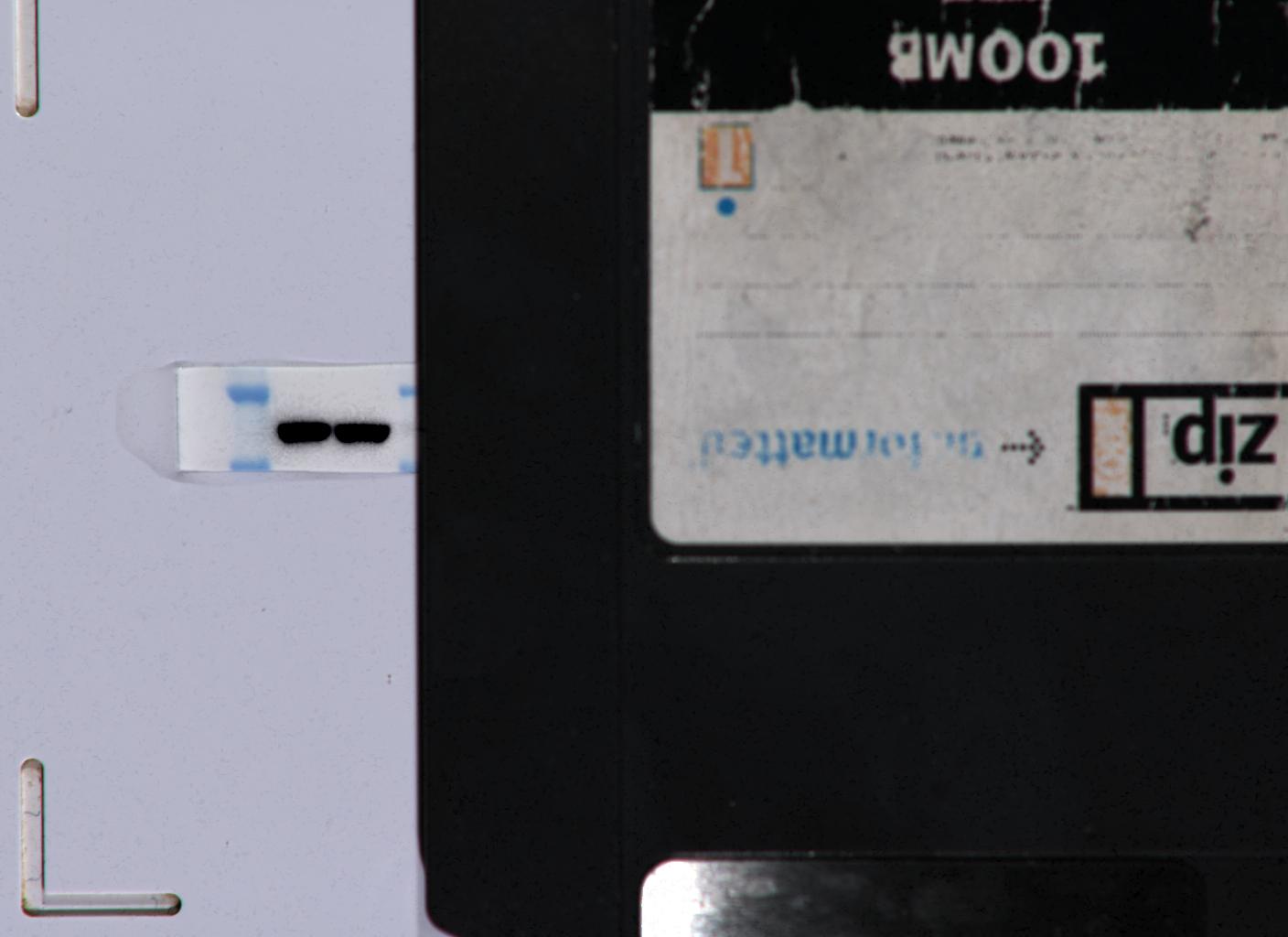

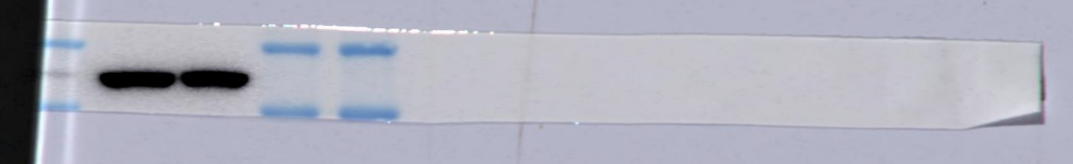
**

**AKT:**

**
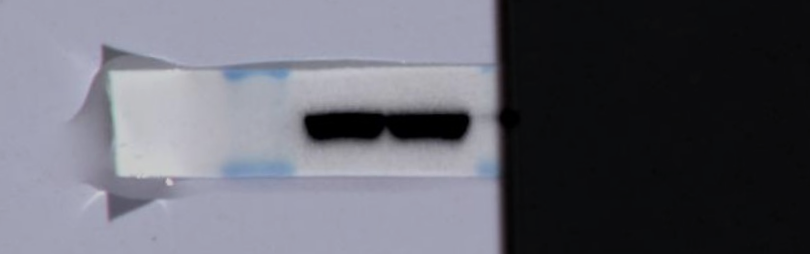

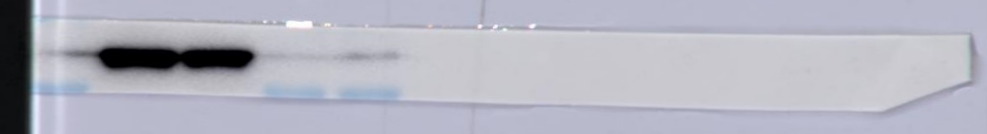
**

**p-ERK:**

**
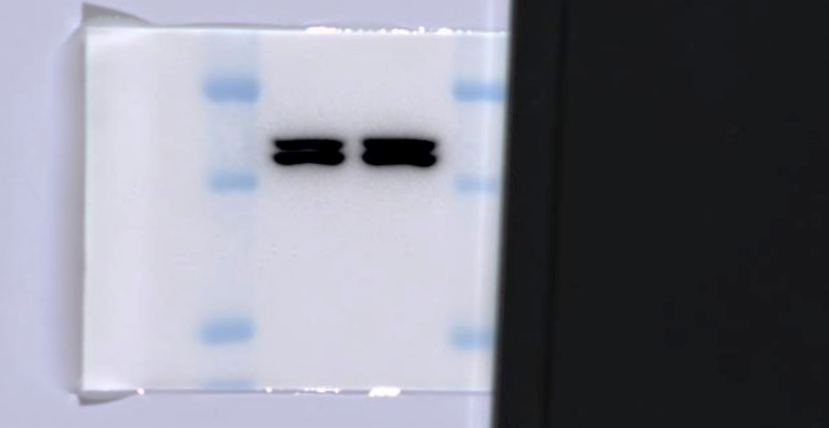

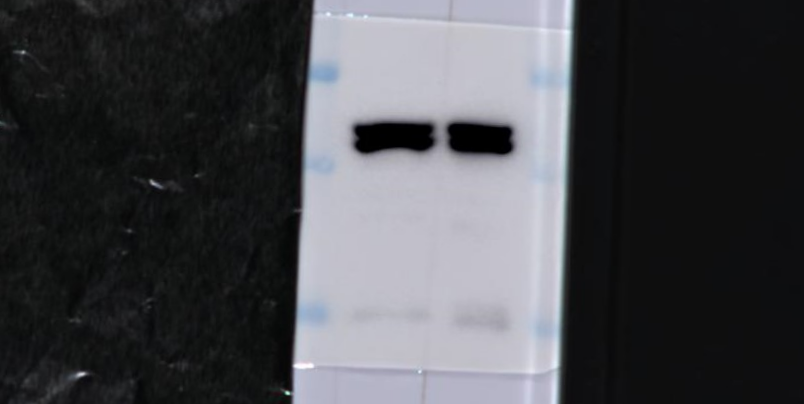
**

**ERK:**

**
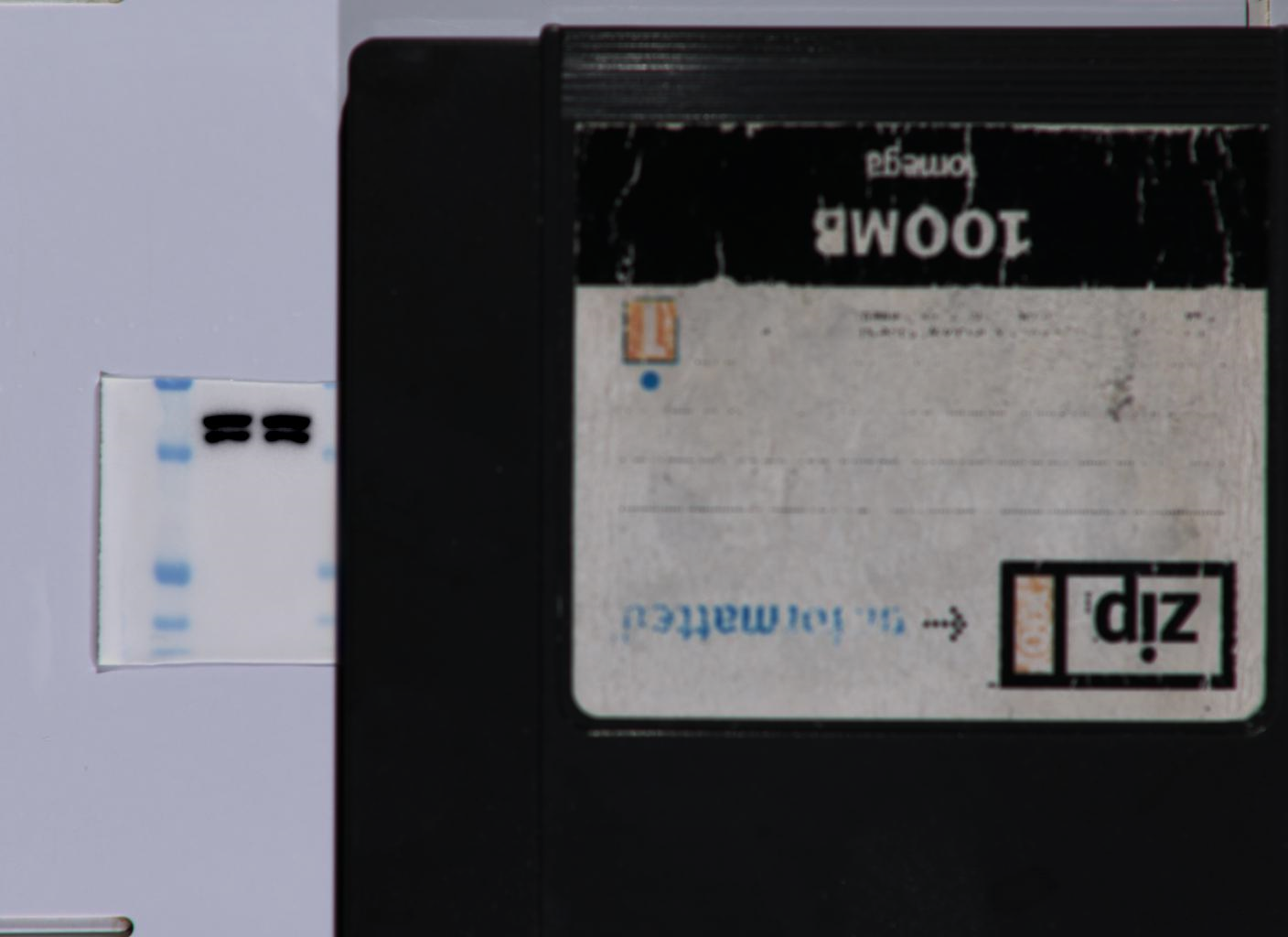

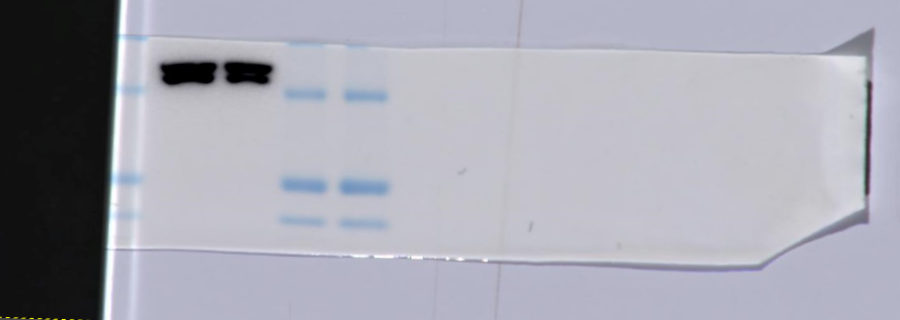
**

**Beta-actina:**

**
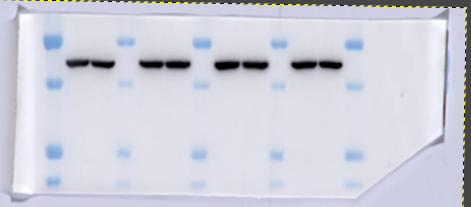
**
